# *Talaromyces neofusisporus* and *T. qii,* two new species of section *Talaromyces* isolated from plant leaves in Tibet, China

**DOI:** 10.1038/srep18622

**Published:** 2016-01-04

**Authors:** Qi-Ming Wang, Yong-Hong Zhang, Bo Wang, Long Wang

**Affiliations:** 1 State Key Laboratory of Mycology, Institute of Microbiology, Chinese Academy of Sciences, Beijing 100101, China; 2 College of Animal Science and Technology, Beijing University of Agriculture, Beijing 102206, China; 3 State Key Laboratory of Crop Stress Biology in Arid Areas and College of Plant Protection, Northwest A&F University, Yangling 712100, Shaanxi, China

## Abstract

Two new species isolated from plant leaves belonging to *Talaromyces* section *Talaromyces* are reported, namely *T. neofusisporus* (ex-type AS3.15415 ^T^ = CBS 139516 ^T^) and *T. qii* (ex-type AS3.15414 ^T^ = CBS 139515 ^T^). Morphologically, *T. neofusisporus* is featured by forming synnemata on CYA and YES, bearing appressed biverticillate penicilli and smooth-walled fusiform conidia about 3.5–4.5 × 2–2.5 μm; and *T. qii* is characterized by velutinous colony texture, yellowish green conidia, yellow mycelium and ovoid to subglobose echinulate conidia measuring 3–3.5 μm. Phylogenetically, *T. neofusisporus* is such a unique species that no close relatives are found according to *CaM, BenA* and *ITS1-5.8S-ITS2* as well as the combined three-gene sequences; and *T. qii* is related to *T. thailandensis* according to *CaM, BenA* and the combined sequence matrices, whereas *ITS1-5.8S-ITS2* sequences do not support the close relationship between *T. qii* and *T. thailandensis*.

The species bearing symmetrical biverticillate penicilli, acerose phialides, with mycelium showing yellow, orange, pink or red tints, and the ascocarps, when present, being gymnothecial had been included in *Penicillium* section *Biverticillata-Symmetrica* by Raper and Thom^1^. Due to the rules of dual nomenclature, Pitt^2^ discriminated the anamorphic state from the teleomorphic state and placed the species only presenting anamorphic state in *Penicillium* subgenus *Biverticillium* and those with the teleomorphic state in the genus *Talaromyces*. However, the intrinsic difference between the species showing the above characters and other penicillia had long been well evidenced either by traditional characters [e. g.,^3^] or molecular phylogenetics [e. g.,^4^,^5^,^6^,^7^]. Recently, the Melbourne nomenclatural code abolished dual naming system and decided using a single name for a single species^8^, thus*Talaromyces* became the valid genus name for these species. In 2011, Samson *et al.* accepted 71 species in *Talaromyces*^7^. Later in 2012, Visagie and Jacobs established 3 new species^9^. In 2013, Manoch *et al.* reported 2 new members isolated from Thailand^10^. Then Peterson and Jurjević added another new member to the genus^11^. Afterwards, Sang *et al.* described 2 new taxa from Korea^12^ and Frisvad *et al.* reported a distinct species producing red pigment^13^. This year, Yilmaz *et al.* discovered 4 new members of the genus^14^. In a monographic study, Yilmaz *et al.* listed 88 species and divided *Talaromyces* into 7 sections, i. e. sections *Talaromyces*, *Helici*, *Purpurei*, *Trachyspermi*, *Bacillispori*, *Subinflati* and *Islandici*, among which, section *Talaromyces* included 36 species^15^. Just recently, Visagie *et al.* added 5 new members to this section^16^.

In the survey of phylloplane moulds in China, we discovered certain isolates showing the characters of the genus *Talaromyces*. Here, we report 2 additional new taxa of section *Talaromyces*, namely *T. neofusisporus* sp. nov. and *T. qii* sp. nov.

## Results and Discussion

PCR amplification produced amplicons of the partial calmodulin gene (*CaM*) ca. 660 bp, partial β-tubulin gene (*BenA*) about 650 bp using primers I2 and Bt2b, and ca. 410 bp using primers Bt2a and Bt2b, the ITS1-5.8S-ITS2 region of the rDNA (*ITS1-5.8S-ITS2)* about 540 bp. The trimmed alignments of *CaM*, *BenA*, *ITS1-5.8S-ITS2* and the combined three-gene sequences contained 553, 416, 459 and 1432 characters with gaps, respectively.

**Figure 1:**
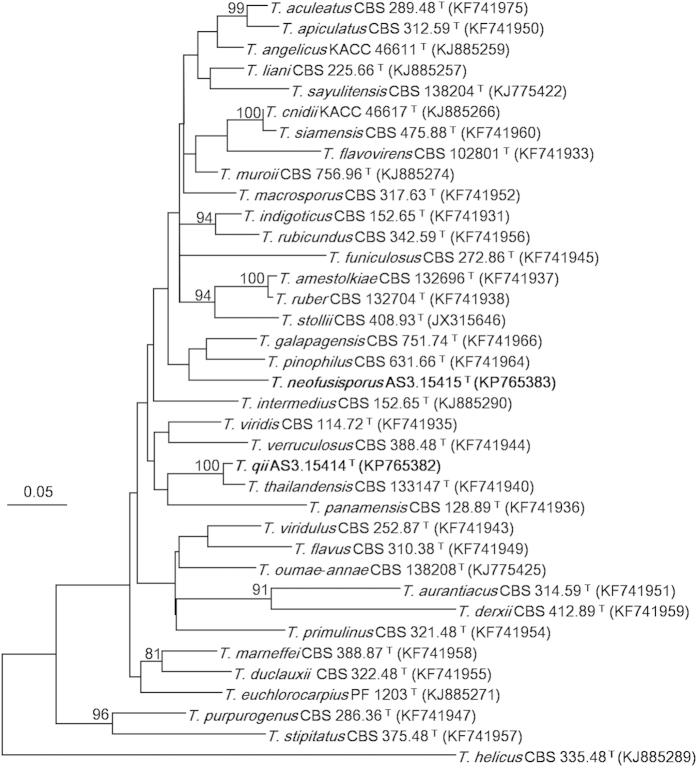
ML phylogram inferred from partial *CaM* sequences. Bootstrap percentages over 70% derived from 1000 replicates are indicated at the nodes. Bar = 0.05 substitutions per nucleotide position.

**Figure 2:**
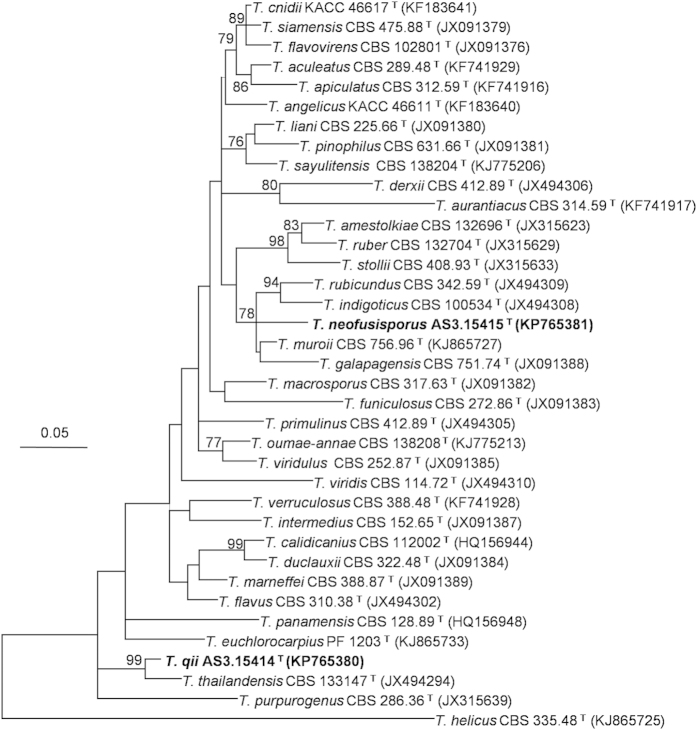
ML phylogram inferred from partial *BenA* sequences. Bootstrap percentages over 70% derived from 1000 replicates are indicated at the nodes. Bar = 0.05 substitutions per nucleotide position.

**Figure 3:**
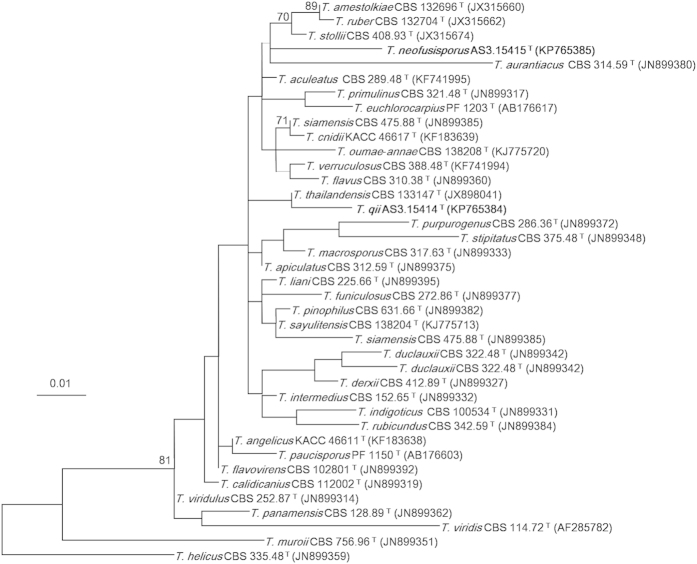
ML phylogram inferred from partial *ITS1-5.8S-ITS2* sequences. Bootstrap percentages over 70% derived from 1000 replicates are indicated at the nodes. Bar = 0.01 substitutions per nucleotide position.

The Maximum Likelihood (ML) phylograms resulting from *CaM*, *BenA, ITS1-5.8S-ITS2* and the three-gene matrices all showed that *T. neofusisporus* was a unique species without close relatives; and *T. qii* was closely related to *T. thailandensis* with 100%, 99% and 99% bootstrap support according to *CaM, BenA* and the three-gene sequences, respectively. However in the phylogram based on *ITS1-5.8S-ITS2* region, *T. qii* had no close relatives. On the whole, either the individual or the combined analyses of the three genes supported *T. neofusisporus* and *T. qii* as valid new species (Figs 1, 2, 3, Supplementary Figure S1).

### Description of *Talaromyces neofusisporus* L. Wang, sp. nov

MycoBank: MB 811447

**Figure 4:**
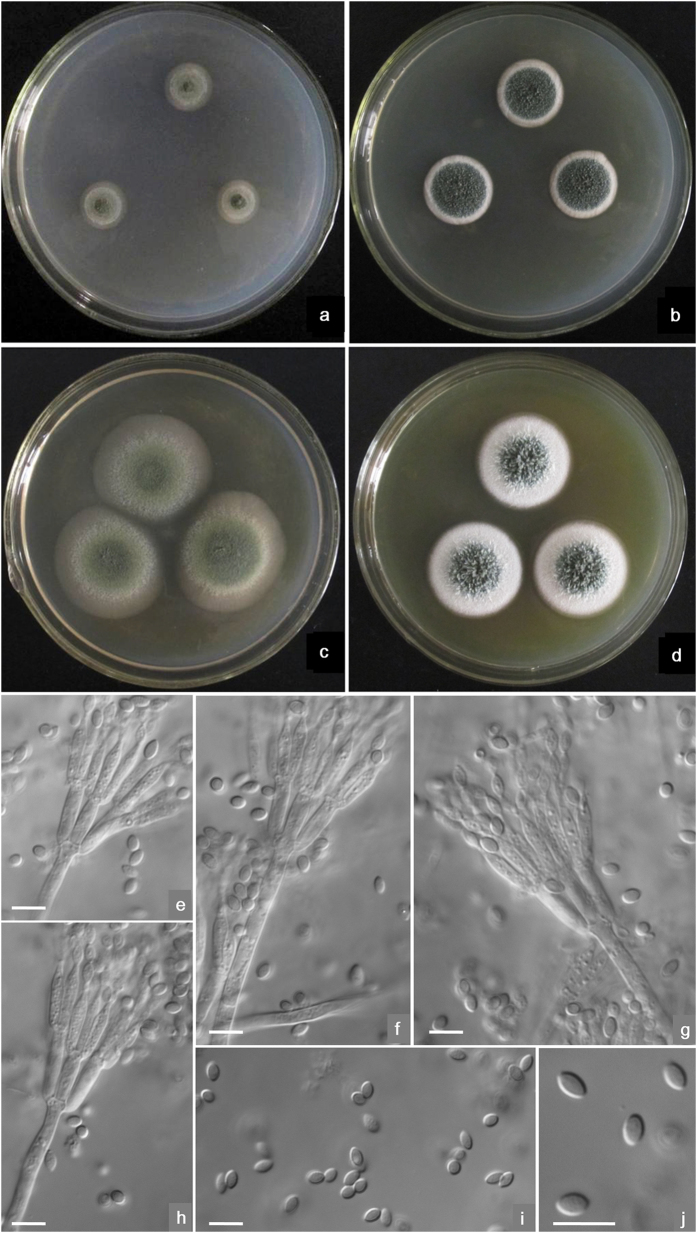
Morphological characters of *T. neofusisporus* AS3.15415 ^T^. (**a**) On Cz at 25 °C after 7 d; (**b**) On CYA at 25 °C after 7 d; (**c**) On MEA at 25 °C after 7 d; (**d**) On YES at 25 °C after 7 d; (**e–h**) Conidiophores; (**i–j**) Conidia. Bar = 10 μm.

Etymology: The specific epithet is derived from the fusiform-shaped conidia of this species.(Fig. 4)

Holotype: HMAS246033

On **Cz** at 25 °C after 7 d: Colonies 13–14 mm diam, plane, low, sparse, margins submerged; velutinous; conidiogenesis moderate at central areas, coloured near Grayish Olive (R. Pl. XLVI); mycelium white; no exudate and soluble pigment; reverse coloured Pale to Light Grayish Olive (R. Pl. XLVII). On **CYA** at 25 °C after 7 d: Colonies 19–20 mm diam, plane, low; surface appearing velutinous and granular due to synnemata about 1–2 mm long; conidiogenesis abundant, near Russian Green (R. Pl. XLII); mycelium white; no exudate and soluble pigment; reverse coloured near Cream Color (R. Pl. XVI). On **MEA** at 25 °C after 7 d: Colonies 33–36 mm diam, low, plane, margins submerged; velutinous; conidiogenesis abundant, near Deep Dull Yellow-Green (1) (R. Pl. XXXII); mycelium white; no exudate and soluble pigment; reverse coloured near Naphthalene Yellow (R. Pl. XLI). On **YES** at 25 °C after 7 d: Colonies 26–28 mm diam, loose and deep; mycelium white, aggregated into synnemata about 2–3 mm long in central areas; conidiogenesis moderate at central areas, near Russian Green (R. Pl. XXXII); mycelium white; no exudate and soluble pigment; reverse coloured Ochraceous-Buff to Light Ochraceous-Buff (R. Pl. XV). No growth at **5** °**C** on CYA. On CYA at **37 **°**C** after 7 d, colonies 2–3 mm diam with white mycelium only.

Conidiophores arising from agar surface and synnemata; stipes 120–180 (−200) × 3–3.5 μm when from surface, but 85–120 μm when from synnemata, smooth-walled; penicilli biverticillate; appressed metulae 4–6 (−8) per stipe, 9 –11 × 2.5–3 μm; phialides 2–4 per metula, acerose with distinguishable collula, 9–11 × 2.5–3 μm; conidia fusiform, (3.5−) 4–4.5 (−5) × 2–2.5 μm, smooth-walled, born in irregularly tangled chains about 120 μm forming loose brushes. Teleomorphic state unknown.

Strains examined. CHINA. Tibet: Motuo County, 29°41′37″N 94°43′36″E, 3700 m; ex-type culture AS3.15415 ^T^ = CBS 139516 ^T^ from an unidentified leaf sample no. *150C6*, 19 Sep 2014, *Q-M. Wang.* (HOLOTYPE: HMAS 246033, Institute of Microbiology, Chinese Academy of Sciences, Beijing, China; dried culture of ex-type AS3.15415 ^T^ on Cz).

Notes. *T. neofusisporus* is characterized by synnemata, fusiform conidia, and the growth at 37 °C.

### Description of *Talaromyces qii* L. Wang, sp. nov

MycoBank: MB 811448

**Figure 5:**
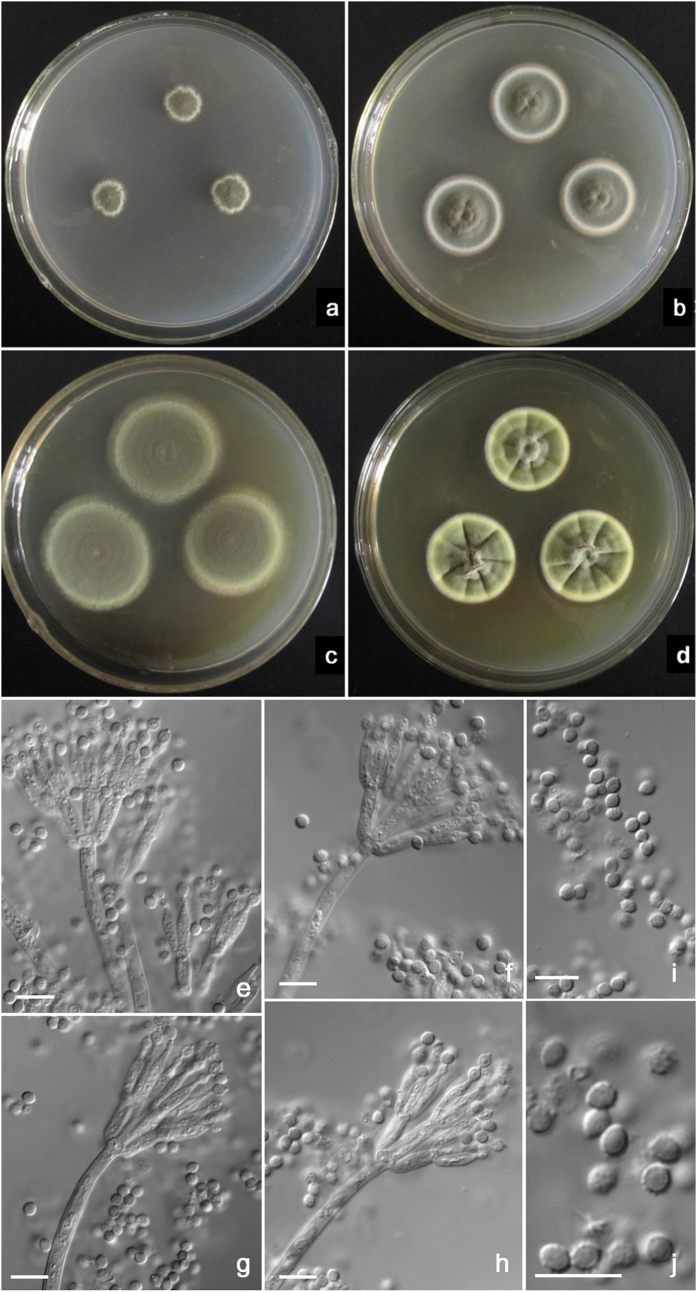
Morphological characters of *T. qii* AS3.15414 ^T^. (**a**) On Cz at 25 °C after 7 d; (**b**) On CYA at 25 °C after 7 d; (**c**) On MEA at 25 °C after 7 d; (**d**) On YES at 25 °C after 7 d; (**e–h)** Conidiophores; (**i–j**) Conidia. Bar = 10 μm.

Etymology: The specific epithet is in honour of Prof. Zu-Tong Qi, who made great contribution to the *Aspergillus* and *Penicillium* taxonomy in China. (Fig. 5)

Holotype: HMAS246032

On **Cz** at 25 °C after 7 d: Colonies 10–13 mm diam, thin, plane, margins irregular, submerged; velutinous; conidiogenesis abundant, coloured near Dark Dull Yellow-Green (R. Pl. XXXII) or Light Hellebore Green (R. Pl. XVII); mycelium coloured near Pale Dull Green-Yellow (R. Pl. XVII); no exudate and soluble pigment; reverse coloured Orange in central areas and Maize Yellow at marginal areas (R. Pl. XV). On **CYA** at 25 °C after 7 d: Colonies 23–24 mm diam, thin, plane but protuberant centrally with slightly radial and annular plicates; margins regular and submerged; velutinous; conidiogenesis abundant, near Dark Dull Yellow-Green (R. Pl. XXXII); mycelium coloured near Pale Dull Green-Yellow (R. Pl. XVII); no exudate and soluble pigment; reverse coloured near Hay’s Russet (R. Pl. XIV), with a Light Ochraceous-Salmon tint at marginal areas (R. Pl. XV). On **MEA** at 25 °C after 7 d: Colonies 33–35 mm diam, low, plane, margins regular and submerged; velutinous; conidiogenesis abundant, near Pois Green (R. Pl. XLI); mycelium with a Chalcedony Yellow tint (R. Pl. XVII); no exudate and soluble pigment; reverse coloured near Deep Colonial Buff (R. Pl. XXX). On **YES** at 25 °C after 7 d: Colonies 25–27 mm diam, low, slightly with radial plicates, protuberant in centers, margins regular; velutinous, conidiogenesis abundant, Pois Green (R. Pl. XLI); mycelium with a Clear Yellow-Greeen (R. Pl. VI) tint; no exudate and soluble pigment; reverse coloured near Hay’s Russet (R. Pl. XIV) with Ochraceous-Buff to Light Ochraceous-Buff colour at margins. No growth at **5 °C** and **37 °C** on CYA.

Conidiophores arising from substrate; stipes (150–) 200–300 (−360) × 3.5–4 μm, smooth-walled; with biverticillate penicilli; metulae 4–6 (−8) per stipe, 7–11 (−13) × 2.5–3 μm; phialides 2– 4 per metula, acerose to ampulliform with short collula, 7–9 × 2–2.5 (−3) μm; conidia ovoid to suglobose, 3–3.5 μm, walls echinulate, born in irregularly tangled chains forming loose masses about 120 μm. Teleomorphic state unknown.

Strains examined. CHINA. Tibet: Motuo County, 29°16′30″N 95°15′04″E, 1211 m; ex-type culture AS3.15414 ^T^ = CBS 139515 ^T^ from an unidentified leaf sample no. *125E25*, 19 Sep 2014, *Q-M. Wang.* (HOLOTYPE: HMAS 246032, Institute of Microbiology, Chinese Academy of Sciences, Beijing, China; dried culture of ex-type AS3.15414 ^T^ on Cz).

Notes. *T. qii* is characterized by the velvety colony texture, yellow-coloured mycelium and ovoid to subglobose echinulate conidia.

*T. neofusisporus* and *T. qii* are located in *Talaromyces* section *Talaromyces*, which including 41 species according to the most recent phylogenetic studies by Yilmaz *et al.* and Visagie *et al.*^15^,^16^.

Yilmaz *et al.* reported 12 species that can produce synnemata in the genus *Talaromyces*, and that only 4 members belong to section *Talaromyces*, namely *T. calidicanius*, *T. duclauxii*, *T. flavovirens*, and *T. panamensis*^15^. In this paper, another synnema-producing member is added, i. e. *T. neofusisporus*. In addition to the phylogenetic evidence of Figs 1, 2, 3 and Supplementary Figure S1 indicating that *T. neofusisporus* is not related to the above 4 species, this new taxon can also be readily distinguished from them morphologically.

*T. neofusisporus* can be distinguished from *T. calidicanius* in that it grows slowly on standard media at 25 °C (Cz 13–14 mm, CYA 19–20 mm, MEA 33–36 mm, YES 26–28 mm) showing the synnematous colony texture only on CYA and YES, bears exclusive appressed biverticillate penicilli with smooth-walled fusiform conidia. However, *T. calidicanius* grows faster (CYA 27–30 mm, MEA 47–48 mm, YES 40–41 mm), presenting funiculose and floccose texture with long synnemata about 6 mm on all standard media; additionally, it produces a minor portion of biverticillate penicilli bearing subterminal branches, and rough-walled to striate-walled conidia^15^,^17^.

*T. duclauxii* is also a species that produces long synnemata up to 5 mm within 7 days on all standard media, giving a deep fluffy colony appearance; it also bears a portion of biverticillate penicilli with subterminal branches on sinuous stipes, and ellipsoidal to subglobose, smooth to finely rough-walled conidia. However, *T. neofusisporus* only produces discernible synnematous colony texture on CYA and YES, and shows velvety texture on Cz and MEA; besides, it produces strictly biverticillate penicilli and straight conidiophore stipes, with smooth-walled fusiform conidia^1^,^2^,^15^.

Although the characters of low growth rate, velvety colony appearance on Cz and MEA, and abundant sporulation fairly resemble those of *T. flavovirens*, *T. neofusisporus* bears relatively long synnemata about 2–3 mm on YES that are obvious to the naked eye within 7 days, with no gymnothecia observed neither in nature nor on artificial media, yet *T. flavovirens* bears shorter synnemata only 750 μm in length after prolonged culturing and produces ascomata on *Quercus suber* leaf litter according to Visagie *et al.* Moreover, the mycelium of *T. neofusisporus* is in white colour, while that of *T. flavovirens* is with a yellow tint. Furthermore, the penicilli are strictly biverticillate and conidia are fusiform-shaped in *T. neofusisporus*, but in *T. flavovirens* a minor portion of biverticillate penicilli with subterminal branches and ellipsoidal conidia are observed^15^,^18^.

The low growth rate, fusiform conida and grayish-coloured conidia *en masse* of *T. neofusisporus* are similar to those of *T. panamensis*. However, *T. neofusiporus* gives a moderate to abundant sporulation on culturing media, does not form synnemata on MEA, but instead bears obvious synnemata on YES without visible yellow stalks. On the contrary, *T. panamensis* sporulates sparsely on all media, does not produce synnemata on YES, while produces yellow-stalked synnemata on MEA. Furthermore, *T. neofusisporus* bears only biverticillate penicilli on relatively longer stipes commonly about 85–120 μm even from synnemata, whereas *T. panamensis* produces a minor portion of biverticillate penicilli with subterminal branches on shorter stipes ca. 40–85 μm^15^,^19^.

In the phylograms inferred from *CaM*, *BenA* and the combined three-gene sequences, *T. qii* is closely related to *T. thailandensis* with 100%, 99% and 99% bootstrap support, respectively. However the phylogram based on *ITS1-5.8S-ITS2* does not support a close relationship between them, because though they fall in the same clade, there is no significant bootstrap support for this clade (Fig. 3). In addition, *T. qii* and *T. thailandensis* can be readily distinguished from each other morphologically in that *T. thailandensis* produces yellow gymnothecia, ellipsoidal spiny ascospores, but no sexual state is found in *T. qii*. Additionally, *T. qii* grows more slowly (CYA 23–24 mm, MEA 33–35 mm, YES 25–27 mm) than *T. thailandensis* (CYA 45–47 mm, MEA 40–42 mm, YES 47–50 mm), and produces abundant echinulate conidia on all the standard media, while the sporulation of *T. thailandensis* is almost absent or sparse and its conidia are smooth-walled^10^,^15^.

The moderate growth rate at 25 °C and no growth at 37 °C, as well as the ovoid to subglobose rough-walled conidia of *T. qii* indicate certain similarities to *T. kendrickii*. But *T. qii* shows a velvety colony texture with heavy sporulation and without exudate, while in *T. kendrickii*, the colony texture is floccose, the sporulation is sparse and abundant reddish to pinkish exudate is obviously present. Moreover in micromorphology, *T. qii* bears only biverticillate penicilli, but some monoverticillate penicilli are present in *T. kendrickii*. Further, the stipe lengths of *T. qii* are shorter (150–360 μm) than those of *T. kendrickii* (150–500 μm), and *T. qii* produces larger conidia (3–3.5 μm) than those of *T. kendrickii* (2.5–3 μm).

The characters of the plane dense colonies, heavy sporulation, conidia *en masse* coloured dull green with a yellowish green tint of *T. qii* remind of the resemblance to *T. diversus* and *T. cnidii*. However, *T. qii* grows much faster on CYA (23–24 mm) and YES (25–27 mm) than *T. diversus* on CYA (7–10 mm) and YES (8–10 mm). Besides, *T. qii* does not grow at 37 °C, while *T. diversus* can form colonies about 2–8 mm in diam. Still, the penicilli are strictly biverticillate in *T. qii*, but *T. diversus* bears a portion of biverticillate penicilli with subterminal branches. In addition to these morphological differences, the phylogenetic work of Yilmaz *et al.* indicates that *T. diversus* is a member of section *Trachyspermi*, which is phylogenetically fairly distant to section *Talaromyces* where *T. qii* is located^1^,^2^,^15^. Comparing with *T. cnidii*, the growth rate of *T. qii* is slower (CYA 23–24 mm, MEA 33–35 mm, YES 25–27 mm) than that of *T. cnidii* (CYA 30–35 mm, MEA 38–43 mm, YES 40–45 mm), and *T. qii* does not excrete soluble pigment on CYA, while *T. cnidii* produces strong red pigment. Additionally, the conidia of *T. qii* are ovoid to subglobose with echinulate walls, whereas those of *T. cnidii* are ellipsoidal with smooth to finely rough walls^12^,^15^. Besides the morphological disparity, our phylograms resulted from *CaM*, *BenA*, *ITS1-5.8S-ITS2* and the combined sequences all demonstrate that *T. qii* and *T. cnidii* are located in different clades in section *Talaromyces* (Figs 1, 2, 3, Supplementary Figure S1).

The characters of similar growth rate, velvety colony texture, yellowish dull green conidia *en masse* and yellowish mycelium also present certain resemblance between *T. qii* and *T. ruber*. However, the colony reverse of *T. qii* on Cz and CYA shows a tint of orange colour but that of *T. ruber* coloured cherry red or brownish red. Besides, the conidia of *T. qii* are ovoid to subglobose with echinulate walls, but those of *T. ruber* are ellipsoidal and smooth-walled^1^,^15^. In addition to the morphological evidence, our molecular phylograms inferred separately from the three loci and the combined sequences all indicate that *T. qii* and *T. ruber* lie in well-separated clades (Figs 1, 2, 3, Supplementary Figure S1).

Plant phylloplane is one major natural habitat for bacteria, yeasts and filamentous fungi^20^,^21^. The phylloplane microbial species are recruited from the airborne taxa in the environment and then a distinct phylloplane microbial community is constructed. During this process, plant genotypes play an important role as the selection force by providing specific nutrients and habitats^22^,^23^,^24^. Thus, more new or distinctive microbial taxa might be discovered from phyllosphere where the plant diversity is abundant. Correspondingly, habitant microbes produce growth hormones for host plants and assist host plants in the antagonism against pathogens in many respects^25^,^26^. In this sense, the discovery of new phylloplane taxa may provide potential biocontrol agents.

## Materials and Methods

### Isolation of strains

Leaf samples from living trees were collected and kept in sterilized plastic bags and reserved in a mobile refrigerator. The isolation of phylloplane fungi followed the method of Nakase and Takashima^27^. No specific permissions were required for these locations/activities. The field studies did not involve endangered or protected species and the GPS coordinates of the specific locations in our study are 29°41′37″N 94°43′36″E and 29°16′30″N 95°15′04″E. The isolates were deposited in China General Microbiological Culture Collection (CGMCC), Institute of Microbiology, Chinese Academy of Sciences, Beijing, China. The ex-type cultures of *T. neofusisporus* AS3.15415 ^T^ and *T. qii* AS3.15414 ^T^ were also deposited in the culture collection of the CBS-KNAW Fungal Biodiversity Centre in The Netherlands as CBS 139516 ^T^ and CBS 139515 ^T^, respectively. The cultures are also reserved at the corresponding author’s laboratory and will be supplied upon request for educational or scientific purpose.

### Morphological studies

Colony characters were examined using Czapek agar (Cz, Raper and Thom^1^), Czapek yeast autolysate agar (CYA, Pitt^2^), 2% malt extract agar (MEA, malt extract (Difco), Pitt^2^), YES (yeast extract sucrose agar, yeast extract (Oxoid), Frisvad and Samson^28^). Colour names were referred to Ridgway^29^. Wet mounts were made using material from colonies growing on MEA at 25 °C after 7 d and mounted in 85% lactic acid without dye. The microscopic characters were examined and photographed using Axioplan2 imaging and Axiophot2 universal Microscope (Carl Zeiss Shanghai Co. Ltd.).

### Molecular studies

Genomic DNA extraction method was referred to Scott *et al.*^30^. *BenA* sequences were amplified with the sense primers btI2^31^ or Bt2a, and the antisense primer Bt2b^32^; *ITS1-5.8S-ITS2* sequences were obtained using the primers ITS5 and ITS4^33^; the *CaM* PCR amplification was achieved with the primers AD1 and Q1^34^. PCR reaction was employed in 20 μL reaction mixture containing 0.5 μL of each primer (10 pM/μL), 1.0 μL of genomic DNA (10 ng/μL), 8 μL of 2 × PCR MasterMix buffer (0.05 u/μL Taq polymerase, 4 mM MgCl_2_, 0.4 mM dNTPs), and 10 μL of double-distilled water (Tsingke Biotechnologies Co., Ltd., Beijing, China). Amplifications were performed in a PTC-150 thermocycler (MJ Research, Watertown, Massachusetts, USA), which was programmed for 3-min. denaturation at 94 °C followed by 34 cycles of 94 °C for 30 s, 50 °C for 30 s and extension for 45 s at 72 °C, with a final 5 min. elongation step at 72 °C. After amplification, the PCR fragments were electrophoresed in 2.0% agarose gels with a 100 bp DNA ladder (MBI Fermentas) at 80 V for 20 min. Gels were stained in 0.5 μg/mL ethidium bromide water solution for 15 min and checked under 254 nm UV with a portable UV light. Samples showing one clear, single band of the anticipated length on the gel were then purified and sequenced in double direction with an ABI 3700 DNA analyzer (Applied Biosystems, Inc., Foster City, California, USA). Raw sequences were proof-read and edited manually with BioEdit 7.0.9^35^. Edited sequences were aligned using muscle implemented in MEGA version 5^36^. The new species together with 34 ex-type strains without *T. calidicanius* and *T. paucisporus* were included in *CaM* analysis; with 34 ex-type isolates of section *Talaromyces* without *T. paucisporus* and *T. stipitatus* were in *BenA* analysis; and with 36 ex-type cultures were subjected to *ITS1-5.8S-ITS2* analysis; while with 33 ex-type isolates lacking *T. calidicanius*, *T. paucisporus* and *T. stipitatus* were in the combined three-gene analysis. *T. helicus* of section *Helici*^15^ was as the outgroup in all these analyses (Figs 1, 2, 3, Supplementary Figure S1). First, these sequence data were determined for the most optimal substitution model and the rates among sites using the function “Find the Best DNA/Protein Models (ML)” of MEGA version 5, and Kimura-2 parameter substitution model and Gamma distributed with Invariant sites (G + I) for rates among sites were found to be the best, gaps were treated as partial deletion according to Hall^37^. All the sequence matrices were analyzed using the ML method and subjected to 1000 bootstrap replications.

## Additional Information

**How to cite this article**: Wang, Q.-M. *et al.* *Talaromyces neofusisporus* and *T. qii*, two new species of section *Talaromyces* isolated from plant leaves in Tibet, China. *Sci. Rep.* **6**, 18622; doi: 10.1038/srep18622 (2016).

## Supplementary Material

Supplementary Information
