# Supplementary material for: Talaromyces neofusisporus and T. qii, two new species of section Talaromyces isolated from plant leaves in Tibet, China
Source: Sci Rep. 2016 Jan 4;6:18622. doi: 10.1038/srep18622 (PMC4698637; doi:10.1038/srep18622)
Supplement: Supplementary Information [file srep18622-s1.pdf]

Talaromyces fusisporus and _T. qii_, two new species of section _Talaromyces_ isolated from plant leaves in Tibet, China

Qi-Ming Wang, Yong-Hong Zhang, Bo Wang, Long Wang

Figure S1. ML phylogram inferred from the combined *CaM*, *BenA* and *ITS1-5.8S-ITS2* sequences. Bootstrap percentages over 70% derived from 1000 replicates are indicated at the nodes. Bar = 0.05 substitutions per nucleotide position.

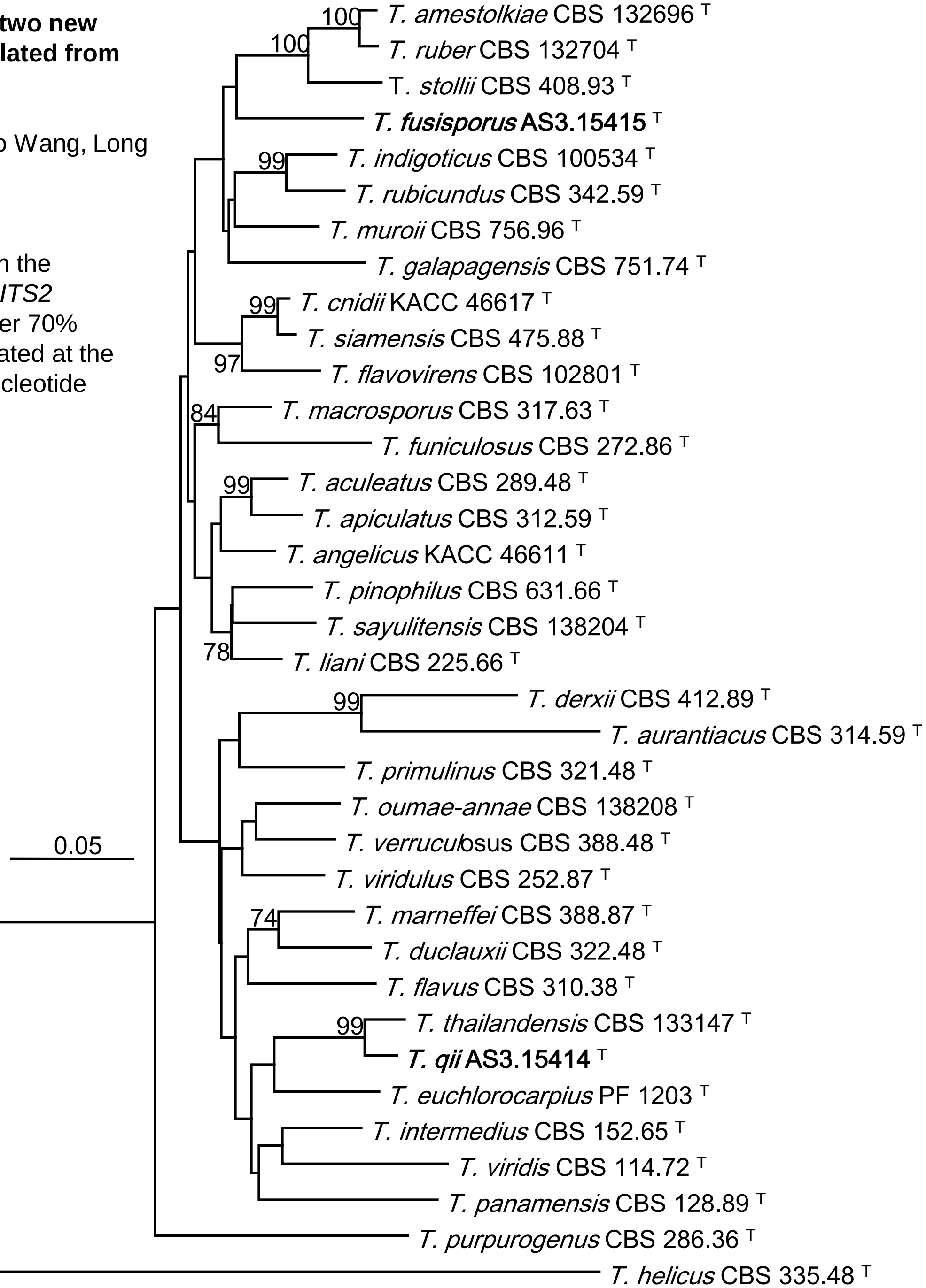

Supplementary dataset 1

>KF741975_Talaromyces_aculeatus_ CBS 289.48 cmd

TG-GATATCTAT-TTGT--C--------GCAATGTTG---TGG-TGG--GTGGTTAGCTGACTA---CGGTTTGATG-AGTAGGACA

AGGATGGAGATGGTGAG---TCCGC-----CACGAA-C-AATAAC---------CAAA-AGACCTTGACC-GAAG------GTGATCG

C--------AACGG-----ACAA-ATATTG------ATAAAATT-GAA-----------------TAGGCCAAATCACAACCAAGGAACTGGGCA

CCGTCATGCGCTCTCTCGGCCAGAACCCCTCCGAATCCGAATTGCAGGACATGATCAACGAAGTCGACGCTGAC

AACAACGGCACAATCGATTTCCCTGGTATGCTATAAA--------TCCT---TACGGGTTATTACG-----------------ATGGC---G

G-TACTAACTGC--T----G-CAGAATTCTTGACAATGATGGCCCGCAAAATGAAGGATACCGACTCCGAGGAAGAGAT

CCGCGAGGCTTTCAAGGTGTTTGACCGTGACAACAATG

>KF741937_Talaromyces_amestolkiae CBS 132696

TG-GAAACTTGGTTTGT--C--------ACAATGTTG--------GC--GTGGTTAGCTGACCAG-CCGTTTTGATG-AATAGGACA

AGGATGGAGATGGTGAG----TCGC-----CGCGAA-C-AATAAA-------------ACACCTTGAAC-GAAC------GTTACCGC----

----AGCCA-----ACAG-ACATTG------ACCCTATC-GGA-----------------CAGGACAAATCACAACCAAGGAACTCGGCACA

GTCATGCGTTCTCTCGGCCAGAACCCCTCCGAATCCGAATTGCAGGACATGATCAACGAAGTCGACGCTGACAA

CAACGGCACAATTGATTTCCCTGGTATGACTGACCACC-----AACT---CGCAATATTTG--------------------ATGGC---AG-

TACTGACTGC--C----G-CAGAATTCTTGACAATGATGGCCCGCAAAATGAAGGATACCGACTCCGAGGAAGAGATC

CGTGAGGCTTTCAAGGTGTTTGATCGTGACAACAATG

>KJ885259_Talaromyces_angelicus KACC 46611

TG-GATTTCCAC-TTGT--C--------GTAATGGTG---------G--GTGGTTAGCTGACTAG--CGTTTTGATG-AGTAGGACAAG

GATGGAGATGGTGAG---TCCGC-----CACGAA-C-GATAAC---------CAAA-AGACCTTGAAC-AAAG------GTTATCGC----

----TACGA-----ACAG-ATATTG------ACTATATC-GAA-----------------TAGGCCAAATCACGACCAAGGAACTGGGCACCG

TCATGCGTTCCCTCGGCCAGAACCCCTCCGAATCCGAATTGCAGGACATGATCAACGAAGTCGACGCTGACAAC

AACGGCACAATCGATTTTCCTGGTATGATATAAT--------TGCT---TACGGTTTACTACG-----------------ATGGC---AG-TAC

TAACTGC--C----G-CAGAATTCTTGACAATGATGGCCCGCAAAATGAAGGATACCGACTCCGAGGAAGAGATCCGC

GAGGCTTTCAAGGTGTTTGACCGCGACAACAATG

>KF741950_Talaromyces_apiculatus CBS 312.59

TG-GATATCTAT-TTGT--C--------GCAATGTTG---CCG-TGG--GTGGTTAGCTGACTAG--CGGCTTGATG-AGTAGGAC

AAGGATGGAGATGGTGAG---TCCGC-----CATGAA-C-AATAAC---------CAAG-AGACCTTGAAC-ATAG------GTGATCG

C--------AACGA-----ACAA-GTATTG------ATAAAATC-GAA-----------------TAGGCCAAATCACAACCAAGGAACTGGGCA

CCGTCATGCGTTCTCTCGGCCAGAACCCCTCCGAATCCGAATTACAGGACATGATCAATGAGGTCGACGCTGACA

ACAACGGCACAATCGATTTCCCTGGTATGATATAAT--------TGCT---TACGGGTTATTACG-----------------ACGGC---AG-

TGCTAACTGC--C----G-CAGAATTCTTGACAATGATGGCCCGCAAAATGAAGGATACCGACTCCGAGGAAGAGATC

CGCGAGGCTTTCAAGGTGTTTGACCGTGACAACAATG

>KF741951_Talaromyces_aurantiacus CBS 314.59

TGAAATATCTTC-CTGTCGC--------ATAGTATTT-------T----CTCGTT-GCTTACAGA-ATGTTTTAATGAAATAGGACAAG

GATGGAGATGGTGAGTG-ACGAC-----TGCGACAC-ACCAAC---------GATGTCGTATCGAAAGCAGATTCATCTACGA

TC----------TACGA-----ATAA-ATATTG------ATAGAGTC-GGA-----------------CAGGTCAAATCACAACCAAGGAACTGGG

CACCGTCATGCGTTCCCTCGGCCAGAACCCCTCCGAATCCGAATTGCAAGACATGATCAACGAAGTTGACGCTG

ACAACAACGGCACAATTGATTTCCCTGGTACGAT-CATCACGCCGCAACCTGCTTTCA--------------------------ATGGA

---AA-AACTGACCGC--C----G-CAGAATTCTTGACCATGATGGCCCGCAAAATGAAGGATACCGACTCCGAGGAAGA

GATCCGCGAGGCTTTCAAGGTGTTTGACCGCGACAACAACG

>KJ885266_Talaromyces_cnidii KACC 46617

TG-ACTTTCTAC-TTAT--C--------GCAATGTTG---TGG-T-G--GTGGTTAGCTGACTAG-CCGGTTTGATG-AATAGGACA

AGGATGGAGATGGTGAG---TCGCCACGAACACAAA-C-AACAAC---------CAAA-CGACTTTGAAC-AAAG------GCTAT

CA----------ACGG-----ACAG-TTATTG------ACTCTATC-GAA-----------------TAGGTCAAATCACAACCAAGGAACTGGGC

ACCGTCATGCGTTCCCTCGGCCAGAACCCCTCCGAATCCGAATTGCAGGACATGATCAACGAGGTCGACGCTGA

CAACAACGGCACAATCGATTTCCCTGGTATGACAAAGCGCACTAC-CACT---CGCAATATCAC--------------------ATGG

C---AG-TACTAACTGC--C----G-CAGAATTCCTGACAATGATGGCCCGCAAAATGAAGGATACCGACTCCGAGGAAG

AGATCCGCGAGGCCTTCAAGGTGTTTGACCGTGACAACAATG

>KF741959_Talaromyces_derxii CBS 412.89

TG------CAGG-CTGT--C--------GCATTTTT----TTG-TGGTTGAGGTCA-CTAACCGG--TCTTTTAATG-AACAGGACAAG

GATGGAGATGGTGAGTGACCGA------TACGGCAT-GCCGAC---------GAATATTGTCTCGAATAAAAG------CTTGAATG

--------TACGG-----GCAG-ATACTG------ATAGACTC-GAA-----------------CAGGTCAGATCACTACCAAGGAACTGGGCA

CCGTCATGCGTTCCCTCGGCCAGAACCCCTCCGAATCCGAGTTGCAGGACATGATCAACGAAGTCGACGCTGAC

AACAACGGCACAATTGATTTCCCTGGTACGATTCATCACTCTAGATGGT---GACA--------------------------TCGACGATA

AAAACTGACAGC--C----A-CAGAATTCCTGACCATGATGGCCCGCAAAATGAAGGATACCGACTCCGAGGAAGAGA

TCCGCGAGGCTTTCAAGGTGTTTGACCGCGACAACAACG

>KF741955_Talaromyces_duclauxii CBS 322.48

TG-GATTCCGGG-TTGT--C--------GCAATGTTG---TGG-TGG--GTGGTTAGCTGACTAG-CCGTTTTGATG-AATAGGA

CAAAGACGGAGATGGTGAGTGA--CGC-----CACGAA-C-ACCAAC---------GATATACTCTTGGAAC-AAAG------GCTAT

TGC--------TGCGA-----ACAA-ATATTG------ATTATGCC--AA-----------------CAGGTCAAATCACAACCAAGGAACTGGG

CACCGTCATGCGTTCCCTCGGCCAGAACCCCTCCGAATCCGAATTGCAGGACATGATCAACGAGGTCGATGCTG

ACAACAACGGCACAATCGATTTCCCTGGTATGATACAGCTTC-----TACTTACCGCAACTGTTTCCGATCGCA----------

-AGCGC---AGATACTGACCGT--C----G-TAGAATTCTTGACAATGATGGCTCGCAAAATGAAGGATACCGACTCTGAG

GAAGAGATCCGCGAGGCTTTCAAGGTGTTTGACCGCGACAACAATG

>KJ885271_Talaromyces_euchlorocarpius PF 1203

TGAGATACCCGG-TTGTCGC--------GCAATGTTATGTTGG-TGG--GTGGTTATCTAACTAG-CCGTTTTGATG-AATAG

GACAAGGATGGAGATGGTGAGTGATCCGC-----CACGAA-C-ACCAGC---------GACATGGTCTTTAGAACGAGG------

CTATACAGAATAGATACACGG-----ACAGCATGTTG------ATTATATC-GAA-----------------TAGGTCAAATCACAACCAAG

GAACTGGGCACCGTCATGCGTTCCCTCGGCCAGAACCCCTCCGAATCCGAATTGCAGGACATGATCAACGAGGT

CGACGCTGACAACAACGGCACAATCGATTTCCCTGGTATGATACCAGCACGTCTGCGCTGAATAGATACG-----------

------------GTGGC---GG-GACTAACTGC--C----TATAGAATTCTTGACAATGATGGCCCGCAAAATGAAGGATACCGACT

CCGAGGAAGAGATCCGCGAGGCCTTCAAGGTGTTTGACCGCGACAACAATG

>KF741933_Talaromyces_flavovirens CBS 102801

TG-GCTATCTAC-TTAT--C--------GCAATGTTG---TGG--GA--GTGATTAGCTGACTAG-CCCCTTTGATG-AGTAGGACA

AGGACGGAGATGGTGAG---TCGCCACGAACACGGA-C-AACAAC---------CAAA-AGATTCTCAAT-AAAG------GCTAT

CG----------ACGA-----ATAA-ATGTTG------ACTTTATT-GAA-----------------TAGGTCAAATCACAACCAAGGAACTGGGC

ACCGTCATGCGTTCTCTCGGCCAGAACCCCTCCGAATCCGAATTGCAGGACATGATCAACGAAGTCGACGCTGA

CAACAACGGCACAATCGATTTTCCTGGTATGACAAAGCACACTAT-CACG---T-CAATATTCC--------------------ATGGC-

--AG-TACTAACTGC--C----G-CAGAATTCCTGACAATGATGGCCCGCAAAATGAAGGATACCGACTCCGAGGAAGAG

ATCCGCGAGGCTTTCAAGGTGTTTGACCGCGACAACAATG

>KF741949_Talaromyces_flavus CBS 310.38

TG-GATTTCCGG-TTGT--C--------GCAATGTGG---TGG-TGG--GTGGTTAGCTGACTAG-CCGTTTGGATG-AGTAGGA

CAAGGATGGAGATGGTGAGTGAAC--------CACGAA-C-ACCAAC---------GATA-CAGCAACGAAT-AAGTACTGTCGTT

ATGAT--------TGCGA-----GCAAATTATTG------ACGGGGACTGAA-----------------CAGGTCAAATCACAACCAAGGAACT

GGGCACCGTCATGCGTTCCCTCGGCCAGAACCCCTCCGAATCCGAATTGCAGGACATGATCAACGAAGTCGACG

CTGACAACAACGGCACAATCGATTTCCCTGGTATGGCCAAAC----------CTGTCTACGGTTATTACA------------------AT

GGC---AG-AACTAACTGC--C----AACAGAATTCTTGACAATGATGGCCCGCAAAATGAAGGATACCGATTCCGAGGA

AGAGATCCGCGAGGCTTTCAAGGTGTTTGACCGTGATAACAATG

>KF741945_Talaromyces_funiculosus CBS 272.86

TG-GATTCCCGG-TTGT--C--------GCAATGTTA---TG-------GTGGTTAGCTGACTAG-CCGTTGAAATA-ATTAGGACAA

GGATGGAGATGGTGAG---TCCGC-----CACGAA-T-GATAAC---------CAGA-TGATCTTGAGC-AAAG------TTTATCGC--

------ATCGA-----ATAT-ATATTA------ACTATATT-CAA-----------------AAGGCCAAATCACAACCAAGGAACTGGGCACTGT

CATGCGTTCCCTCGGCCAGAACCCCTCCGAATCCGAATTGCAGGACATGATCAATGAAGTCGATGCTGACAACAA

CGGAACAATCGATTTCCCTGGTATGACAAACTGCA-----AACT---CTCAGTTTTCT--------------------ACCAC---AG-TACT

AACTAA--C----A-CAGAATTCTTGACTATGATGGCCCGCAAAATGAAGGATACTGACTCCGAGGAAGAGATCCGTGA

GGCTTTCAAGGTGTTTGACCGTGACAACAATG

>KF741966_Talaromyces_galapagensis CBS 751.74

TG-GATTTCTGG-TTGT--C--------GCATTGTTT---TGG-TGG--CTGGTTAGCTGACTAG--CGTTTTGATG-AGTAGGACA

AGGATGGTGATGGTGAG---TCCGC-----CACGAA-C-A-CGAT---------GATA-TCGTCTCGAAC-AAAG------GTTGTTTC-

--------ACGA-----ACAG-ATATTG------ATAAACTC-CAA-----------------TAGGCCAAATCACAACCAAGGAATTGGGCACT

GTCATGCGTTCCCTCGGTCAGAACCCCTCCGAATCTGAATTGCAGGACATGATCAACGAAGTCGACGCTGACAA

CAACGGCACAATTGATTTCCCTGGTATGACAAACCACA-----AGCT---CGCGATTTTT---------------------ATGGCGATAC

TTACTAACTGC--C----G-CAGAATTCTTGACGATGATGGCCCGTAAAATGAAGGATACCGACTCCGAGGAAGAGATC

CGTGAGGCATTCAAGGTGTTTGACCGTGACAACAATG

>KF741931_Talaromyces_indigoticus CBS 100534

TG-GATTCCTGG-TTGT--C--------GCAATGTTG---TGG-TGG--GTGGTTAGCTGACTAG--CGGTTTGATG-AGTAGGAC

AAGGATGGTGATGGTGAG---TTCGC-----CACGAA-C-AACGAC---------CAAA-AGACCTTGAAC-AAAG------GTCATT

GC--------TACGA-----ACAG-ATATTG------ACTATATC-GAA-----------------TAGGTCAAATTACGACCAAGGAATTGGGCA

CCGTCATGCGTTCCCTCGGCCAGAACCCCTCCGAATCCGAATTGCAGGACATGATCAACGAAGTCGACGCTGAC

AACAACGGCACAATTGATTTCCCTGGTATGACGAACCACACCAC-CACT---CGCAATATTAC--------------------ATAGC-

--AG-TACTAACTGC--T----A-CAGAATTCTTGACAATGATGGCTCGCAAAATGAAGGATACCGACTCCGAGGAAGAGA

TCCGCGAGGCTTTCAAGGTGTTTGACCGTGACAACAATG

>KJ885290_Talaromyces_intermedius CBS 152.65

TG-GATTCCTAG-TTGT--C--------GCAATGTTATGCTGG-TGG--GTGGCTAGCTGATTAG-CCGTTTTGATG-AGTAGG

ACAAGGATGGAGATGGTGAGTGATCCGC-----CTCGAC-C-ACCGAC---------GAGATAGTCTTCGAAC-AAAG------GC

TTTTGT--------TCCGATGTGGACAGCATATTG------ACTATATCGGAA-----------------TAGGTCAAATTACAACCAAGGAG

CTCGGCACCGTCATGCGTTCCCTCGGCCAGAACCCCTCCGAATCCGAATTGCAGGACATGATCAACGAAGTCGA

CGCTGACAACAACGGCACAATTGATTTCCCTGGTATGACACACTACA------ACT---CGCAATCGTCT--------------------A

TGGC---AGTTGCTAACTGCTAC----A-CAGAATTCTTAACAATGATGGCCCGCAAAATGAAGGATACCGACTCCGAG

GAAGAGATCCGGGAGGCTTTCAAGGTGTTTGACCGTGACAACAATG

>KJ885257_Talaromyces_liani CBS 225.66

TA-GCAATCTGG-TTGT--C--------GCAATGTTG---TGG-TGG--GTGGTTAGCTGACTAG-CCGTTTTGATG-AGTAGGA

CAAGGATGGAGATGGTGAG---TCGGC-----CACGAA-C-AATATC---------CAAA-AGGCGTTGAAC-AAGG------GTTAT

CGC--------CGCGA-----ACAG-ATATTG------ACTATATC-GAA-----------------TAGGTCAAATCACAACCAAGGAACTGGG

CACTGTCATGCGTTCCCTCGGCCAGAACCCCTCCGAATCCGAACTGCAGGACATGATCAACGAAGTCGACGCTG

ACAACAACGGCACAATCGATTTCCCTGGTATGATATAAT--------TGCT---CACAGGATATTACG-----------------ATGGC---

AG-TACTAACTGC--C----G-CAGAATTCCTGACAATGATGGCCCGCAAAATGAAGGACACCGACTCCGAGGAAGAG

ATCCGCGAGGCATTCAAGGTGTTTGACCGTGACAACAATG

>KF741952_Talaromyces_macrosporus CBS 317.63

TG-GATTCCTGG-TTGT--C--------GCAATGT-----------G--GTGATTCGCTGACTAG-CCG-TTTGATG-CGCAGGACAAG

GATGGAGATGGTGAG---TCCGC-----CACGAA-C-AATAGC---------CAAA-AGGCCTCGAAC-AAAG------GCCGTCGC--

------TGCGA-----ACAG-ATATTG------ACTATATC-GAA-----------------TAGGCCAAATCACAACCAAGGAACTGGGCACC
GTCATGCGTTCCCTCGGCCAGAACCCCTCCGAATCCGAACTGCAGGACATGATCAACGAGGTCGATGCTGACAA
CAACGGCACAATCGACTTCCCTGGTATGACAAACTACA-----AACT---CGCATTCT-----------------------ATGGC---AG-TA
CTAACTGC--C----G-CAGAATTCTTGACAATGATGGCCCGCAAGATGAAGGATACCGACTCCGAGGAAGAGATCCG
CGAGGCTTTCAAGGTGTTTGACCGTGACAACAATG

>KF741958_Talaromyces_marneffei CBS 388.87

TG-GATTTCTGG-TTGT--C--------GCAATGTTG---TGG-TGG--GTGGTTCGCTGACTAG-CCGTTTGGATG-AATAGGA
CAAGGATGGTGATGGTGAGTGA--CGC-----CACGAA-C-ACCAGA---------CATATAGTCTTCGAACAAAAA------GTTAT
TAC--------TGCGA-----ACAG-ATATTA------ATAACATC--AA-----------------TAGGTCAAATTACAACCAAGGAACTGGGCA
CCGTCATGCGTTCCCTCGGCCAGAACCCTTCCGAATCCGAATTGCAGGACATGATCAACGAGGTCGACGCTGAC
AACAACGGCACAATCGATTTCCCTGGTATGATGCAGCCTC-----TATTTATCGCAGCCGTTTCCGATCATA-----------AG
GGC---AGATACTGACTGC--C----T-TAGAATTCTTGACAATGATGGCCCGCAAAATGAAGGATACCGACTCCGAGGA
AGAGATCCGCGAGGCTTTCAAGGTGTTTGATCGTGACAACAATG

>KJ885274_Talaromyces_muroii CBS 756.96

TG-GATTTCTAC-TTGT--C--------GCAATGTTG---TGG-TGG--GTGGTTAGCTGACTAG-CCGTTTTGATG-AGTAGGAC
AAGGATGGAGATGGTGAG---TCCGC-----CACGAA-A-AATAAC---------CAAA-AGGCCTTGAAC-AAAG------GTTATCG
C--------TGCGA-----ACAG-ATATTG------ACTATGTC-GAA-----------------TAGGTCAAATCACAACCAAGGAACTGGGCA
CCGTCATGCGTTCTCTCGGCCAGAACCCCTCCGAATCCGAATTGCAGGACATGATCAACGAAGTCGACGCTGAC
AACAACGGCACAATCGATTTCCCTGGTATGACAAACCACA-----AGCT---CGCAATAATCT--------------------ATGGC---A
G-TACTAACTGC--C----G-CAGAATTCTTGACAATGATGGCCCGCAAAATGAAGGATACCGACTCCGAAGAAGAGAT
CCGCGAGGCTTTCAAGGTGTTTGACCGCGACAACAATG

>KJ775425_Talaromyces_oumae-annae CBS 138208

TG-GATTTCTGA-TTGA--C--------GCAATGTTT-----G-TGATCGTGGTTAGCTGACTAG-CCGTTTTGATG-AGTAGGAC
AAGGATGGAGATGGTGAGTG-ACCGC-----CACGAA-C-GTCGAT---------GATACAGTCAACGAAT-AAGGGCTATCCT
TATGAT--------TGCGA-----ACAGCATATTG------ATGGTATTTGAA-----------------CAGGTCAGATCACCACCAAGGAATT
GGGCACCGTCATGCGTTCCCTCGGCCAGAACCCCTCCGAATCCGAATTGCAGGACATGATCAACGAGGTCGACG
CTGACAACAACGGCACAATCGATTTCCCTGGTATGACATAACC-------TATCAGACGCAGCCATATATGACTATCGA---
------GTGGC---AG-AACTAATTGC--C----A-CAGAATTCTTGACAATGATGGCCCGCAAAATGAAGGATACCGACTCCG
AGGAAGAGATCCGTGAGGCTTTCAAGGTGTTTGACCGCGACAACAATG

>KF741936_Talaromyces_panamensis CBS 128.89

TG-GATTTTTAGTTGGT--C--------GCAATGTTG---TGG-TGG--GCTGTTAGCTGACGCG-CCGTGTTGATG-AGTAGGA
CAAGGATGGCGATGGTGAGTGATCCGC-----CACGAG-C-ACCAAC---------AATG-CAGTTTTGAGC-AAGG------GATA
TGTATGATGAAACATGA-----ACAACGGATTG------ATTGTATCTAAA-----------------TAGGTCAAATCACAACCAAGGAA
CTCGGCACCGTCATGCGTTCCCTCGGCCAGAACCCCTCCGAATCCGAATTGCAGGACATGATCAACGAGGTCGA
CGCCGACAACAACGGCACAATCGATTTCCCTGGTATGAGCCAACCCGTCTA-CGTTTGTTACG--------------------------
-GGGGC---AG-AACTAACTGC--C----TACAGAATTCTTGACAATGATGGCTCGCAAAATGAAGGATACCGACTCCGAA
GAAGAGATCCGTGAGGCTTTCAAGGTGTTTGACCGCGACAACAATG

>KF741964_Talaromyces_pinophilus CBS 631.66

TG-GAAATCTGG-TTGT--C--------GCAATGTTG---TGG-TGG--ATGGTTAGCTGACTAG-CCGTTTTGATG-AGTAGGAC
AAGGATGGAGATGGTGAG---TCCGC-----CACGAA-C-A-CGAC---------GATATTTGTCTCGAAC-AAAG------GTTTTTT
C---------ACGA-----GCAT-ATATTG------ATAAAATC-TAA-----------------TAGGCCAAATTACAACTAAGGAACTGGGCACC
GTTATGCGTTCCCTCGGCCAGAACCCCTCCGAATCCGAACTGCAGGACATGATCAACGAAGTCGACGCTGACAA
CAACGGCACAATCGATTTCCCTGGTATGATATAAT--------TGTT---CACGGGTTTATACG-----------------ATGGC---AG-T
ACTAACTGC--C----G-CAGAATTCTTGACAATGATGGCCCGCAAAATGAAGGATACCGACTCCGAGGAAGAGATCC

GTGAAGCTTTCAAGGTGTTTGACCGTGACAACAATG

>KF741954_Talaromyces_primulinus CBS 321.48

TT-CATTTCTGG-TTGT--C--------GCAATGTTG---TGG-TGG--GTGGTTAGCTGACCAG-CCGTTTTGATG-AGTAGGAC

AAGGATGGAGATGGTGAGTG-ACCAC-------CGAA-C-ACCAAC---------GTTACAGTCAACGAAT-AAGGGCTATCTTTA

TGAT--------TGGGA-----ACAG-GTATTA------ATCGTC---GAA----------------TAGGTCAAATCACAACCAAGGAACTGGG

CACCGTCATGCGTTCCCTCGGCCAGAACCCCTCCGAATCCGAGTTGCAGGACATGATCAACGAAGTCGACGCTG

ACAACAACGGCACAATCGATTTCCCTGGTATGACTCATCACGTCTATTGCTTGATACG--------------------------ATGGC

---AG-TACTGACCGC--T----G-CAGAATTTTTGACAATGATGGCCCGCAAAATGAAGGATACCGACTCCGAGGAAGA

GATCCGCGAGGCTTTCAAGGTGTTTGACCGCGACAACAATG

>KF741947_Talaromyces_purpurogenus CBS 286.36

TGCAATCATTGT-TTG------------GGTATGTTG---T---TGG--CCGGTTATCTAATTAGCCCGTTTGGATG-AGTAGGACAA

GGATGGTGATGGTGAG---TTCACCCGGACACGCAGCGATCAAC---------GATA-AGACTCTGA----------------------------

----------ACAGGATATTT------ACTATATC-GAT----------------TAGGTCAAATCACAACCAAGGAACTGGGCACCGTCATG

CGCTCCCTCGGCCAGAACCCCTCCGAATCCGAATTGCAGGACATGATCAACGAAGTTGACGCTGACAACAACGG

CACAATCGATTTCCCTGGTATGATGA-----------CTCTCGCTACAATCTACT-------------------GCGGATA-GGTAACTGAT

TGA--TAATGGATAGAATTCTTGACAATGATGGCCCGCAAAATGAAGGATACCGACTCCGAGGAAGAGATCCGTGA

GGCTTTCAAGGTGTTTGACCGTGACAACAATG

>KF741938_Talaromyces_ruber CBS 132704

TG-GAAACTTGGTTTGT--C--------ACAATGTTG--------GC--GTGGTTAGCTGACTAG-CCGTTTTGATG-AATAGGACA

AGGATGGAGATGGTGAG----TCGC-----CGCGAA-C-AATGAA-------------ACACCTTGAAC-GAAC------ATTACCGC----

----AGTCA-----ACAG-ACATTG------ACCCTATC-GGA----------------CAGGACAAATCACAACCAAGGAACTCGGCACA

GTCATGCGTTCTCTCGGCCAGAACCCCTCCGAATCCGAATTGCAGGACATGATCAACGAAGTCGACGCTGACAA

CAACGGCACAATTGATTTCCCTGGTATGACTGACCACC-----AACT---CGCAATATTTG-------------------ATGGC---AG-

TACTGACTGC--C----G-CAGAATTCTTGACAATGATGGCCCGCAAAATGAAGGATACCGACTCCGAGGAAGAGATC

CGTGAGGCTTTCAAGGTGTTTGACCGTGACAACAATG

>KF741956_Talaromyces_rubicundus CBS 342.59

TG-GATTTCTTC-TTGT--C--------GCAATGTGG-------TGG--GTGGTTAGCTGACTAG--CGGTTTAATG-AGTAGGACAA

GGATGGAGATGGTGAG---TTCGC-----CATGAA-C-AACGAA---------CAAA-AGACCTTGCTC-AAAG------GTCATTGC-

-------TGCGA-----ACAG-ATATTG------ACTATATC-GAA----------------TAGGTCAAATCACAACCAAGGAATTGGGCACT

GTCATGCGTTCCCTCGGCCAGAATCCCTCCGAATCCGAATTGCAGGACATGATCAACGAAGTCGACGCTGACAA

CAACGGCACAATCGATTTCCCTGGTATGACGAACCACACTAC-CACT---CGCAATATTCC-------------------ATAGC---A

G-TACTAACTGC--T----A-CAGAATTCTTGACAATGATGGCCCGCAAAATGAAGGATACCGACTCCGAAGAAGAGATC

CGTGAGGCTTTCAAGGTGTTCGATCGTGACAACAATG

>KJ775422_Talaromyces_sayulitensis CBS 138204

TG-CAAATCTGG-TTCT--C--------GCAATGTTG---TGG-TGG--GTGGTTAGCTGACTAG-CCGTTTTGATG-AGTAGGA

CAAGGATGGAGATGGTGAG---CCCGC-----CACGAA-C-AATAAC---------CAAA-AAGCCTTGAAC-AAAG------GTTGG

TTC---------GCGA-----ACAA-ATATTG------ATATGCTT-GAA----------------TAGGCCAGATCACAACCAAGGAACTGGGC

ACCGTCATGCGTTCTCTCGGCCAGAACCCCTCCGAATCCGAACTGCAGGACATGATCAATGAAGTGGACGCTGA

CAACAACGGCACAATCGATTTTCCTGGTATGATATAAT--------TGCT---CACGGATTATTACG----------------ATGGC---A

G-TACTAATTGC--C----G-CAGAATTCTTGACAATGATGGCACGCAAAATGAAGGATACCGACTCGGAAGAAGAGAT

CCGTGAGGCTTTCAAGGTGTTTGACCGTGACAACAATG

>KF741960_Talaromyces_siamensis CBS 475.88

TG-ACTTTCTAC-TTAT--C--------GCAATGTTG---TGG-T-G--GTGGTTAGCTGACTAG-CCGGTTTGATG-AATAGGACA

AGGATGGAGATGGTGAG---TCGCCACGAACACAAA-C-AACAAC---------CAAA-CGACTTTGAAC-AAAG------GCTAT

CA----------ACGG-----ACAG-TTATTG------ACTCTATC-GAA-----------------TAGGTCAAATCACAACCAAGGAACTGGGC
ACCGTCATGCGTTCCCTCGGCCAGAACCCCTCGGAATCCGAATTGCAGGACATGATCAACGAGGTCGACGCTGA
CAACAACGGCACAATCGATTTTCCTGGTATGACAAAGCACACAAC-TACT---CGCAATATTCC--------------------ATGG
C---AG-TACTAACTGC--T----G-CAGAATTCCTGACAATGATGGCCCGCAAAATGAAGGATACCGACTCCGAGGAAGA
GATCCGCGAGGCCTTCAAGGTGTTTGATCGTGACAACAATG

>KF741957_Talaromyces_stipitatus CBS 375.48

TGCAGTGATTGT-TTG------------GTAATGGT-------------GTGGTTATCTAACTAA-CCGTTTCGATC-AATAGGACAAGGA
TGGCGATGGTGAG---TTCACCCAAACACGCAGC-ACCAAC---------GATAGGGTATATTAGT-----------ATGATC----------
TACGG-----ACGG-ACATTG------ACAATATT-GAT-----------------CAGGCCAAATTACCACCAAGGAACTGGGCACCGTC
ATGCGTTCGCTCGGCCAGAACCCCTCCGAATCCGAATTGCAGGACATGATCAACGAAGTTGACGCTGACAACAA
CGGCACAATCGATTTCCCTGGTATGACGACTCTAC-----TACCCCTTACAATCCAGT--------------------GTGGATA-GGT
GACTTACTGA--TGCTGGCTAGAATTTTTGACAATGATGGCCCGCAAAATGAAGGATACCGACTCCGAGGAAGAGA
TCCGTGAGGCTTTCAAGGTCTTTGATCGCGACAACAATG

>JX315646_Talaromyces_stollii CBS 408.93

TG-GATTCCTTG-TTGT--C--------GCAATGCTA---GCG-TGG--GTGGTTTGCTGACGAG-CCGTCTTGATG-AGTAGGA
CAAGGATGGAGATGGTGAG----TCGC-----CGCGAA-C-AATGAA--------------GCTGCTTGAAC-GAAT-------------C-------
-AGCCA-----ACAG-ACCTTA------ACTCTATC-GAA-----------------CAGGACAAATCACAACCAAGGAACTTGGCACCGT
CATGCGTTCCCTCGGCCAGAACCCCTCCGAATCCGAATTGCAGGACATGATCAACGAAGTCGACGCTGATAACAA
CGGCACAATCGATTTCCCTGGTATGATAGACCACC-----AGCT---CGCACTTTTTG--------------------ATGGC---AG-CAC
TAACTGC--C----G-CAGAATTTTTGACAATGATGGCCCGCAAAATGAAGGATACCGACTCCGAGGAAGAGATCCGT
GAGGCTTTCAAGGTGTTTGACCGTGATAACAATG

>KF741940_Talaromyces_thailandensis CBS 133147

TG-GGTTTCCGG-TTGT--C--------GCAATGTTG---TGGCTGG--GTGGTTAGCTGACTAG-CCGTTTTGATG-AATAGGA
CAAGGATGGAGATGGTGAGTGAGCCGC-----AATGAT-C-ACCAAC---------GATGCAATCTTTGGACAAGAA--------TAT
CAG--------CACGA-----ACAGTATCCTG------ACTATATCTGAA-----------------TAGGTCAAATCACAACCAAGGAACTGG
GCACCGTCATGCGTTCCCTCGGCCAGAACCCCTCCGAATCCGAGCTGCAGGACATGATCAACGAAGTCGACGCT
GACAACAACGGCACAATCGATTTCCCTGGTATGAAACAAT--------TGCT---TACGGGCGTTACG------------------ATGG
C---AGAAACTAACTGC--C----TACAGAATTCTTGACAATGATGGCCCGCAAAATGAAGGATACCGACTCCGAGGAAG
AGATCCGTGAGGCTTTCAAGGTGTTTGATCGCGACAACAATG

>KF741944_Talaromyces_verruculosus CBS 388.48

TG-AATTTCTGG-TTAT--C--------GCAATGTTG---TGG-TGG--GTAGTTAGCTGACTAG-CCGTTTTGTTG-AGTAGGAC
AAGGATGGAGATGGTGAGTG-ACCGC-----CACCAA-C-ACCGAC---------GATC-TAGTCTCGAAC-AAAG------GCTAT
TTG--------TACGA-----TCAG-ATATTG------ACGATATCTGAA-----------------TAGGTCAAATCACAACCAAGGAACTGGG
CACTGTCATGCGTTCCCTCGGCCAGAACCCCTCCGAATCCGAATTGCAGGACATGATCAACGAGGTTGACGCTG
ACAACAACGGCACAATCGATTTCCCTGGTATGACATAACC-------TATCAACCGCAAACATATATGACAGTTGG---------
ATGGC---AG-AACTAACTGC--C----A-CAGAATTCTTGACAATGATGGCTCGCAAAATGAAGGATACCGACTCCGAGG
AAGAGATCCGTGAGGCTTTCAAGGTGTTTGACCGCGACAACAATG

>KF741935_Talaromyces_viridis CBS 114.72

TG-TATTTCTTG-TTGT--C--------GCAATGTTG---TGG-TGG--GTGGTTAGCTGACTAG-TCGTTTTGATG-AGTAGGAC
AAGGATGGAGATGGTGAGTCAACCGC-----CACG-------CAAC---------GATACAATCTTTGAAC-AAAATCTACTATTGT
AAT--------TACGA-----ACAAAATATTG------ACCATATCTGAA-----------------AAGGTCAAATCACAACCAAGGAACTGGG
CACCGTCATGCGTTCCCTCGGCCAGAACCCCTCCGAATCCGAATTGCAGGACATGATCAACGAAGTCGACGCTG
ACAACAACGGCACAATCGATTTCCCTGGTATGACAGA-CACACTAC-AACG---CACAATCGTCT--------------------ATG
GC---AGAAACTAACTGC--C----GATAGAATTCTTGWCAATGATGGCCCGCAAAATGAAGGATACCGACTCCGAGGA

AGAGATCCGTGAGGCTTTCAAGGTGTTTGACCGTGACAACAATG

>KF741943_Talaromyces_viridulus CBS 252.87

TG-GATTTCTGG-TTGT--C--------GCAATGTTG---TG--TGG--GTAGTTAGCTGACGAG-CCGTTTTGATG-AGTAGGAC

AAGGATGGAGATGGTGAGTG-ACCAC-----CACAAA-C-ACCGGC---------GATA-TGGTCTTGAAC-CGAG------GTTATT

AC--------TACGA-----ACAT-ATATTG------ACGGTATTTGAA-----------------CAGGTCAAATCACAACCAAGGAACTGGGC

ACCGTCATGCGTTCCCTCGGCCAGAACCCCTCTGAATCCGAATTGCAGGACATGATCAACGAGGTCGACGCTGA

CAACAACGGCACAATCGATTTCCCTGGTATGACCCAACACGTCTA-CGCTGAACACG---------------------------ATGGC-

--GG-TACTAACTGC--C----A-CAGAATTCTTGACAATGATGGCCCGCAAAATGAAGGATACCGACTCCGAGGAAGAG

ATCCGCGAGGCTTTCAAGGTGTTTGACCGCGACAACAATG

>KP765382_Talaromyces_qii_AS3.15414

TG-GGTTTCCGG-TTGT--C--------GCAATGTTG---TGG-CGG--GTGGTTAGCTGACTCG-CCGTTTTGATG-AGTAGGA

CAAGGATGGAGATGGTGAGTGAGCCGC-----AATGAT-C-ACCAAC---------GATGCAGTCTTTGGACGAGTA--------AA

TCAG--------CACGA-----ACAGTATCCTG------ACTATATCTGAA-----------------TAGGTCAAATCACAACCAAGGAACTG

GGCACCGTCATGCGTTCCCTCGGCCAGAACCCCTCCGAATCCGAACTGCAGGACATGATCAACGAAGTCGACG

CTGACAACAACGGCACAATCGATTTCCCTGGTATGACACAATC-------ATCT---TACGGTCGCTACG------------------AT

GGC---AGAGACTAACTGC--C----TACAGAATTCCTGACAATGATGGCCCGCAAAATGAAGGATACCGACTCCGAGG

AAGAGATCCGTGAGGCTTTCAAGGTGTTTGACCGCGACAACAATG

>KP765383_Talaromyces_fusisporus_AS3.15415

TG-GATTCCTGG-TTGTCGCGTTGTTGTGCAATGCTA---TAG-CGG--GCGGTTAGCTGACTAG-CCGTTTTGATG-AAT

AGGACAAGGATGGAGATGGTGAGTTTCTTAC--GAACACGAA-C-GACAAC---------GATACGATTTTTGGA--GAAT-----

-ATTATGAC--------TACGA-----ACAACATATTG------ATAAAGTC-CAA-----------------TAGGTCAGATCACAACCAAGGAA

CTGGGCACCGTCATGCGTTCCCTCGGCCAGAACCCCTCCGAATCTGAATTGCAGGACATGATCAACGAAGTCGA

CGCTGACAACAACGGCACAATCGATTTCCCTGGTATGACAGTACACA-----AAAT---TGCAATTTTTGG-------------------

AGGGC---AG-TACTAACTGC--C----G-CAGAATTCTTGACAATGATGGCCCGCAAAATGAAGGATACCGACTCCGAG

GAAGAAATCCGTGAGGCTTTCAAGGTGTTTGACCGTGACAACAATG

>KJ885289 Talaromyces_helicus CBS_335.48

TG------ACGGTTTGA-----------GCAAGAATT---GTG-TGGCAGCAATCTAATATTTTGATCTTGATGATG-TACAGGACAA

AGATGGCGATGGTGAG---TTTGT-----CGAGAG-C-ATTTACTCCGAATTGCAGATAAGCAATCAAC-GAAGAAAAAAG

GGGTCGA--------CATGG-----ACACAAACCTGTGGAATACTGTACTAAAAGCGTGATCGACGTTGGCCAGGTCAAAT

CACAACCAAAGAACTCGGTACCGTCATGCGCTCTCTCGGCCAGAACCCTTCCGAATCCGAATTGCAGGACATGAT

CAACGAAGTCGACGCTGATAACAACGGCACAATTGACTTTCCTGGTATGATCAAAATCCGTCCTCACT---CCCGCT

GTTTTGCCAGCTCCCCCCTCGCAGTGTGAT---TAATAGAAAGGGT-------ATTAGAATTCTTGACCATGATGGCTCGC

AAAATGAAGGATACCGACTCCGAAGAAGAAATCCGCGAGGCATTCAAGGTGTTTGACCGCGACAACAATG

Data S1. The sequence alignment of *CaM*.

Supplementary dataset 2

>KF741929_Talaromyces_aculeatus_ CBS 289.48 bt

GTGAGTTTG-ACTCT-CGAC--------CCAAGCTTTCTA-TCAATTGTCGCGAC-AACACGCT------GACTCT-TTCCAGGC

AAATCATCTCTGCTGAGCATGGTCTCGATGGCTCTGGTGTGTAA---------GTATC---CATGA-ATGG-AATCCAGCTAC

AATCCGACA--A-TATCTGATAATC-AACAGCTACAATGGCTCCTCTGACCTCCAGTTGGAGCGTATGAACGTCTACT

TCAACGAGGTGCGT------------CG----------------AAC-----CAAT-CCACCAAAT-AACGA--A-ACAA------------AC---TCACA

A-TG--G--TA-TAGGCCTCCGGCAACAAATACGTCCCCCGTGCTGTCCTCGTCGACTTGGAGCCCGG

>JX315623_Talaromyces_amestolkiae CBS 132696

GTGAGTTTG-A--CT-CGTC--------CCAAGC-TACTA-TCAATTGTCGCGAC-AGCACGCT------GACT-T-ATCCAGGCAA

ATCATCTCTGCTGAGCACGGTCTCGATGGCTCTGGTGTGTAA---------GTATT--TCACAG-TTCG-AATACACCTACAG

TCCGACA--A-CATCTGATCATC-GACAGCTACAATGGCTCCTCCGACCTCCAGTTGGAGCGTATGAACGTCTACTT

CAACGAGGTGCGT------------TA---------------GAAAG----TCTC-TCGACTCCT--ATAG--A-ACAG------------AC---ACTCAT-

TC--A--TC-TAGGCCTCCGGCAACAAATACGTCCCCCGTGCCGTCCTCGTCGATTTGGAGCCCGG

>KF183640_Talaromyces_angelicus KACC 46611

GTGAGTTTT-A--CT-CGAC--------CCGAACTTTCTA-TCAATTGTCGCGAC-AACACGCT------GACT-T-TTCCAGGCAA

ATCATCTCTGCTGAGCACGGTCTCGATGGCTCTGGTGTGTAA---------GTATT--ACATGA-TTCG-ATTCCAGCTACAA

TCCAACA--A-TATCTGATCATC-AACAGCTACAATGGCTCCTCCGACCTCCAGTTGGAGCGTATGAACGTCTACTTC

AACGAGGTGCGT------------CA----------------GAC-----GATT-CCACCATATAAACAA--A-ACAAA-----------AC---TCATCG--

G--G--TA-TAGGCCTCCGGCAACAAATACGTTCCCCGTGCTGTCCTCGTCGACTTGGAGCCCGG

>KF741916_Talaromyces_apiculatus CBS 312.59

GTGAGTTTG-ACACT-CGACA-------CCAAGCTTTCTA-TCAGTTGTCGCGTC-AACACGCT------GACTCTGTCCCAG

GCAAATCATCTCTGCTGAGCACGGTCTCGATGGCTCCGGTGTGTAA---------GTATC---CACGG-TTCG-AATCCAGG

TACAATTCGACA--A-TATCTAATAATC-AACAGCTACAATGGCTCCTCTGACCTCCAGTTGGAGCGTATGAACGTCTA

CTTCAACGAAGTGCGT------------CG---------------AAAC-----CAAT-CCACCATAT-AACGA--A-ACAA------------AC---TCA

CAG-TG--G--TA-TAGGCCTCCGGCAACAAATACGTCCCCCGTGCTGTCCTGGTCGACTTGGAGCCCGG

>KF741917_Talaromyces_aurantiacu CBS 314.59

GTGTGTAAAAA-----AGAC----------------TTGG-TCAATTGTCGCCACAAAAAAGCT------GAAT-T-TTCTAGGCAAATCAT

CTCTGCTGAGCACGGTCTCGATGGCTCCGGTGTGTAA---------GTGTTACAAACCA-TTCG-AATGCAGTTATAATCC

GACA--C-CATCTGATCATC-AATAGCTACAATGGCTCCTCCGACCTCCAGTTGGAGCGTATGAACGTCTACTTCAAC

GAGGTGCGTGGAAACAACCATCA---------------GAAA-------AT-CAATCGAATGCTTGG--A-----------------AC---TCATCT

CTC--GAATA-TAGGCCTCCGGCAATAAGTACGTTCCCCGTGCCGTCCTTGTCGACTTGGAGCCCGG

>HQ156944_Talaromyces_calidicanius CBS 112002

GTGAGTTTG-ACCTT-CACC--------CCAAATTTTCAACTCAATTATCGCGAC-AACACGCTGACTCGGACTCT-TCCCA

GGCAAATTATCTCCGCTGAGCACGGTCTCGACGGCTCCGGTGTGTAA---------GTATT--ACACTA-TTCA-AATCCAG

CTACAATCCGACA--AATATCTGATAATC-AACAGCTACAATGGCTCCTCCGACCTCCAGTTGGAGCGTATGAACGTT

TACTTCAACGAGGTTCGT------------CA----------------GAC-----CCAT-CCACCTCATAAACCGCAA-ACAA------------GC---

TCATGATCG--A--TA-CAGGCTTCCGGCAACAAATATGTCCCCCGTGCTGTCCTCGTCGACTTGGAGCCCGG

>KF183641_Talaromyces_cnidii KACC 46617

GTGAGTTTG-A--CT-CGAC--------CCAAACTTTCTA-TCAATTGTCGCGAC-AACACGCT------GACT-T-TTTCAGGCAA

ATCATCTCTGCTGAGCACGGTCTTGATGGCTCTGGTGTGTAA---------GTATT---CACGAATTCG-AATCCAGCTACAA

TCCGACA--A-TATCTGATCGTC-AACAGCTACAATGGCTCCTCCGACCTCCAGTTGGAGCGTATGAACGTCTACTTC

AACGAGGTGCGT------------CG----------------AAC-----CAAT-CCACCATAT-AACGA--A-ACAA------------AC---TCACAA-T

G--G--TATTAGGCCTCCGGCAACAAATACGTCCCCCGTGCTGTCCTCGTCGACTTGGAGCCCGG

>JX494306_Talaromyces_derxii CBS 412.89
GTGAGTTCTGACT---CGACTG------TAAAAGTCTCAG-TCAATTGTCGCGACAAACATGCT------GACT-T-TACCAGGC
AAATCATCTCTGCCGAGCACGGTCTCGACGGCTCTGGTGTGTAA---------GTGTTGCAAACGA-CTCG-GATCCAAA
TACAAGCCGATAGTA-TATCTGATTATC-AACAGCTACAATGGCTCCTCCGACCTCCAGTTGGAGCGTATGAACGTC
TACTTCAATGAGGTGCGTGAAATTAACCACCG---------------AAAA-----AAAC-CCATCGAATGCTTGA--------------------A
C---TGATGTTTC--GAATA-TAGGCCTCCGGCAACAAATACGTGCCCCGTGCTGTCCTCGTCGACTTGGAGCCCGG
>JX091384_Talaromyces_duclauxii CBS 322.48
GTGAGTTTG-ACCTT-CACT--------CCGAATTTTCAAGTCAATTGTCGCGAT-AACACGCTGACTCTGACTCT-TTCCA
GGCAAATTATCTCTGCTGAGCACGGTCTCGACGGCTCCGGTGTGTAA---------GTATT--ACTCAA-TTCA-AATCCAG
CTACAATCCGACA--AGTATCTGATAATC-AACAGCTACAATGGCTCCTCCGACCTCCAGTTGGAGCGTATGAACGTT
TACTTCAACGAGGTTCGT------------CA----------------GAC-----CCAT-CCACACCATAAACCGCAA-ACAA------------GC---
TCATGATCG--A--TA-CAGGCTTCCGGCAACAAATATGTCCCCCGTGCTGTCCTCGTCGACTTGGAGCCCGG
>KJ865733_Talaromyces_euchlorocarpius PF 1203
GTGAGTTGG-ACTTT-CGACAA------AAAAACTTTCAG-TCAATTGTCGCGAC-ACCACGCT------GACTCT-TTGCAGG
CAAATCATCTCCGCTGAGCACGGTCTCGACGGCTCTGGTGTGTAA---------GTGTT-GGCAAAA-TTCGAAATCCAG
CTACAATCCGACA--A-TATCTGATAATC-ACCAGCTACAATGGCTCCTCCGACCTCCAGTTGGAGCGTATGAACGTC
TACTTCAACGAGGTGCGT------------TA---------------AGAA-----CATCATCATCAAATAAACCGAAAGACAAAAC---------A
C---TCATCGTTC--G---A-TAGGCTTCCGGCAACAAATATGTCCCTCGTGCTGTCCTCGTCGACTTGGAGCCCGG
>JX091376_Talaromyces_flavovirens CBS 102801
GTGAGTTTG-A--CT-CGAC--------CCAAACTTTCTC-TCAATTGTCGCGAC-AAAACCCT------GACT-T-TTTCAGGCAA
ATCATCTCTGCTGAGCACGGTCTCGATGGCTCGGGTGTGTAA---------GTATT---CACCA-TTCG-AATCCAGCTACAA
TCCGACA--A-TGTCTGATCGTC-AACAGCTACAATGGCTCCTCCGACCTCCAGTTGGAGCGTATGAACGTCTACTT
CAACGAGGTGCGT------------CG----------------AAC-----AAAT-CCACCATAT-AACGA--A-ACAAATAAAGAAACAGAC---T
CACAA-TG--G--TA-TAGGCCTCCGGCAACAAATACGTCCCTCGTGCTGTCCTCGTCGACTTGGAGCCCGG
>JX494302_Talaromyces_flavus CBS 310.38
GTGAGTTTG-ACTCT-CGAC--------CGAAACTC-----TCAATTGTCGCGAC-AACACGCT------GACT-T-TTCCAGGCAAA
TCATCTCCGCTGAGCACGGTCTCGACGGCTCCGGTGTGTAA---------GTATT--ACACGA-TTCA-AATCCAGCTACGA
TCCAACA--A-TATCTGATAATC-AACAGCTACAATGGCTCCTCCGACCTCCAGTTGGAGCGTATGAACGTTTACTTC
AACGAGGTGCGT------------CA----------------AAC-----CACT-CCACCTAATAAACGGAAG-ACAA------------AC---TCATGA
TCG--A--TA-TAGGCTTCCGGCAACAAATATGTCCCTCGTGCTGTCCTCGTCGACTTGGAGCCCGG
>JX091383_Talaromyces_funiculosus CBS 272.86
GTGAGTTTA-ACTCA-AAAC------------------TA-TCAATTGGCGCAAC-AACACACT------GACTCG--TCCAGGCAAATCA
TCTCTGCTGAGCACGGTCTCGACGGCTCCGGTGTGTAA---------GTATT---CACGA-TCCG-AAACCAACTACAATCA
GACA--G-TATCTGATAATC-AACAGCTACAATGGCTCCTCCGATCTCCAGTTGGAGCGTATGAACGTCTACTTCAAC
GAGGTGCGT------------AA----------------AAC-----AAAT-TCATCGTACAAACGG--AAATAAA-----------TC---TCATAA-CG--
G--TC-CAGGCCTCCGGCAACAAATATGTCCCTCGTGCTGTCCTGGTCGATTTGGAGCCCGG
>JX091388_Talaromyces_galapagensis CBS 751.74
GTAAGTTTG-ACTGT-CAAC--------CCGAACTTTGCC-TCAGTTGTCGCGAC-AACACACT------GACT-T-CTCCAGGC
AAATCATCTCTGCTGAGCACGGCCTCGATGGCTCTGGTGTGTAA---------GTATT--GCACGA-TTCG-AATCTAGCTC
CAATCCGACA--A-TATCTGATTGTC-AATAGCTACAATGGCTCCTCCGACCTCCAGTTGGAGCGTATGAACGTCTAC
TTCAACGAGGTGCGT------------TA---------------TAAA-----ACAC-TCGACTTAT--GCAG--A-ACAA------------AC---ACTCA
T-TG--G--TA-TAGGCCTCCGGCAACAAATACGTTCCCCGTGCTGTCCTCGTCGACTTGGAGCCCGG
>JX494308_Talaromyces_indigoticus CBS 100534
GTGAGTTTG-ACTCC-CGAC--------CCAAACTTTCGA-TCAATTGTCACGAC-AACACGCT------GACT-T-TTCCAGGC

AAATCATCTCTGCTGAGCACGGTCTCGACGGCTCTGGTGTGTAA---------GTATT--ACACGT-TTCG-AATCCACCTA
CCATCCGGCA--G-TGTCTGATTATC-AACAGCTACAATGGCTCCTCCGACCTCCAGTTGGAGCGTATGAACGTCTA
CTTCAACGAGGTGCGT------------TA----------------GAA-----GACTCTCGACTCAT--GCAG--A-ACAAAC----------AC---TC
AT---TG--G--TA-TAGGCCTCCGGAAACAAATATGTCCCCCGTGCTGTCCTCGTCGACTTGGAGCCCGG
>JX091387_Talaromyces_intermedius CBS 152.65
GTGAGTTTG-ACTTT-GAAA--------ACAAACTCTGGG-TCAATTATCGCGAC-AACACGCT------GACTCT--TCCAGGCA
AATCATCTCTGCTGAGCACGGTCTCGACGGCTCTGGTGTGTAA---------GTATT-TACACGA-TTCGAAATCCGATTAC
AATCCGACA--A-TATCTGATAATC-AACAGTTACAATGGTTCCTCCGATCTCCAGTTGGAGCGTATGAACGTTTACTT
CAACGAGGTTCGT------------CA----------------GAA-----CAAT-CCGCCAAACAAATCAAAC-ACAAG-----------AC---TCAT
CACTG--G--TA-TAGGCTTCCGGCAACAAATATGTCCCCCGTGCTGTCCTCGTCGACTTGGAGCCCGG
>JX091380_Talaromyces_liani CBS 225.66
GTGAGTTGG-ACTCT-CGAC--------CCGAACTTTCTC-TCAATTGTCGCGAC-AACACGCT------GACT-T-TTCCAGGC
AAATCATCTCTGCTGAGCACGGTCTCGATGGCTCTGGTGTGTAA---------GTATT--GCACGA-TTCG-ACTCCAGCTA
CGATCCGACG--A-TATCTGATAATC-AACAGCTACAATGGCTCCTCCGACCTCCAGTTGGAGCGTATGAACGTTTAC
TTCAACGAGGTGCGT------------CA-----------------AC-----CAAT-CCATCGTATAAACGG--A-ACAAA-----------GC---TCATA
C-TG--G--TG-TAGGCCTCCGGCAACAAATACGTTCCCCGTGCTGTCCTCGTCGACTTGGAGCCCGG
>JX091382_Talaromyces_macrosporus CBS 317.63
GTGAGTTTG-ACTCT-CGGC--------CCAACCTTTCTA-TCAATGGTCGCGAC-AACACGCT------GACTTT-TTCCAGGC
AAATCATCTCCGCTGAGCACGGTCTCGACGGCTCTGGTGTGTAA---------GTATT---CACGA-TTCG-AATCCAGCGA
CAATCCGACA--A-TATCTGATGATC-AACAGCTACAATGGCTCCTCCGACCTCCAATTGGAGCGTATGAACGTCTAC
TTCAACGAGGTGCGT------------CA----------------AAC-----CAAT-TCATCGTATAAACGG--A-ATAAA-----------AC---TCATA
A-TG--G--AA-TAGGCCTCCGGCAACAAATATGTCCCCCGTGCTGTCCTCGTCGATTTGGAGCCCGG
>JX091389_Talaromyces_marneffei CBS 388.87
GTGAGTTTG-GCTCT-CAACAAACACATCACAACTTTCAG-TCACTTGTCGCGAC-AACACGCT------GACTCTTTTCC
AGGCAAATCATCTCCGCCGAGCACGGCCTCGACGGCTCCGGTGTGTAA---------GTATT--ACACGA-TTCA-AATCC
AGCTACAATCCGACA--ATTATCTGATGATC-AACAGCTACAATGGCTCCTCCGACCTCCAGTTGGAGCGTATGAAC
GTTTACTTCAACGAGGTGCGT------------CA----------------GAC-----CCTT-CCACCTAATAAACCGAAG-ACGA------------
ACTCATCATCATCG--A--TA-TAGGCTTCCGGCAACAAATATGTCCCCCGTGCTGTCCTCGTCGACTTGGAGCCCGG
>KJ865727_Talaromyces_muroii CBS 756.96
GTGAGTTTG-ACTCT-CGAC--------CCGAACTTTCTA-TCAATTGTCGCGAC-AACACGCT------GACT-T-TTCTAGGCA
AATCATCTCTGCTGAGCACGGTCTCGATGGCTCTGGTGTGTAA---------GTATT--GCACGA-TTCG-ATTCCAGCTAC
AATCCGACA--A-TATCTGATTATC-AACAGCTACAATGGCTCCTCCGACCTCCAGTTGGAGCGTATGAACGTCTACT
TCAACGAGGTGCGT------------TA---------------GAAA-----ACAC-TCGACTCAT--GCAG--A-ACAA------------AC---ACTCAT
-TT--G--AA-TAGGCCTCCGGCAACAAATACGTTCCCCGTGCTGTCCTCGTCGACTTGGAGCCCGG
>KJ775213_Talaromyces_oumae-annae CBS 138208
GTGAGTTTG-G-TCC-CGAC--------CCAAACTTTCTA-TCAATCGTCGCAAC-AACATGCT------GATT-C-TTTCAGGCAA
ATCATCTCTGCTGAGCACGGTCTCGACGGCTCTGGTGTGTAA---------GTGTT--ACACGA-TTCG-AATCCAGCTACA
ATCCGACA--A-TATCTGAC-ATC-AACAGCTACAATGGCTCCTCCGACCTCCAGTTGGAGCGTATGAACGTTTACTT
CAACGAGGTGCGT------------CA----------------GACCATCTCACC-ACACCAGATAACCCG--A-ACAAA-----------AC---TC
ATAATTG--G--TA-TAGGCCTCCGGCAACAAATATGTCCCTCGTGCTGTCCTCGTCGACTTGGAGCCCGG
>HQ156948_Talaromyces_panamensis CBS 128.89
GTGAGTTTG-ACGTT-AAACACA-----ACGAGCTTTACA-TCAATTGTCGCGAC-AATACGCT------GACTCT-TTCCAGG
CAAATCATCTCTGCTGAGCACGGTCTCGACGGCTCTGGTGTGTAA---------GTGTTGGACACGA-TTCGAACTCCG
GCTGCAATCCGACA--A-TTTTTAACGATC-AACAGTTACAATGGCTCCTCCGACCTCCAGTTGGAGCGTATGAACGT

CTACTTCAACGAGGTGCGT------------TA---------------GGAA-----CAGT-CGATC---CAAGCGAGAA-ACAA------------AC---TCATCAATT--G-ATA-TAGGCCCCCGGCAACAAATATGTCCCTCGTGCCGTCCTCGTCGACTTGGAGCCCGG

>JX091381_Talaromyces_pinophilus CBS 631.66

GTGAGTTGG-GCTCT-CGAC--------CTGGAATTTCTA-TCAATTGTCGTGAC-ACCACGTT------GACT-T-TTCCAGGCAAATCATCTCTGCTGAGCATGGCCTCGATGGCTCTGGTGTGTAA---------GTATT--ACACGA-TTCG-AATGCAGCTACAATCCGACA--A-GATCTGATAATC-AACAGCTACAATGGCTCCTCCGACCTCCAGTTGGAGCGTATGAACGTTTACTTCAACGAGGTGCGT------------CG----------------AAC-----CAAT-CCATTGTATAAG-GG--A-ACAAA-----------GC---TCATAC-TG--G--TG-TAGGCCTCCGGCAACAAATACGTTCCCCGTGCCGTCCTCGTCGACTTGGAGCCCGG

>JX494305_Talaromyces_primulinus CBS 321.48

GTGAGTCTG-AATCT-CGAC--------TCGAACTTTGTA-CTAATTGTCGCGAC-AATACGCT------GACTCT-TTCCAGGCAAATCATCTCTGCTGAGCACGGTCTCGACGGCTCTGGTGTGTAA---------GTATT--ACGCGA-TTCG-AACCCAGCTACAATTCGACA--A-TATCTGATCTTC-AACAGCTACAATGGCTCCTCCGACCTCCAGTTGGAGCGTATGAACGTCTACTTCAACGAGGTGCGT------------CA---------------GA-------CCAC-TTACCAGATAAACCG--A-ACGAAA----------AC---TCATAT-TT--T--AA-TAGGCCTCCGGCAACAAATATGTCCCCCGTGCTGTCCTCGTCGACTTGGAGCCCGG

>JX315639_Talaromyces_purpurogenus CBS 286.36

GTGAGGAATGA-----------------CCACGCTTTCAG-TCAATTGTCGCGAC-GACTCGCT------GACTAT-TTTCAGGCAAATCATCTCTGCTGAGCACGGTCTCGATGGATCCGGCGTGTAA---------GTGTT-GATGGGA-TTCGAAATCCATCTACAATTCGACC--G-TATCTGATAATC-AACAGTTACAATGGCTCCTCCGACCTCCAGTTGGAGCGTATGAACGTTTACTTCAACGAGGTGCGT------------CG----------------AAC-----AA---CCAACCAATAGAAACAAAAACAAAA----------AC---TCATATCCAATGCTTAACAGGCTTCCGGCAACAAATATGTTCCTCGTGCTGTCCTCGTCGACTTGGAACCCGG

>JX315629_Talaromyces_ruber CBS 132704

GTGAGTTTG-A--CT-CGAC--------CCAAGCTTACTA-TCAATTGTCGCGAC-AGCACGCT------GACT-A-GACCAGGCAAATCATCTCTGCTGAGCACGGTCTCGATGGCTCTGGTGTGTAA---------GTATT--TCACAA-TTGG-AATACACCTACAGTTCGACG--A-TATCTGATCATC-GACAGCTACAATGGCTCCTCCGACCTCCAGTTGGAGCGTATGAACGTCTACTTCAACGAGGTGCGT------------TA---------------GAAAG----TCTC-TCGACTCCT--ACAG--A-ACAG------------AC---ACTCAT-TC--A--TC-TAGGCCTCCGGCAACAAATATGTCCCTCGTGCCGTCCTCGTCGATTTGGAGCCCGG

>JX494309_Talaromyces_rubicundus CBS 342.59

GTGAGTTTGAACTCC-CGAC--------CCAACCTTTCCA-TCAATTGTCGCGAC-AACACGCT------GACT-T-TTCCAGGCAAATCATCTCTGCTGAGCACGGTCTCGACGGCTCTGGTGTGTAA---------GTATT--AGACCG-TACG-AATCCGACTACAATCCGACA--G-TATCTGATTATC-AACAGCTACAATGGCTCCTCCGACCTCCAGTTGGAGCGTATGAACGTCTACTTCAACGAGGTGCGT------------TA---------------GAAA-----ACAT-TCGACTCAT--GCAG--A-ACAAA-----------AC---ACTCAT-TG--G--TA-TAGGCCTCCGGAAACAAATATGTCCCCCGTGCTGTCCTCGTCGACTTAGAGCCCGG

>KJ775206_Talaromyces_sayulitensis CBS 138204

GTGAGTTGG-ACTCT-CGAC--------TCGGACTTTCTA-TCAATTGTCGCGAC-AACACGCT------GACT-T-TCCCAGGCAAATCATCTCTGCTGAGCACGGTCTCGATGGCTCTGGTGTGTAA---------GTATT--GCACGATTTCG-AATCCGGCTACAATCCGACG--A-TATCTGATAATC-AACAGCTACAATGGCTCCTCCGACCTCCAGTTGGAGCGTATGAACGTCTATTTCAACGAGGTGTGT------------CG----------------AAC-----CAAT-CCATCGTAT-AACGG--A-ACAAT-----------AC---TTATAT-TA--G--TA-TAGGCCTCCGGCAACAAATACGTTCCCCGTGCTGTCCTCGTCGACTTGGAACCCGG

>JX091379_Talaromyces_siamensis CBS 475.88

GTGAGTTTG-A--CT-CGAC--------CCAAACTTTCTA-TCAATTGTCGCGAC-AACACGCT------GACT-T-TTTCAGGCAAATCATCTCTGCTGAGCACGGTCTCGATGGCTCTGGTGTGTAA---------GTATT---CACGACCTCG-AATCCAGCTACAATCCGACA--A-TATCTGATCGTC-AACAGCTACAATGGCTCCTCCGACCTCCAGTTGGAGCGTATGAACGTCTACTTCAACGAGGTGCGT------------CG----------------TAC-----CAAT-CCACCATAT-GACGA--A-ACAA------------AC---TTACAA-TG--G--TATTAGGCCTCCGGCAACAAATACGTCCCCCGTGCTGTCCTCGTCGACTTGGAGCCCGG

>JX315633_Talaromyces_stollii CBS 408.93

GTGAGTTTG-A--CT-CGAC--------CCAAACTTTCTA-TCAATTGTCGCGGC-GGCACGCT------GACT-T-GTCCAGGCA

AATTATCTCTGCTGAGCACGGTCTCGATGGCTCTGGTGTGTAA---------GTATT--TCACAA-TTCG-AATACACCCACA

GTCCGAGG--T-TATCTGATCATC-GACAGCTACAATGGCTCCTCCGACCTCCAGTTGGAGCGTATGAACGTCTACTT

CAACGAGGTGCGT------------TT---------------GAAAG----TTTC-TCGACTCCT--GCAG--A-ACAA------------AC---ACTAAT-

TC--A--GC-TAGGCCTCCGGCAACAAATACGTCCCCCGTGCTGTCCTCGTCGACTTGGAGCCCGG

>JX494294_Talaromyces_thailandensis CBS 133147

GTGAGTGTTGACTCT-CGAC--------CCAAACTTTCGG-TCAATTCTCGCGAC-AACACGCT------GACTCT-TTCCAGG

CAAATCATCTCCGCTGAGCACGGTCTCGACGGCTCTGGTGTGTAA---------GTGTT-GATACGA-ATCGAAATTC---C

ACAATTCGACA--A-TATCTGATAATC-AACAGCTACAATGGCTCCTCCGACCTCCAGTTGGAGCGTATGAACGTTTA

CTTCAACGAGGTGAGT------------TA---------------GACA-----ACAC-TCGACTCG--AACAGAAC-ACAG------------AC---TC

ATCAATT--G--CA-TAGGCTTCCGGCAACAAGTATGTCCCTCGTGCTGTCCTCGTCGACTTGGAGCCCGG

>KF741928_Talaromyces_verruculosus CBS 388.48

GTGAGTTTG-ACTCT-GGAA--------CCCAACTTTCGA-TCAATTGTTGCGAC-AGCACACT------GACT-C-TTTCAGGCA

AATCATCTCTGCTGAGCACGGTCTCGACGGCTCCGGTGTGTAA---------GTATA--ACACGA-TTCG-AATCCAGCTGC

AGTCCGACG--A-TATCTGATGATC-AACAGCTACAATGGCTCCTCCGACCTCCAGTTGGAGCGTATGAACGTCTAC

TTCAACGAGGTGCGT------------CA----------------GAC-----CAAT-CCACTAGATAAATCG--A-ACAAA-----------AC---TCAT

CCCTC--G--A--TAGGCTTCCGGCAACAAATATGTCCCCCGTGCTGTCCTCGTCGACTTGGAGCCCGG

>JX494310_Talaromyces_viridis CBS 114.72

GTGAGTTTG-ACTCTGAGAC--------CGAAACTCTCAGTCAAATTGTT-------------T------GACTCT-TTCCAGGCAAATCA

TCTCTGCTGAGCACGGCCTCGATGGCTCCGGTGTGTAA---------GTATTTGACTTGG-TTCG-AATCCAGCTACAATC

CGACA--A-TCTCTGATCATC-AACAGTTACAATGGCTCCTCCGACCTCCAGTTGGAGCGTATGAACGTTTACTTCAA

CGAGGTGCGT------------TA---------------GAAAACAAGACAT-TCGACTCA--ACCGG----ACAAAG----------AC---TCATCA

GTT--G--GTTTAGGCTTCCGGCAACAAATATGTTCCTCGTGCTGTTCTCGTCGACTTGGAGCCCGG

>JX091385_Talaromyces_viridulus CBS 252.87

GTGAGTTTG-G-TCT-CGAC--------CCAAACTTTCTA-TCAATTGTCGCAAC-AACACGCT------GACT-C-TTTCAGGCA

AATCATCTCTGCTGAGCACGGTCTCGACGGCTCTGGTGTGTAA---------GTATC--GCACGA-TTCG-AATCCAGCTAC

AACCCGACA--A-TATCTGACAATC-AACAGCTACAATGGCTCCTCCGACCTCCAGTTGGAGCGTATGAACGTTTACT

TCAACGAG----GT------------CA----------------GAC-----CACT-CCACCAGATAAATCG--A-ACAAA-----------AC---TCATAAT

TG--G--TA-TAGGCCTCCGGCAACAAATATGTCCCTCGTGCTATCCTCGTCGACTTGGAGCCCGG

>KP765380_Talaromyces_qii_AS3.15414

GTGAGTGTTGACTCT-CGAC--------CCAAACTTTCAG-TCAATTCTCGCGAC-AACACGCT------GACTCT-TTCCAGG

CAAATCATCTCCGCTGAGCACGGTCTCGACGGCTCTGGTGTGTAA---------GTGTT-GATACGA-ATCGAAATCC---TA

CAATTCGACA--C-TATCTGATAATC-AACAGCTACAATGGCTCCTCCGACCTCCAGTTGGAGCGTATGAACGTTTAC

TTCAACGAGGTGCGT------------TA---------------GA-A-----TCAC-TCGACTCA--AACGAAAC-ACAG------------AC---TCAT

CAATT--G--CA-TAGGCTTCCGGCAACAAGTATGTCCCTCGTGCTGTCCTCGTCGATTTGGAGCCCGG

>KP765381_Talaromyces_fusisporus_AS3.15415

GTGAGTTGG-A--CA-CGAC--------CCAAACTTTCTA-TCAATTGTCGCGAC-AGCACGCT------GACT-T-GTC-AGGCAA

ATCATCTCTGCTGAGCACGGTCTCGATGGCTCTGGTGTGTAA---------GTATT--ATACGA-TTCG-AATCCAGCTACAA

TCCGACA--G-TATCTGATCATC-AACAGCTACAATGGCTCCTCCGACCTCCAGTTGGAGCGTATGAACGTCTACTTC

AACGAGGTGCGT------------TA---------------GGAT-----TTCC-TCGGCTCAT--GCAG--A-ACAA------------AC---ACTCAT-AT

--G--AA-TAGGCCTCCGGCAACAAATACGTCCCCCGTGCTGTCCTCGTCGACTTGGAGCCCGG

>KJ865725_Talaromyces_helicus_CBS_335.48

GTCCATCAT-ATTCT-TGTT-------------CCCTCTG-CTAATCGTCATAAC-------------------------CAGGCAAATCATCTCTGC

TGAGCACGGCCTCGATGGCTCTGGTGTGTAAGTTGACCGTGTATT-CTGTCGACGTCGAAATCTATCATCGATCGC

TCA--G-ATTCTGACCAACCACCAGCTACAATGGCTCCTCCGACCTTCAATTGGAGCGCATGAACGTCTACTTCAAC

GAGGTACGC------------CAATCCATCTAAGGCCTACACGCATTCAAT-CCAACTAACGCGAGAAAAAAAAA----------------

------------A--TCTTAGGCCAACGGCAACAAATATGTTCCTCGTGCTGTCCTCGTCGATCTCGAGCCCGG

Data S2. The sequence alignment of *BenA*.

Supplementary dataset 3

>KF741995_Talaromyces_aculeatus_CBS 289.48 its

CCTCCCACCCTTGTCTCT-ATAC-ACCTGTTGCTTTGGCGGGCCCACC-GGGGCCACCTGGTCGCCGGGGGACG--

CACGTCCCCGGGCCCGCGCCCGCCGAAG-CGCTCTGTGAACCCTGATGAAGATGG-GCTGTCTGAGTA-GTATG-

AAAATTGTCAAAACTTTCAACAATGGATCTCTTGGTTCCGGCATCGATGAAGAACGCAGCGAAATGCGATAAGTAA

TGTGAATTGCAGAATTCCGTGAATCATCGAATCTTTGAACGCACATTGCGCCCCCTGGCATTCCGGGGGGCATGC

CTGTCCGAGCGTCATTTCTGCCCTCAAGCACGGCTTGTGTGTTGGGTGCGGTCCCCCC-GGGGACCTGCCCGA

AAGGCAGCGGC-GACGTCCGTCT-GGTCCTCGAGCGTATGGGGCTTTGTCACTCG-CTCGGGAAGGACCTGCGG

GGGTTGGTCA

>JX315660_Talaromyces_amestolkiae CBS 132696

CCTCCCACCCTTGTCTCT-ATAC-ACCTGTTGCTTTGGCGGGCCCACC-GGGGCCACCTGGTCGCCGGGGGACA--

TCTGTCCCCGGGCCCGCGCCCGCCGAAG-CGCTCTGTGAACCCTGATGAAGATGG-GCTGTCTGAGTA-CTATG-A

AAATTGTCAAAACTTTCAACAATGGATCTCTTGGTTCCGGCATCGATGAAGAACGCAGCGAAATGCGATAAGTAAT

GTGAATTGCAGAATTCCGTGAATCATCGAATCTTTGAACGCACATTGCGCCCCCTGGCATTCCGGGGGGCATGCC

TGTCCGAGCGTCATTTCTGCCCTCAAGCACGGCTTGTGTGTTGGGTGCGGTCCCCCC-GGGGACCTGCCCGAAA

GGCAGCGGC-GACGTCCGTCT-GGTCCTCGAGCGTATGGGGCTTTGTCACTCG-CTCGGGAAGGACTGGCGGGG

GTTGGTCA

>KF183638_Talaromyces_angelicus KACC 46611

CCTCCCACCCTTGTCTCT-CTAC-ACCTGTTGCTTTGGCGGGCCCACC-GGGGCCACCTGGTCGCCGGGGGACG-

-CACGTCCCCGGGCCCGCGCCCGCCGAAG-CGCTCTGTGAACCCTGATGAAGATGG-GCTGTCTGAGTA-CGATG

-AAAATTGTCAAAACTTTCAACAATGGATCTCTTGGTTCCGGCATCGATGAAGAACGCAGCGAAATGCGATAAGTAA

TGTGAATTGCAGAATTCCGTGAATCATCGAATCTTTGAACGCACATTGCGCCCCCTGGCATTCCGGGGGGCATGC

CTGTCCGAGCGTCATTTCTGCCCTCAAGCACGGCTTGTGTGTTGGGTGTGGTCCCCCC-GGGGACCTGCCCGAA

AGGCAGCGGC-GACGTCCGTCT-GGTCCTCGAGCGTATGGGGCTCTGTCACTCG-CTCGGGAAGGACCTGCGGG

GGTTGGTCA

>JN899375_Talaromyces_apiculatus CBS 312.59

CCTCCCACCCTTGTC-CT-ATAC-ACCTGTTGCTTTGGCGGGCCCACC-GGGGCCACCTGGTCGCCGGGGGACG--

CACGTCCCCGGGCCCGCGCCCGCCGAAG-CGCTCTGTGAACCCTGATGAAGATGG-GCTGTCTGAG-A-TTATG-A

AAATTGTCAAAACTTTCAACAATGGATCTCTTGGTTCCGGCATCGATGAAGAACGCAGCGAAATGCGATAAGTAAT

GTGAATTGCAGAATTCCGTGAATCATCGAATCTTTGAACGCACATTGCGCCCCCTGGCATTCCGGGGGGCATGCC

TGTCCGAGCGTCATTTCTGCCCTCAAGCACGGCTTGTGTGTTGGGTGTGGTCCCCCC-GGGGACCTGCCCGAAA

GGCAGCGGC-GACGTCCGTC--GGTCCTCGAGCGTATGGGGCTT-GTCACTCG-CTCGGGAAGGACCTGCGGGGG

TTGGTCA

>JN899380_Talaromyces_aurantiacus CBS 314.59

CCTCCCACCCGTGTC----CTCTCACCTGTTGCTTTGGCGGGCCCACC-GGGGCCACCCGGTCGCCGGGGGACG--

TCCGTCCCCGGGCCCGCGCCCGCCGAAG-CGCTCTGTGAACCCTGATGAAGATGG-GCTGTCTGAG-C-GAATG-

AAAATTGTCAAAACTTTCAACAATGGATCTCTTGGTTCCGGCATCGATGAAGAACGCAGCGAAATGCGATAAGTAA

TGTGAATTGCAGAATTCCGTGAATCATCGAATCTTTGAACGCACATTGCGCCCCCTGGCATTCCGGGGGGCATGC

CTGTCCGAGCGTCATTTCTGCCCTCAAGCCCGGCTTGTGTGTTGGGCGTGGTCCCCCC-GGGGACCTGTCCGAA

AGGCAGCGGC-GACGTCCGTC--GGTCCTCGAGCGTATGGGGCTT-GTCACTCG-CTCGGGACGGATCGGCGGAG

GTTGGTCA

>JN899319_Talaromyces_calidicanius CBS 112002

CCTCCCACCCTTGTCTCT-CTAC-ACCTGTTGCTTTGGCGGGCCCACC-GGGGCCACCCGGTCGCCGGGGGACG-

-TTCGTCCCCGGGCCCGCGCCCGCCGAAG-CGCCCTGTGAACCCTGATGAAGATGG-GCTGTCTGAGTA-CGATG-
AAAATTGTCAAAACTTTCAACAATGGATCTCTTGGTTCCGGCATCGATGAAGAACGCAGCGAAATGCGATAAGTAA
TGTGAATTGCAGAATTCCGTGAATCATCGAATCTTTGAACGCACATTGCGCCCCCTGGCATTCCGGGGGGCATGC
CTGTCCGAGCGTCATTTCTGCCCTCAAGCACGGCTTGTGTGTTGGGTGTGGTCCCCCC-GGGGACCTGCCCGAA
AGGCAGCGGC-GACGTCCGTCT-GGTCCTCGAGCGTATGGGGCTTTGTCACTCG-CTCGGGAAGGACCTGCGGG
GGTTGGTCA
>KF183639_Talaromyces_cnidii KACC 46617
CCTCCCACCCTTGTCTCT-ATAC-ACCCGTTGCTTTGGCGGGCCCACC-GGGGCCACCTGGTCGCCGGGGGACG-
-TCCGTCCCCGGGCCCGCGCCCGCCGAAG-CGCTCTGTGAACCCTGATGAAGATGG-GCTGTCTGAGTA-CTATG-
AAAATTGTCAAAACTTTCAACAATGGATCTCTTGGTTCCGGCATCGATGAAGAACGCAGCGAAATGCGATAAGTAA
TGTGAATTGCAGAATTCCGTGAATCATCGAATCTTTGAACGCACATTGCGCCCCCTGGCATTCCGGGGGGCATGC
CTGTCCGAGCGTCATTTCTGCCCTCAAGCACGGCTTGTGTGTTGGGCGCGGTCCCCCC-GGGGACCTGCCCGA
AAGGCAGCGGC-GACGTCCGTCT-GGTCCTCGAGCGTATGGGGCTCTGTCACTCG-CTCGGGAAGGACCTGCGG
GGGTTGGTCA
>JN899327_Talaromyces_derxii CBS 412.89
CCTCCCACCCTTGTC-CT-CTAC-ACCTGTTGCTTTGGCGGGCCCACC-GGGGCCACCCGGTCGCCGGGGGACG--
-TCGTCCCCGGGCCCGCGCCCGCCGAGG-CGCCCTGTGAACCCTGATGAAGATGG-GCTGTCTGAG-A-TCATG-A
AAATTGTCAAAACTTTCAACAATGGATCTCTTGGTTCCGGCATCGATGAAGAACGCAGCGAAATGCGATAAGTAAT
GTGAATTGCAGAATTCCGTGAATCATCGAATCTTTGAACGCACATTGCGCCCCCTGGCATTCCGGGGGGCATGCC
TGTCCGAGCGTCATTTCTGCCCTCAAGCACGGCTTGTGTGTTGGGTGTGGTCCCCCC-GGGGACCTGCCCGAAA
GGCAGCGGC-GACGTCCGTCC-GGTCCTCGAGCGTATGGGGCTT-GTCACTCG-CTCGGGAAGGACCTGCGGGG
GTTGGTCA
>JN899342_Talaromyces_duclauxii CBS 322.48
CCTCCCACCCGTGTC-CT-CTAC-ACCTGTTGCTTTGGCGGGCCCACC-GGGGCCACCCGGTCGCCGGGGGACG-
--TTGTCCCCGGGCCCGCGCCCGCCGAAG-CGCCCTGTGAACCCTGATGAAGATGG-GCTGTCTGAG-A-TGATG-A
AAATTGTCAAAACTTTCAACAATGGATCTCTTGGTTCCGGCATCGATGAAGAACGCAGCGAAATGCGATAAGTAAT
GTGAATTGCAGAATTCCGTGAATCATCGAATCTTTGAACGCACATTGCGCCCCCTGGCATTCCGGGGGGCATGCC
TGTCCGAGCGTCATTTCTGCCCTCAAGCACGGCTTGTGTGTTGGGTGTGGTCCCCC--GGGGACCTGCCCGAAA
GGCAGCGGC-GACGTCCGTC--GGTCCTCGAGCGTATGGGGCTC-GTCACTCG-CTCGGGAAGGACCTGCGGGG
GTTGGTCA
>AB176617_Talaromyces_euchlorocarpius PF 1203
CCTCCCACCCTTGTCTCT-ATAC-ACCTGTTGCTTTGGCGGGCCCACC-GGGGCCACCCGGTCGCCGGGGGACG-
-TCCGTCCCCGGGCCCGCGCCCGCCGAAG-CGCCCTGTGAACCCTGATGAAGATGG-GCTGTCTGAGTA-CTATG-
AAAATTGTCAAAACTTTCAACAATGGATCTCTTGGTTCCGGCATCGATGAAGAACGCAGCGAAATGCGATAAGTAA
TGTGAATTGCAGAATTCCGTGAATCATCGAATCTTTGAACGCACATTGCGCCCCATGGCATTCCGGGGGGCATGC
CTGTCCCAGCGTCATTTCTGCCCTCAAGCGCGGCTTGTGTGTTGGGTGCGGTCCCCCC-GGGGACCTGCCCGA
AAGGCAGCGGC-GACGTCCGTCC-GGTCCTCGAGCGCATGGGGCTTTGTCACTCGACTCGGGAAGGACCTGCG
GGGGTTGGTCA
>JN899392_Talaromyces_flavovirens CBS 102801
CCTCCCACCCTTGTC-CT-CTAC-ACCTGTTGCTTTGGCGGGCCCACC-GGGGCGACCTGGTCGCCGGGGGACG--
AACGTCCCCGGGCCCGCGCCCGCCGAAG-CGCTCTGTGAACCCTGATGAAGATGG-GCTGTCTGAG-A-CGATG-
AAAATTGTCAAAACTTTCAACAATGGATCTCTTGGTTCCGGCATCGATGAAGAACGCAGCGAAATGCGATAAGTAA
TGTGAATTGCAGAATTCCGTGAATCATCGAATCTTTGAACGCACATTGCGCCCCCTGGCATTCCGGGGGGCATGC
CTGTCCGAGCGTCATTTCTGCCCTCAAGCACGGCTTGTGTGTTGGGTGTGGTCCCCCC-GGGGACCTGCCCGAA

AGGCAGCGGC-GACGTCCGTC--GGTCCTCGAGCGTATGGGGCTT-GTCACTCG-CTCGGGAAGGACCTGCGGGG
GTTGGTCA
>JN899360_Talaromyces_flavus CBS 310.38
CCTCCCACCCTTGTC-CT-ATAC-ACCTGTTGCTTTGGCGGGCCCACC-GGGGCCACCTGGTCGCCGGGGGACG---
-CGTCTCCGGGCCCGCGCCCGCCGAAG-CGCTCTGTGAACCCTGATGAAGATGG-GCTGTCTGAG-A-CTATG-AA
AATTGTCAAAACTTTCAACAATGGATCTCTTGGTTCCGGCATCGATGAAGAACGCAGCGAAATGCGATAAGTAATG
TGAATTGCAGAATTCCGTGAATCATCGAATCTTTGAACGCACATTGCGCCCCCTGGCATTCCGGGGGGCATGCCT
GTCCGAGCGTCATTTCTGCCCTCAAGCACGGCTTGTGTGTTGGGTGCGGTCCCCCC-GGGGACCTGCCCAAAA
GGCAGCGGC-GACGCCCGTC--GGTCCTCGAGCGTATGGGGCTC-GTCACTCG-CTCGGGAAGGACCTGCGGGG
GTTGGTCA
>JN899377_Talaromyces_funiculosus CBS 272.86
CCTCCCACCCTTGTC-CT-CTAC-ACCTGTTGCTTTGGCGGGCCCAC--GGGGCC-CCTGGTCGCCGGGGGACG---
CCGTCCCCGGGCCCGCGCCCGCCGAAG-CGCTTCGTGAACCCTGATGAAGAAGG-GCTGTCTGAG-A-CTATG-A
AAATTGTCAAAACTTTCAACAATGGATCTCTTGGTTCCGGCATCGATGAAGAACGCAGCGAAATGCGATAAGTAAT
GTGAATTGCAGAATTCCGTGAATCATCGAATCTTTGAACGCACATTGCGCCCCCTGGCATTCCGGGGGGCATGCC
TGTCCGAGCGTCATTTCTGCCCTCAAGCACGGCTTGTGTGTTGGGTGTGGTCCCCC--GGGGACCTGCCCGAAA
GGCAGCGGC-GACGTCCGTC--GGTCCTCGAGCGTATGGGGCTC-GTCACTCG-CTCGGGAAGGACCTGCGGGG
GTTGGTCA
>JN899358_Talaromyces_galapagensis CBS 751.74
CCTCCCACCCTTGTC-CT-ATAC-ACCTGTTGCTTTGGCGGGCCCACC-GGGGCCACCTGGTCGCCGGGGGATA--
CACGTCCCCGGGCCCGCGCCCGCCGAAG-CGCCCTGTGAACCCTGATGAAGATGG-GCTGTCTGAG-A-CTGTG-
AAAATGGTCAAAACTTTCAACAATGGATCTCTTGGTTCCGGCATCGATGAAGAACGCAGCGAAATGCGATAAGTAA
TGTGAATTGCAGAATTCCGTGAATCATCGAATCTTTGAACGCACATTGCGCCCCCTGGCATTCCGGGGGGCATGC
CTGTCCGAGCGTCATTTCTGCCCTCAAGCACGGCTTGTGTGTTGGGTGTGGTCCCCCC-GGGGACCTGCCCGAA
AGGCAGCGGC-GACGTCCGTC--GGTCCTCGAGCGTATGGGGCTC-GTCACTCG-CTCGGGACGGACCTGCGGG
GGTTGGTCA
>JN899331_Talaromyces_indigoticus CBS 100534
CCTCCCACCCTTGTC-CT-ATATAACCTGTTGCTTTGGCGGGCCCACC--CTGTGGCCAGGTCGCCGGGGGACG---
TCGTCCCCGGGCCCGCGCCCGCCGAAG-CGCCCTGTGAACCCTGATGAAGATGG--CTGTCTGAGTC-GTATG-AG
AATCGT-AAAACTTTCAACAATGGATCTCTTGGTTCCGGCATCGATGAAGAACGCAGCGAAATGCGATAAGTAATGT
GAATTGCAGAATTCCGTGAATCATCGAATCTTTGAACGCACATTGCGCCCCCTGGCATTCCGGGGGGCATGCCTG
TCCGAGCGTCATTTCTGCCCTCAAGCACGGCTTGTGTGTTGGGTGTGGTCCCCCC-GGGGACCTGCCCGAAAG
GCAGCGGC-GACGTCCGTC--GGTCCTCGAGCGTATGGGGCTT-GTCACTCG-CTCGGGAAGGACCTGCGGGGGT
TGGTCA
>JN899332_Talaromyces_intermedius CBS 152.65
CCTCCCACCCTTGTC-CT-ATAC-ACCTGTTGCTTTGGCGGGCCCACC-GGGGCCACCTGGTCGCCGGGGGACG---
-CGTCCCCGGGCCCGCGCCCGCCGAAG-CGCCCTGTGAACCCTGATGAATATGG-GCTGTCTGAG-A-CTATG-AAA
ATTGTCAAAACTTTCAACAATGGATCTCTTGGTTCCGGCATCGATGAAGAACGCAGCGAAATGCGATAAGTAATGT
GAATTGCAGAATTCCGTGAATCATCGAATCTTTGAACGCACATTGCGCCCCCTGGCATTCCGGGGGGCATGCCTG
TCCGAGCGTCATTTCTGCCCTCAAGCACGGCTTGTGTGTTGGGTGTGGTCCCCCC-GGGGACCTGCCCGAAAG
GCAGCGGC-GACGTCCGTC--GGTCCTCGAGCGTATGGGGCTT-GTCACTCG-CTCGGGAAGGACCTGCGGGGGT
TGGTCA
>JN899395_Talaromyces_liani CBS 225.66
CCTCCCACCCTTGTCTCCTATAC-ACCTGTTGCTTTGGCGGGCCCACC-GGGGCCACCTGGTCGCCGGGGGACG-

-CACGTCCCCGGGCCCGCGCCCGCCGAAG-CGCGCTGTGAACCCTGATGAAGATGG-GCTGTCTGAG-A-CTATG-
AAAATTGTCAAAACTTTCAACAATGGATCTCTTGGTTCCGGCATCGATGAAGAACGCAGCGAAATGCGATAAGTAA
TGTGAATTGCAGAATTCCGTGAATCATCGAATCTTTGAACGCACATTGCGCCCCCTGGCATTCCGGGGGGCATGC
CTGTCCGAGCGTCATTTCTGCCCTCAAGCACGGCTTGTGTGTTGGGTGTGGTCCCCCC-GGGGACCTGCCCGAA
AGGCAGCGGC-GACGTCCGTC--GGTCCTCGAGCGTATGGGGCTC-GTCACTCG-CTCGGGAAGGACCTGCGGG
GGTTGGTCA
>JN899333_Talaromyces_macrosporus CBS 317.63
CCTCCCACCCTTGTC-CT-ATAC-ACCTGTTGCTTTGGCGGGCCCACTGGGGGCCCCCTGGTCGCCGGGGGACA--
TCCGTCCCCGGGCCCGCGCCCGCCGAAG-CGCTCTGTGAACCCTGATGAAGATGG-GCTGTCTGAG-A-TTATG-A
AAATTGTCAAAACTTTCAACAATGGATCTCTTGGTTCCGGCATCGATGAAGAACGCAGCGAAATGCGATAAGTAAT
GTGAATTGCAGAATTCCGTGAATCATCGAATCTTTGAACGCACATTGCGCCCCCTGGCATTCCGGGGGGCATGCC
TGTCCGAGCGTCATTTCTGCCCTCAAGCACGGCTTGTTTGTTGGGTGTGGTCCCCCC-GGGGACCTGCCCGAAA
GGCAGCGGC-GACGTCCGTC--GGTCCTCGAGCGTATGGGGCTC-GTCACTCG-CTCGGGAAGGACCTGCGGGG
GTTGGTCA
>JN899344_Talaromyces_marneffei CBS 388.87
CCTCCCACCCTTGTC-CT-ATAC-ACCTGTTGCTTTGGCGGGCCCACC-GGGGCCACCCGGTCGCCGGGGGACG--
-TTGTCCCCGGGCCCGCGCCCGCCGAAG-CGCCCTGTGAACCCTGATGAAGATGG-ACTGTCTGAG-A-CCATG-A
AAATTGTCAAAACTTTCAACAATGGATCTCTTGGTTCCGGCATCGATGAAGAACGCAGCGAAATGCGATAAGTAAT
GTGAATTGCAGAATTCCGTGAATCATCGAATCTTTGAACGCACATTGCGCCCCCTGGCATTCCGGGGGGCATGCC
TGTCCGAGCGTCATTTCTGCCCTCAAGCACGGCTTGTGTGTTGGGTGTGGTCCCTCC-GGGGACCTGCCCGAAA
GGCAGCGGC-GACGTCCGTC--GGTCCTCGAGCGTATGGGGCTC-GTCACTCG-CTCGGGAAGGACCTGCGGGG
GTTGGTCA
>JN899351_Talaromyces_muroii CBS 756.96
CCTCCCACCCTTGTCGTTTATA--ACCTGTTGCTTTGGCGGGCCCACC-GGGGCCACCTGGTCGCCGGGGGACA--
--CGTTCCCGGGCCCGTGCCCGCCGAAG-CGCCCCTAGAACTCTGGTGAAGCTAG-GCTGTCTGAGC--TTATG-AA
AATAGT-AAAACTTTCAACAATGGATCTCTTGGTTCCGGCATCGATGAAGAACGCAGCGAAATGCGATAAGTAATGT
GAATTGCAGAATTCCGTGAATCATCGAATCTTTGAACGCACATTGCGCCCCCTGGCATTCCGGGGGGCATGCCTG
TCCGAGCGTCATTTCTGCCCTCAAGCGCGGCTTGTGTGTTGGGTGTGGTCCCCCC-GGGGACCTGCCCGAAAG
GCAGCGGC-GACGTCCGTCG-GGTCCTCGAGCGCATGGGGCTT-GTCACTCG-CTCGGGAGGGACCCGCGGGG
GTTGGTCA
>KJ775720_Talaromyces_oumae-annae CBS 138208
CCTCCCACCCTTGTCTCT-ACAC-ACCTGTTGCTTTGGCGGGCCCACC-GGGGCCACCCGGTCGCCGGGGGACG
GTTTCGTCCCCGGGCCCGCGCCCGCCGAAG-CGCCCTGTGAACCCTGATGAAGATGG-GCTGTCTGAGTA-CTAT
G-AAAATTGTCAAAACTTTCAACAATGGATCTCTTGGTTCCGGCATCGATGAAGAACGCAGCGAAATGCGATAAGT
AATGTGAATTGCAGAATTCCGTGAATCATCGAATCTTTGAACGCACATTGCGCCCCCTGGCATTCCGGGGGGCAT
GCCTGTCCGAGCGTCATTTCTGCCCTCAAGCACGGCTTGTGTGTTGGGTGCGGTCCCCCC-GGGGACCTGCCC
GAAAGGCAGCGGC-GACGTCCGTCT-GGTCCTCGAGCGTATGGGGCTCTGTCACTCG-CTCGGGACGGACCTGC
GGGGGTTGGTCA
>JN899362_Talaromyces_panamensis CBS 128.89
CCTCCCACCCTTGTC-CT-ACAC-ACCTGTTGCTTTGGCGGGCCCACC-GGGGCCACCCGGTCGCCGGGGGACG-
---CGTCCCCGGGCCCGCGCCCGCCGAAG-CGCCCTGTGAACCCTGATGAAGATGG-GCTGTCTGAG-A-CGATG-A
AAATTGTCAAAACTTTCAACAATGGATCTCTTGGTTCCGGCATCGATGAAGAACGCAGCGAAATGCGATAAGTAAT
GTGAATTGCAGAATTCCGTGAATCATCGAATCTTTGAACGCACATTGCGCCCCCTGGCATTCCGGGGGGCATGCC
TGTCCGAGCGTCATTTCTGCCCTCAAGCACGGCTTGTGTGTTGGGTGCGGTCCCCCC-GGGGACCTGCCCGAAA

GGCAGCGGC-GACGCCCGTC--GGTCCTCGAGCGCATGGGGCTT-GTCACTCG-CTCGGGATGGACCTGCGGGG
GTTGGTCA
>AB176603_Talaromyces_paucisporus PF 1150
CCTCCCACCCTTGTCTCT-ATAC-ACCTGTTGCTTTGGCGGGCCCACC-GGGGTCACCTGGTCGCCGGGGGACG--
CACGTCCCCGGGCCCGCGCCCGCCGAAG-CGCTCTGTGAACCCTGATGAAGATGG-GCTGTCTGAGTA-CGATG-
AAAATCGTCAAAACTTTCAACAATGGATCTCTTGGTTCCGGCATCGATGAAGAACGCAGCGAAATGCGATAAGTAA
TGTGAATTGCANAATTCCGTGAATCATCGAATCTTTGAACGCACATTGCGCCCCCTGGCATTCCGGGGGGCATGC
CTGTCCGAGCGTCATTTCTGCCCTCAAGCACGGCTTGTGTGTTGGGTGTGGTCCCCCC-GGGGACCTGCCCGAA
AGGCAGCGGCGGACGTCCGTCT-GGTCCTCGAGCGCATGGGGCTCTGTCACTCG-CTCGGGAAGGACCTGCGG
GGGTTGGTCA
>JN899382_Talaromyces_pinophilus CBS 631.66
CCTCCCACCCTTGTC-CT-ATAC-ACCTGTTGCTTTGGCGGGCCCACC-GGGGCCACCTGGTCGCCGGGGGACG--
CACGTCTCCGGGCCCGCGCCCGCCGAAG-CGCTCTGTGAACCCTGATGAAGATGG-GCTGTCTGAG-A-CTGTG-
AAAATTGTCAAAACTTTCAACAATGGATCTCTTGGTTCCGGCATCGATGAAGAACGCAGCGAAATGCGATAAGTAA
TGTGAATTGCAGAATTCCGTGAATCATCGAATCTTTGAACGCACATTGCGCCCCCTGGCATTCCGGGGGGCATGC
CTGTCCGAGCGTCATTTCTGCCCTCAAGCACGGCTTGTGTGTTGGGTGTGGTCCCCCC-GGGGACCTGCCCGAA
AGGCAGCGGC-GACGTCCGTC--GGTCCTCGAGCGTATGGGGCTC-GTCACTCG-CTCGGGAAGGACCTGCGGG
GGTTGGTCA
>JN899317_Talaromyces_primulinus CBS 321.48
CCTCCCACCCTTGTCTCT-CTAC-ACCTGTTGCTTTGGCGGGCCCACC-GGGGCCACCCGGTCGCCGGGGGACG-
-CTCGTCCCCGGGCCCGCGCCCGCCGAAG-CGCCCTGTGAACCCTGATGAAGATGG-GCTGTCTGAGTA-TTATG-
AAAATTGTCAAAACTTTCAACAATGGATCTCTTGGTTCCGGCATCGATGAAGAACGCAGCGAAATGCGATAAGTAA
TGTGAATTGCAGAATTCCGTGAATCATCGAATCTTTGAACGCACATTGCGCCCCCTGGCATTCCGGGGGGCATGC
CTGTCCGAGCGTCATTTCTGCCCTCAAGCGCGGCTTGTGTGTTGGGTGCGGTCCCCCC-GGGGACCTGCCCGA
AAGGCAGCGGC-GACGTCCGTCC-GGTCCTCGAGCGTATGGGGCTTTGTCACTCG-CTCGGGAAGGACCTGCGG
GGGTTGGTCA
>JN899372_Talaromyces_purpurogenus CBS 286.36
CCTCCCACCCTTGTCCCA---AC-ACCTGTTGCTTCGGCGGGCCCACC-GGGGCCACCCGGTCGCCGGGGGACA--
TCCGTCCCCGGGCCCGCGCCCGCCGAGG-CGCTCTGTGAACCCTGATGAAGATGG-GCTGTCTGAG-G-ATATG-A
AAATTGTCAAAACTTTCAACAATGGATCTCTTGGTTCCGGCATCGATGAAGAACGCAGCGAAATGCGATAAGTAAT
GTGAATTGCAGAATTCCGTGAATCATCGAATCTTTGAACGCACATTGCGCCCCCTGGCATTCCGGGGGGCATGCC
TGTCCGAGCGTCATTTCTGCCCTCAAGCACGGCTTGTGTGTTGGGTGTGGT-CCCCC-GGGGACCTGCCCGAAA
GGCAGCGGC-GACGTCCGTC--GGTCCTCGAGCGTATGGGGCTC-GTCACTCG-CTCGGGAAGGACCTGCGGGG
GTTGGTCA
>JX315662_Talaromyces_ruber CBS 132704
CCTCCCACCCTTGTCTCT-ATAC-ACCTGTTGCTTTGGCGGGCCCACC-GGGGTCACCTGGTCGCCGGGGGACAA
-TCTGTCCCCGGGCCCGCGCCCGCCGAAG-CGCTCTGTGAACCCTGATGAAGATGG-GCTGTCTGAGTA-CTATG-
AAAATTGTCAAAACTTTCAACAATGGATCTCTTGGTTCCGGCATCGATGAAGAACGCAGCGAAATGCGATAAGTAA
TGTGAATTGCAGAATTCCGTGAATCATCGAATCTTTGAACGCACATTGCGCCCCCTGGCATTCCGGGGGGCATGC
CTGTCCGAGCGTCATTTCTGCCCTCAAGCACGGCTTGTGTGTTGGGTGCGGTCCCCCC-GGGGACCTGCCCGA
AAGGCAGCGGC-GACGTCCGTCT-GGTCCTCGAGCGTATGGGGCTTTGTCACTCG-CTCGGGAAGGGCTGGCGG
GGGTTGGTCA
>JN899384_Talaromyces_rubicundus CBS 342.59
CCTCCCACCCTTGTC-CT-ATA--ACCTGTTGCTTTGGCGGGCCCACC-GTGA------GGTCGCCGGGGGACG--AACG

TCCCCGGGCCCGCGCCCGCCGAAG-CGCCCTGTGAACCCTGATGAAGATGG-GCTGTCTGAG---GTACG-AAAAT
CGTCAAAACTTTCAACAATGGATCTCTTGGTTCCGGCATCGATGAAGAACGCAGCGAAATGCGATAAGTAATGTGA
ATTGCAGAATTCCGTGAATCATCGAATCTTTGAACGCACATTGCGCCCCCTGGCATTCCGGGGGGCATGCCTGTC
CGAGCGTCATTTCTGCCCTCAAGCACGGCTTGTGTGTTGGGTGTGGTCCCCCC-GGGGACCTGCCCGAAAGGC
AGCGGC-GACGTCCGTC--GGTCCTCGAGCGTATGGGGCTC-GTCACTCG-CTCGGGAAGGACCTGCGGGGGTTG
GTCA
>KJ775713_Talaromyces_sayulitensis CBS 138204
CCTCCCACCCTTGTCTCTAATAC-ACCTGTTGCTTTGGCGGGCCCACC-GGGGCCACCTGGTCGCCGGGGGACG-
-CACGTCCCCGGGCCCGCGCCCGCCGAAG-CGCTCTGTGAACCCTGATGAAGATGG-GCTGTCTGAGTA-CTGTG
AAAAATTGTCAAAACTTTCAACAATGGATCTCTTGGTTCCGGCATCGATGAAGAACGCAGCGAAATGCGATAAGTA
ATGTGAATTGCAGAATTCCGTGAATCATCGAATCTTTGAACGCACATTGCGCCCCCTGGCATTCCGGGGGGCATG
CCTGTCCGAGCGTCATTTCTGCCCTCAAGCACGGCTTGTGTGTTGGGTGTGGTCCCCCC-GGGGACCTGCCCGA
AAGGCAGCGGC-GACGTCCGTCT-GGTCCTCGAGCGTATGGGGCTCTGTCACTCG-CTCGGGAAGGACCTGCGG
GGGTTGGTCA
>JN899385_Talaromyces_siamensis CBS 475.88
CCTCCCACCCTTGTC-CT-ATAC-ACCCGTTGCTTTGGCGGGCCCACC-GGGGCCACCTGGTCGCCGGGGGACG--
TCCGTCCCCGGGCCCGCGCCCGCCGAAG-CGCTCTGTGAACCCTGATGAAGATGG-GCTGTCTGAG-A-CTATG-A
AAATTGTCAAAACTTTCAACAATGGATCTCTTGGTTCCGGCATCGATGAAGAACGCAGCGAAATGCGATAAGTAAT
GTGAATTGCAGAATTCCGTGAATCATCGAATCTTTGAACGCACATTGCGCCCCCTGGCATTCCGGGGGGCATGCC
TGTCCGAGCGTCATTTCTGCCCTCAAGCACGGCTTGTGTGTTGGGTGCGGTCCCCCC-GGGGACCTGCCCGAAA
GGCAGCGGC-GACGTCCGTC--GGTCCTCGAGCGTATGGGGCTC-GTCACTCG-CTCGGGAAGGACCTGCGGGG
GTTGGTCA
>JN899348_Talaromyces_stipitatus CBS 375.48
CCTCCCACCCTTGTCC---ATA--ACCTGTTGCTTTGGCGGGCCCACG-GGGA--ACCTGGTCGCCGGGGGACG---TC
GTCCCCGGGCCCGCGCCCGCCGAGG-CGCCCTTTGAACCCTGATGAAGATGG-GCTGTCTGAG-G-ATATG-AAAA
TTGTCAAAACTTTCAACAACGAATCTCTTGGTTCCGGCATCGATGAAGAACGCAGCGAAATGCGATAAGTAATGTG
AATTGCAGAATTCCGTGAATCATCGAATCTTTGAACGCACATTGCGCCCCCTGGCATTCCGGGGGGCATGCCTGT
CCGAGCGTCATTTCTGCCCTCAAGCACGGCTTGTGTGTTGGGCGTGGTCCCTCC-GGGGACCTGCCCGAAAGG
CAGCGGC-GACGTCCGTC--GGTCCTCGAGCGTATGGGGCTC-GTCACTCG-CTCGGGAAGGACCTGCGGGGGTT
GGTCA
>JX315674_Talaromyces_stollii CBS 408.93
CCTCCCACCCTTGTCTCT-ATAC-ACCTGTTGCTTTGGCGGGCCCACC-GGGGCCACCTGGTCGCCGGGGGACG--
TTCGTCCCCGGGCCCGCGCCCGCCGAAG-CGCTCTGTGAACCCTGATGAAGATGG-GCTGTCTGAGTA-CTATG-A
AAATTGTCAAAACTTTCAACAATGGATCTCTTGGTTCCGGCATCGATGAAGAACGCAGCGAAATGCGATAAGTAAT
GTGAATTGCAGAATTCCGTGAATCATCGAATCTTTGAACGCACATTGCGCCCCCTGGCATTCCGGGGGGCATGCC
TGTCCGAGCGTCATTTCTGCCCTCAAGCACGGCTTGTGTGTTGGGTGCGGTCCCCCC-GGGGGCCTGCCCGAA
AGGCAGCGGC-GACGTCCGTCT-GGTCCTCGAGCGTATGGGGCTTTGTCACTCG-CTCGGGAAGGACTGGCGGG
GGTTGGTCA
>JX898041_Talaromyces_thailandensis CBS 133147
CCTCCCACCCTTGTCTCT-ATAC-ACCTGTTGCTTTGGCGGGCCCACC-GGGGCCACCCGGTCGCCGGGGGACG-
-CTCGTCCCCGGGCCCGCGCCCGCCGAAG-CGCTCTAAGAACCCTGATGAAGATGG-GCTGTCTGAGTA-CTATG-
AAAATTGTCAAAACTTTCAACAATGGATCTCTTGGTTCCGGCATCGATGAAGAACGCAGCGAAATGCGATAAGTAA
TGTGAATTGCAGAATTCCGTGAATCATCGAATCTTTGAACGCACATTGCGCCCCCTGGCATTCCGGGGGGCATGC
CTGTCCGAGCGTCATTTCTGCCCTCAAGCACGGCTTGTGTGTTGGGTGTGGTCCCCCC-GGGGACCTGCCCGAA

AGGCAGCGGC-GACGTCCGTCT-GGTCCTCGAGCGTATGGGGCTTTGTCACTCG-CTCGGGAAGGACCTGCGGG
GGTTGGTCA
>KF741994_Talaromyces_verruculosus CBS 388.48
CCTCCCACCCTTGTCTCT-ATAC-ACCTGTTGCTTTGGCGGGCCCACC-GGGGCCACCTGGTCGCCGGGGGACG--
-TCGTCTCCGGGCCCGCGCCCGCCGAAG-CGCTCTGTGAACCCTGATGAAGATGG-GCTGTCTGAGTA-CTATG-A
AAATTGTCAAAACTTTCAACAATGGATCTCTTGGTTCCGGCATCGATGAAGAACGCAGCGAAATGCGATAAGTAAT
GTGAATTGCAGAATTCCGTGAATCATCGAATCTTTGAACGCACATTGCGCCCCCTGGCATTCCGGGGGGCATGCC
TGTCCGAGCGTCATTTCTGCCCTCAAGCACGGCTTGTGTGTTGGGTGCGGTCCCCCC-GGGGACCTGCCCGAAA
GGCAGCGGC-GACGTCCGTCT-GGTCCTCGAGCGTATGGGGCTCTGTCACTCG-CTCGGGACGGACCTGCGGGG
GTTGGTCA
>AF285782_Talaromyces_viridis CBS 114.72
TCTCCCACCCTTGTCTCT-CTAT-ACCTGTTGCTTCGGCGGGCCCACC-GGGACA--CTGGTCGCCGGGGGACG--T
CCGTCCCCGGGCCCGCGCCCGCCGAAGCCCCCCTGTGAACCCTGATGAATATGGAGCTGTCTGAGTATCAATG-
AAAATCGTCAAAACTTTCAACAATGGATCTCTTGGTTCCGGCATCGATGAAGAACGCAGCGAAATGCGATAAGTAA
TGTGAATTGCAGAATTCCGTGAATCATCGAATCTTTGAACGCACATTGCGCCCCCTGGCATTCCGGGGGGCATGC
CTGTCCGAGCGTCATTTCTGCCCTCAAGCGCGGCTTGTGTGTTGGGTGCGGTCCCCCC-GGGGACCCGCCCGA
AAGGCAGCGGC-GACGCCCGTCC-GGTCCTCGAGCGCATGGGGCTCTGTCACCCG-CTCGGGAAGGACCCGCG
GGGGTTGGTCA
>JN899314_Talaromyces_viridulus CBS 252.87
CCTCCCACCCTTGTCTCT-ATAC-ACCTGTTGCTTTGGCGGGCCCACC-GGGGCCACCTGGTCGCCGGGGGACG--
TTCGTCCCCGGGCCCGCGCCCGCCGAAG-CGCCCTGTGAACCCTGATGAAGATGG-GCTGTCTGAGTA-CGATG-
AAAATTGTCAAAACTTTCAACAATGGATCTCTTGGTTCCGGCATCGATGAAGAACGCAGCGAAATGCGATAAGTAA
TGTGAATTGCAGAATTCCGTGAATCATCGAATCTTTGAACGCACATTGCGCCCCCTGGCATTCCGGGGGGCATGC
CTGTCCGAGCGTCATTTCTGCCCTCAAGCACGGCTTGTGTGTTGGGTGTGGTCCCCCC-GGGGACCTGCCCGAA
AGGCAGCGGC-GACGTCCGTCG-GGTCCTCGAGCGCATGGGGCTTTGTCACTCG-CTCGGGAAGGACCTGCGG
GGGTTGGTCA
>KP765384_Talaromyces_qii_AS3.15414
CCTCCCACCCTTGTCTCT-ATAT-ACCTGTTGCTTTGGCGGGCCCACC-GGGGCCACCCGGTCGCCGGGGGACG--
CTCGTCCCCGGGCCCGCGCCCGCCGAAG-CGCTCTAAGAACCCTGATGAAGATGG-GCTGTCTGAGTA-ATTTG-A
AAATTGTCAAAACTTTCAACAATGGATCTCTTGGTTCCGGCATCGATGAAGAACGCAGCGAAATGCGATAAGTAAT
GTGAATTGCAGAATTCCGTGAATCATCGAATCTTTGAACGCACATTGCGCCCCCTGGCATTCCGGGGGGCATGCC
TGTCCGAGCGTCATTTCTGCCCTCAAGCACGGCTTGTGTGTTGGGTGTGGTCCCTCC-GGGGACCTGCCCGAAA
GGCAGCGGC-GACGTCCGTCT-GGTCCTCGAGCGTATGGGGCTCTGTCACTCG-CTCGGGAAGGACCTGCGGGG
GTTGGTCA
>KP765385_Talaromyces_fusisporus_AS3.15415
CCTCCCACCCTTGTCTCT-ATAC-ACCTGTTGCTTTGGCGGGCCCACCGGGGCTCACCTGGTCGCCGGGGGACG-
-TCTGTCCCCGGGCCCGCGCCCGCCGAAG-CGCTCTGAGAACCCTGATGAAGATGG-GCTGTCTGAGTA-CTATG-
AAAATTGTCAAAACTTTCAACAATGGATCTCTTGGTTCCGGCATCGATGAAGAACGCAGCGAAATGCGATAAGTAA
TGTGAATTGCAGAATTCCGTGAATCATCGAATCTTTGAACGCACATTGCGCCCCCTGGCATTCCGGGGGGCATGC
CTGTCCGAGCGTCATTTCTGCCCTCAAGCGCGGCTTGTGTGTTGGGTGCGGTCCCCCC-GGGGACCTGCCCGA
AAGGCAGCGGC-GACGTCCGTTTGGGTCCTCGAGCGTATGGGGCTTTGTCACTCG-CTCGGGACGGACCCGCG
GGGGTTGGTCA
>JN8993591_Talaromyces_helicus_CBS 335.48l
CCTCCCACCCGTGCCGTT-ATAATACCTGTTGCTTTGGCGGGCCCAC--GGGG-CACCTGGTCGCTGGGGGACG---

CCGTCCCCGGGCCCGCGCCCGCCGAAG-CACCCCCTGAACTCTGAAGAAGATAGGGCTGTCTGAG-A-CCTAG-A
AAATTGTCAAAACTTTCAACAATGGATCTCTTGGTTCCGGCATCGATGAAGAACGCAGCGAAATGCGATAAGTAAT
GTGAATTGCAGAATTCCGTGAATCATCGAATCTTTGAACGCACATTGCGCCCCCTGGCATTCCGGGGGGCATGCC
TGTCCGAGCGTCATTTCTGCCCTCAAGCACGGCTTGTGTGTTGGGTGTGGTCCCCCCGGGGGACCTGCCCCAA
AGGCAGCGGC-GACGCCCGTCG-GGTCCTCGAGCGCATGGGGCTT-GTCACTTG-CTCGGGAGGGACCTGCGGG
GGTTGGTCA

Data S3. The sequence alignment of *ITS1-5.8S-ITS2.*

Supplementary dataset 4

>Talaromyces_aculeatus_CBS_289.48CaMBenAITS

TG-GATATCTAT-TTGTC----------GCA----ATGTTG---TGGT-GGGTGGTT-AGCTGACTA---CG---GTT---------TGATG-AG

TAGGACAAGGATGGAGATGGTGAGT----------------------CCGC-----CACGAA--CAATAACC-AAAAGACCTTGA-CCGA

AGG-------TGATC-GCA--------ACGGACA-AATATTGA---------TAAAATT-GAATAGGCCAAATCACAACCAAGGAACTGG

GCACCGTCATGCGCTCTCTCGGCCAGAACCCCTCCGAATCCGAATTGCAGGACATGATCAACGAAGTCGACGCT

GACAACAACGGCACAATCGATTTCCCTGGTATGCTATA------------AATCCTTACGGGT-TATTA-----CGA------------TGG

CGG----T-ACTAACTGC--T-G---CAGAATTCTTGACAATGATGGCCCGCAAAATGAAGGATACCGACTCCGAGGAAGA

GATCCGCGAGGCTTTCAAGGTGTTTGACCGTGACAACAATGNGTGAGTTTG-ACTCTCG-AC--CCA------AGCTTTC

-TATCAATTGTCGCGAC-AACACGCTGACT------CT-TTCCAGGCAAATCATCTCTGCTGAGCATGGTCTCGATGGCT

CTGGTGTGTAA---------GTA-TC---CATGA-ATGG-AATCCAGCTACAATCCGACA--A-TATCTGA-TAATCAACAGCTAC

AATGGCTCCTCTGACCTCCAGTTGGAGCGTATGAACGTCTACTTCAACGAGGTGCGTCG----------------AAC-----CAA

T-CCACCAAAT---AACGA-A---ACAAA------------CTCAC-A--ATGG------TATAGGCCTCCGGCAACAAATACGTCCCCC

GTGCTGTCCTCGTCGACTTGGAGCCCGGNCCTCCCACCCTTGTCT-CTATAC-ACCTGTTGCTTTGGCGGGCCCA

CC-GGGGCCACCTGGTCGCCGGGGGAC-G-CACGTCCCCGGGCCCGCGCCCGCCGAAG-CGCTCTGTGAACCC

TGATGAAGATGG-GCTGTCTGAGTA-GTATG-AAAATTGTCAAAACTTTCAACAATGGATCTCTTGGTTCCGGCATCG

ATGAAGAACGCAGCGAAATGCGATAAGTAATGTGAATTGCAGAATTCCGTGAATCATCGAATCTTTGAACGCACAT

TGCGCCCCCTGGCATTCCGGGGGGCATGCCTGTCCGAGCGTCATTTCTGCCCTCAAGCACGGCTTGTGTGTTG

GGTGCGGTCCCCCC-GGGGACCTGCCCGAAAGGCAGCGGCGACGTCCGTCT-GGTCCTCGAGCGTATGGGGCT

TTGTCACTCG-CTCGGGAAGGACCTGCGGGGGTTGGTCA

>Talaromyces_amestolkiae_CBS_132696

TG-GAAACTTGGTTTGTC----------ACA----ATGTTG---GC-----GTGGTT-AGCTGACCAG-CCG---TTT---------TGATG-AA

TAGGACAAGGATGGAGATGGTGAGT-----------------------CGC-----CGCGAA--CAATAA-----AACACCTTGA-ACGAAC

G-------TTACC-GCA--------GCCAACA-GACATTGA---------CCCTATC-GGACAGGACAAATCACAACCAAGGAACTCG

GCACAGTCATGCGTTCTCTCGGCCAGAACCCCTCCGAATCCGAATTGCAGGACATGATCAACGAAGTCGACGCT

GACAACAACGGCACAATTGATTTCCCTGGTATGACTGA-----------------CCACCAAC-TCGCAATATTTGA------------TG

GCAG----T-ACTGACTGC--C-G---CAGAATTCTTGACAATGATGGCCCGCAAAATGAAGGATACCGACTCCGAGGAA

GAGATCCGTGAGGCTTTCAAGGTGTTTGATCGTGACAACAATGNGTGAGTTTG-A--CTCG-TC--CCA------AGC-TA

C-TATCAATTGTCGCGAC-AGCACGCTGACT-------T-ATCCAGGCAAATCATCTCTGCTGAGCACGGTCTCGATGGC

TCTGGTGTGTAA---------GTA-TT--TCACAG-TTCG-AATACACCTACAGTCCGACA--A-CATCTGA-TCATCGACAGCTA

CAATGGCTCCTCCGACCTCCAGTTGGAGCGTATGAACGTCTACTTCAACGAGGTGCGTTA---------------GAAA----GT

CTC-TCGACTCCT----ATAG-A---ACAGA------------CACTC-A--TTCA-----TC-TAGGCCTCCGGCAACAAATACGTCCCC

CGTGCCGTCCTCGTCGATTTGGAGCCCGGNCCTCCCACCCTTGTCT-CTATAC-ACCTGTTGCTTTGGCGGGCCC

ACC-GGGGCCACCTGGTCGCCGGGGGAC-A-TCTGTCCCCGGGCCCGCGCCCGCCGAAG-CGCTCTGTGAACCC

TGATGAAGATGG-GCTGTCTGAGTA-CTATG-AAAATTGTCAAAACTTTCAACAATGGATCTCTTGGTTCCGGCATCG

ATGAAGAACGCAGCGAAATGCGATAAGTAATGTGAATTGCAGAATTCCGTGAATCATCGAATCTTTGAACGCACAT

TGCGCCCCCTGGCATTCCGGGGGGCATGCCTGTCCGAGCGTCATTTCTGCCCTCAAGCACGGCTTGTGTGTTG

GGTGCGGTCCCCCC-GGGGACCTGCCCGAAAGGCAGCGGCGACGTCCGTCT-GGTCCTCGAGCGTATGGGGCT

TTGTCACTCG-CTCGGGAAGGACTGGCGGGGGTTGGTCA

>Talaromyces_angelicus_KACC_46611

TG-GATTTCCAC-TTGTC----------GTA----ATGGTG---------GGTGGTT-AGCTGACTAG--CG---TTT---------TGATG-AGTA

GGACAAGGATGGAGATGGTGAGT----------------------CCGC-----CACGAA--CGATAACC-AAAAGACCTTGA-ACAAAG

G-------TTATC-GCT--------ACGAACA-GATATTGA---------CTATATC-GAATAGGCCAAATCACGACCAAGGAACTGGGC

ACCGTCATGCGTTCCCTCGGCCAGAACCCCTCCGAATCCGAATTGCAGGACATGATCAACGAAGTCGACGCTGA
CAACAACGGCACAATCGATTTTCCTGGTATGATATA------------ATTGCTTACGGTT-TACTA-----CGA------------TGGCA
G----T-ACTAACTGC--C-G---CAGAATTCTTGACAATGATGGCCCGCAAAATGAAGGATACCGACTCCGAGGAAGAGA
TCCGCGAGGCTTTCAAGGTGTTTGACCGCGACAACAATGNGTGAGTTTT-A--CTCG-AC--CCG------AACTTTC-TAT
CAATTGTCGCGAC-AACACGCTGACT-------T-TTCCAGGCAAATCATCTCTGCTGAGCACGGTCTCGATGGCTCTG
GTGTGTAA---------GTA-TT--ACATGA-TTCG-ATTCCAGCTACAATCCAACA--A-TATCTGA-TCATCAACAGCTACAAT
GGCTCCTCCGACCTCCAGTTGGAGCGTATGAACGTCTACTTCAACGAGGTGCGTCA----------------GAC-----GATT-C
CACCATAT--AAACAA-A---ACAAA-----------ACTCAT-----CGG-----GTATAGGCCTCCGGCAACAAATACGTTCCCCGTG
CTGTCCTCGTCGACTTGGAGCCCGGNCCTCCCACCCTTGTCT-CTCTAC-ACCTGTTGCTTTGGCGGGCCCACC-
GGGGCCACCTGGTCGCCGGGGGAC-G-CACGTCCCCGGGCCCGCGCCCGCCGAAG-CGCTCTGTGAACCCTGA
TGAAGATGG-GCTGTCTGAGTA-CGATG-AAAATTGTCAAAACTTTCAACAATGGATCTCTTGGTTCCGGCATCGAT
GAAGAACGCAGCGAAATGCGATAAGTAATGTGAATTGCAGAATTCCGTGAATCATCGAATCTTTGAACGCACATTG
CGCCCCCTGGCATTCCGGGGGGCATGCCTGTCCGAGCGTCATTTCTGCCCTCAAGCACGGCTTGTGTGTTGGG
TGTGGTCCCCCC-GGGGACCTGCCCGAAAGGCAGCGGCGACGTCCGTCT-GGTCCTCGAGCGTATGGGGCTCT
GTCACTCG-CTCGGGAAGGACCTGCGGGGGTTGGTCA
>Talaromyces_apiculatus_CBS_312.59
TG-GATATCTAT-TTGTC----------GCA----ATGTTG---CCGT-GGGTGGTT-AGCTGACTAG--CG---GCT---------TGATG-A
GTAGGACAAGGATGGAGATGGTGAGT-----------------------CCGC-----CATGAA--CAATAACC-AAGAGACCTTGA-ACA
TAGG-------TGATC-GCA--------ACGAACA-AGTATTGA---------TAAAATC-GAATAGGCCAAATCACAACCAAGGAACTG
GGCACCGTCATGCGTTCTCTCGGCCAGAACCCCTCCGAATCCGAATTACAGGACATGATCAATGAGGTCGACGC
TGACAACAACGGCACAATCGATTTCCCTGGTATGATATA------------ATTGCTTACGGGT-TATTA-----CGA------------CG
GCAG----T-GCTAACTGC--C-G---CAGAATTCTTGACAATGATGGCCCGCAAAATGAAGGATACCGACTCCGAGGAA
GAGATCCGCGAGGCTTTCAAGGTGTTTGACCGTGACAACAATGNGTGAGTTTG-ACACTCG-ACA-CCA------AGCT
TTC-TATCAGTTGTCGCGTC-AACACGCTGACTC-----TG-TCCCAGGCAAATCATCTCTGCTGAGCACGGTCTCGAT
GGCTCCGGTGTGTAA---------GTA-TC---CACGG-TTCG-AATCCAGGTACAATTCGACA--A-TATCTAA-TAATCAACAG
CTACAATGGCTCCTCTGACCTCCAGTTGGAGCGTATGAACGTCTACTTCAACGAAGTGCGTCG---------------AAAC--
---CAAT-CCACCATAT---AACGA-A---ACAAA------------CTCAC-A--GTGG------TATAGGCCTCCGGCAACAAATACGTC
CCCCGTGCTGTCCTGGTCGACTTGGAGCCCGGNCCTCCCACCCTTGTC--CTATAC-ACCTGTTGCTTTGGCGGG
CCCACC-GGGGCCACCTGGTCGCCGGGGGAC-G-CACGTCCCCGGGCCCGCGCCCGCCGAAG-CGCTCTGTGA
ACCCTGATGAAGATGG-GCTGTCTGAG-A-TTATG-AAAATTGTCAAAACTTTCAACAATGGATCTCTTGGTTCCGGC
ATCGATGAAGAACGCAGCGAAATGCGATAAGTAATGTGAATTGCAGAATTCCGTGAATCATCGAATCTTTGAACGC
ACATTGCGCCCCCTGGCATTCCGGGGGGCATGCCTGTCCGAGCGTCATTTCTGCCCTCAAGCACGGCTTGTGTG
TTGGGTGTGGTCCCCCC-GGGGACCTGCCCGAAAGGCAGCGGCGACGTCCGTC--GGTCCTCGAGCGTATGGG
GCTT-GTCACTCG-CTCGGGAAGGACCTGCGGGGGTTGGTCA
>Talaromyces_aurantiacus_CBS_314.59
TGAAATATCTTC-CTGTC----------GCATAGTATTTTC---TCGT----------TGCTTACAGA-ATG---TTT---------TAATGAAATA
GGACAAGGATGGAGATGGTGAGT---------------------GACGAC-----TGCGACA-CACCAACG-ATGTCGTATCGA-AAGC
AGATTCAT--CTACGATCT--------ACGAATA-AATATTGA---------TAGAGTC-GGACAGGTCAAATCACAACCAAGGAAC
TGGGCACCGTCATGCGTTCCCTCGGCCAGAACCCCTCCGAATCCGAATTGCAAGACATGATCAACGAAGTTGAC
GCTGACAACAACGGCACAATTGATTTCCCTGGTACGATC--------------ATCACGCCGCAAC----CTGCTTTCAA----------
--TGGAAA----A-ACTGACCGC--C-G---CAGAATTCTTGACCATGATGGCCCGCAAAATGAAGGATACCGACTCCGAG
GAAGAGATCCGCGAGGCTTTCAAGGTGTTTGACCGCGACAACAACGNGTGTGTAAA-------------AAA------GACTT---
GGTCAATTGTCGCCACAAAAAAGCTGAAT-------T-TTCTAGGCAAATCATCTCTGCTGAGCACGGTCTCGATGGCTC
CGGTGTGTAA---------GTG-TTACAAACCA-TTCG-AATGCAGTTATAATCCGACA--C-CATCTGA-TCATCAATAGCTAC

AATGGCTCCTCCGACCTCCAGTTGGAGCGTATGAACGTCTACTTCAACGAGGTGCGTGG--------------AAAC-----AA
CCATCAGAAAAT--CAATCGAATGCTTGGA-----------ACTCATCT--CTCGA----ATATAGGCCTCCGGCAATAAGTACGT
TCCCCGTGCCGTCCTTGTCGACTTGGAGCCCGGNCCTCCCACCCGTGTCCTCTC----ACCTGTTGCTTTGGCGG
GCCCACC-GGGGCCACCCGGTCGCCGGGGGAC-G-TCCGTCCCCGGGCCCGCGCCCGCCGAAG-CGCTCTGTG
AACCCTGATGAAGATGG-GCTGTCTGAGCG--AATG-AAAATTGTCAAAACTTTCAACAATGGATCTCTTGGTTCCGG
CATCGATGAAGAACGCAGCGAAATGCGATAAGTAATGTGAATTGCAGAATTCCGTGAATCATCGAATCTTTGAACG
CACATTGCGCCCCCTGGCATTCCGGGGGGCATGCCTGTCCGAGCGTCATTTCTGCCCTCAAGCCCGGCTTGTGT
GTTGGGCGTGGTCCCCCC-GGGGACCTGTCCGAAAGGCAGCGGCGACGTCCGTC--GGTCCTCGAGCGTATGG
GGCTT-GTCACTCG-CTCGGGACGGATCGGCGGAGGTTGGTCA
>Talaromyces_cnidii_KACC_46617
TG-ACTTTCTAC-TTATC----------GCA----ATGTTG---TGGT--GGTGGTT-AGCTGACTAG-CCG---GTT---------TGATG-AA
TAGGACAAGGATGGAGATGGTGAGT----------------------CGCCACGAACACAAA--CAACAACC-AAACGACTTTGA-A
CAAAGG-------CTATC---A--------ACGGACA-GTTATTGA---------CTCTATC-GAATAGGTCAAATCACAACCAAGGAACT
GGGCACCGTCATGCGTTCCCTCGGCCAGAACCCCTCCGAATCCGAATTGCAGGACATGATCAACGAGGTCGACG
CTGACAACAACGGCACAATCGATTTCCCTGGTATGACAAA-------------GCGCACTACCAC-TCGCAATATCACA--------
----TGGCAG----T-ACTAACTGC--C-G---CAGAATTCCTGACAATGATGGCCCGCAAAATGAAGGATACCGACTCCGA
GGAAGAGATCCGCGAGGCCTTCAAGGTGTTTGACCGTGACAACAATGNGTGAGTTTG-A--CTCG-AC--CCA------A
ACTTTC-TATCAATTGTCGCGAC-AACACGCTGACT-------T-TTTCAGGCAAATCATCTCTGCTGAGCACGGTCTTGA
TGGCTCTGGTGTGTAA---------GTA-TT---CACGAATTCG-AATCCAGCTACAATCCGACA--A-TATCTGA-TCGTCAAC
AGCTACAATGGCTCCTCCGACCTCCAGTTGGAGCGTATGAACGTCTACTTCAACGAGGTGCGTCG----------------AA
C-----CAAT-CCACCATAT---AACGA-A---ACAAA------------CTCAC-A--ATGG-----TATTAGGCCTCCGGCAACAAATACG
TCCCCCGTGCTGTCCTCGTCGACTTGGAGCCCGGNCCTCCCACCCTTGTCT-CTATAC-ACCCGTTGCTTTGGCG
GGCCCACC-GGGGCCACCTGGTCGCCGGGGGAC-G-TCCGTCCCCGGGCCCGCGCCCGCCGAAG-CGCTCTGT
GAACCCTGATGAAGATGG-GCTGTCTGAGTA-CTATG-AAAATTGTCAAAACTTTCAACAATGGATCTCTTGGTTCCG
GCATCGATGAAGAACGCAGCGAAATGCGATAAGTAATGTGAATTGCAGAATTCCGTGAATCATCGAATCTTTGAAC
GCACATTGCGCCCCCTGGCATTCCGGGGGGCATGCCTGTCCGAGCGTCATTTCTGCCCTCAAGCACGGCTTGT
GTGTTGGGCGCGGTCCCCCC-GGGGACCTGCCCGAAAGGCAGCGGCGACGTCCGTCT-GGTCCTCGAGCGTAT
GGGGCTCTGTCACTCG-CTCGGGAAGGACCTGCGGGGGTTGGTCA
>Talaromyces_derxii_CBS_412.89
TG------CAGG-CTGTC----------GCA----TTTTTT---------TGTGGTTGAGGTCACTAA-CCGGTCTTT---------TAATG-AACA
GGACAAGGATGGAGATGGTGAGT---------------------GACCGA-----TACGGCA-TGCCGACGAATATTGTCTCGA-ATAA
AAG-------CTTGAATGT--------ACGGGCA-GATACTGA---------TAGACTC-GAACAGGTCAGATCACTACCAAGGAACT
GGGCACCGTCATGCGTTCCCTCGGCCAGAACCCCTCCGAATCCGAGTTGCAGGACATGATCAACGAAGTCGAC
GCTGACAACAACGGCACAATTGATTTCCCTGGTACGATTCA-------------TCACTCTAGATGGTGACA----TCGA---------
---CGATAA----AAACTGACAGC--C-A---CAGAATTCCTGACCATGATGGCCCGCAAAATGAAGGATACCGACTCCGAG
GAAGAGATCCGCGAGGCTTTCAAGGTGTTTGACCGCGACAACAACGNGTGAGTTCTGA--CTCG-ACTGTAA------A
AGTCTC-AGTCAATTGTCGCGACAAACATGCTGACT-------T-TACCAGGCAAATCATCTCTGCCGAGCACGGTCTCG
ACGGCTCTGGTGTGTAA---------GTG-TTGCAAACGA-CTCG-GATCCAAATACAAGCCGATAGTA-TATCTGA-TTATC
AACAGCTACAATGGCTCCTCCGACCTCCAGTTGGAGCGTATGAACGTCTACTTCAATGAGGTGCGTGA---------------
-AAT----TAACCACCGAAAAAAACCCATCGAATGCTTGAA------------CTGATGT--TTCGA----ATATAGGCCTCCGGCAA
CAAATACGTGCCCCGTGCTGTCCTCGTCGACTTGGAGCCCGGNCCTCCCACCCTTGTC--CTCTAC-ACCTGTTGC
TTTGGCGGGCCCACC-GGGGCCACCCGGTCGCCGGGGGAC-G--TCGTCCCCGGGCCCGCGCCCGCCGAGG-C
GCCCTGTGAACCCTGATGAAGATGG-GCTGTCTGAG-A-TCATG-AAAATTGTCAAAACTTTCAACAATGGATCTCTT
GGTTCCGGCATCGATGAAGAACGCAGCGAAATGCGATAAGTAATGTGAATTGCAGAATTCCGTGAATCATCGAATC

TTTGAACGCACATTGCGCCCCCTGGCATTCCGGGGGGCATGCCTGTCCGAGCGTCATTTCTGCCCTCAAGCACG
GCTTGTGTGTTGGGTGTGGTCCCCCC-GGGGACCTGCCCGAAAGGCAGCGGCGACGTCCGTCC-GGTCCTCGA
GCGTATGGGGCTT-GTCACTCG-CTCGGGAAGGACCTGCGGGGGTTGGTCA

>Talaromyces_duclauxii_CBS_322.48

TG-GATTCCGGG-TTGTC----------GCA----ATGTTG---TGGT-GGGTGGTT-AGCTGACTAG-CCG---TTT---------TGATG-
AATAGGACAAAGACGGAGATGGTGAGT---------------------GACGC-----CACGAA--CACCAACG-ATATACTCTTGGAA
CAAAGG-------CTATT-GCT--------GCGAACA-AATATTGA---------TTATGCC--AACAGGTCAAATCACAACCAAGGAACT
GGGCACCGTCATGCGTTCCCTCGGCCAGAACCCCTCCGAATCCGAATTGCAGGACATGATCAACGAGGTCGATG
CTGACAACAACGGCACAATCGATTTCCCTGGTATGATACA------GCTTCTACTTACCGCAACTGTTTCCGATCGCAA-
-----------GCGCAG----ATACTGACCGT--C-G---TAGAATTCTTGACAATGATGGCTCGCAAAATGAAGGATACCGACTC
TGAGGAAGAGATCCGCGAGGCTTTCAAGGTGTTTGACCGCGACAACAATGNGTGAGTTTG-ACCTTCA-CT--CCG-
-----AATTTTCAAGTCAATTGTCGCGAT-AACACGCTGACTCTGACTCT-TTCCAGGCAAATTATCTCTGCTGAGCACG
GTCTCGACGGCTCCGGTGTGTAA---------GTA-TT--ACTCAA-TTCA-AATCCAGCTACAATCCGACA--AGTATCTGA-T
AATCAACAGCTACAATGGCTCCTCCGACCTCCAGTTGGAGCGTATGAACGTTTACTTCAACGAGGTTCGTCA-------
---------GAC-----CCAT-CCACACCAT--AAACCGCA--AACAAG------------CTCAT-G--ATCG-----ATACAGGCTTCCGGCA
ACAAATATGTCCCCCGTGCTGTCCTCGTCGACTTGGAGCCCGGNCCTCCCACCCGTGTC--CTCTAC-ACCTGTTG
CTTTGGCGGGCCCACC-GGGGCCACCCGGTCGCCGGGGGAC-G--TTGTCCCCGGGCCCGCGCCCGCCGAAG-
CGCCCTGTGAACCCTGATGAAGATGG-GCTGTCTGAG-A-TGATG-AAAATTGTCAAAACTTTCAACAATGGATCTCT
TGGTTCCGGCATCGATGAAGAACGCAGCGAAATGCGATAAGTAATGTGAATTGCAGAATTCCGTGAATCATCGAAT
CTTTGAACGCACATTGCGCCCCCTGGCATTCCGGGGGGCATGCCTGTCCGAGCGTCATTTCTGCCCTCAAGCAC
GGCTTGTGTGTTGGGTGTGGTCCCCC--GGGGACCTGCCCGAAAGGCAGCGGCGACGTCCGTC--GGTCCTCGA
GCGTATGGGGCTC-GTCACTCG-CTCGGGAAGGACCTGCGGGGGTTGGTCA

>Talaromyces_euchlorocarpius_PF_1203

TGAGATACCCGG-TTGTCGC--------GCA----ATGTTATGTTGGT-GGGTGGTT-ATCTAACTAG-CCG---TTT---------TGAT
G-AATAGGACAAGGATGGAGATGGTGAGT--------------------GATCCGC-----CACGAA--CACCAGCG-ACATGGTCTTT
AGAACGAGG-------CTATACAGAATAGATACACGGACAGCATGTTGA---------TTATATC-GAATAGGTCAAATCACAAC
CAAGGAACTGGGCACCGTCATGCGTTCCCTCGGCCAGAACCCCTCCGAATCCGAATTGCAGGACATGATCAACG
AGGTCGACGCTGACAACAACGGCACAATCGATTTCCCTGGTATGATACC------------AGCACGTCTGCGC-TGAATAG
ATACGG------------TGGCGG----G-ACTAACTGC--CTA---TAGAATTCTTGACAATGATGGCCCGCAAAATGAAGGATAC
CGACTCCGAGGAAGAGATCCGCGAGGCCTTCAAGGTGTTTGACCGCGACAACAATGNGTGAGTTGG-ACTTTCG-
ACAAAAA------AACTTTC-AGTCAATTGTCGCGAC-ACCACGCTGACT------CT-TTGCAGGCAAATCATCTCCGCTGA
GCACGGTCTCGACGGCTCTGGTGTGTAA---------GTG-TT-GGCAAAA-TTCGAAATCCAGCTACAATCCGACA--A-TA
TCTGA-TAATCACCAGCTACAATGGCTCCTCCGACCTCCAGTTGGAGCGTATGAACGTCTACTTCAACGAGGTGC
GTTA---------------AGAA-----CATCATCATCAAAT--AAACCG-AAAGACAAA---------ACACTCATCG--TTCG-------ATAGG
CTTCCGGCAACAAATATGTCCCTCGTGCTGTCCTCGTCGACTTGGAGCCCGGNCCTCCCACCCTTGTCT-CTATA
C-ACCTGTTGCTTTGGCGGGCCCACC-GGGGCCACCCGGTCGCCGGGGGAC-G-TCCGTCCCCGGGCCCGCGC
CCGCCGAAG-CGCCCTGTGAACCCTGATGAAGATGG-GCTGTCTGAGTA-CTATG-AAAATTGTCAAAACTTTCAAC
AATGGATCTCTTGGTTCCGGCATCGATGAAGAACGCAGCGAAATGCGATAAGTAATGTGAATTGCAGAATTCCGTG
AATCATCGAATCTTTGAACGCACATTGCGCCCCATGGCATTCCGGGGGGCATGCCTGTCCCAGCGTCATTTCTGC
CCTCAAGCGCGGCTTGTGTGTTGGGTGCGGTCCCCCC-GGGGACCTGCCCGAAAGGCAGCGGCGACGTCCGT
CC-GGTCCTCGAGCGCATGGGGCTTTGTCACTCGACTCGGGAAGGACCTGCGGGGGTTGGTCA

>Talaromyces_flavovirens_CBS_102801

TG-GCTATCTAC-TTATC----------GCA----ATGTTG---TGG--GAGTGATT-AGCTGACTAG-CCC---CTT---------TGATG-AG
TAGGACAAGGACGGAGATGGTGAGT----------------------CGCCACGAACACGGA--CAACAACC-AAAAGATTCTCA-A

TAAAGG-------CTATC---G--------ACGAATA-AATGTTGA---------CTTTATT-GAATAGGTCAAATCACAACCAAGGAACTG
GGCACCGTCATGCGTTCTCTCGGCCAGAACCCCTCCGAATCCGAATTGCAGGACATGATCAACGAAGTCGACGC
TGACAACAACGGCACAATCGATTTTCCTGGTATGACAAA--------------GCACACTATCACGTCAATATTCCA-----------
TGGCAG----T-ACTAACTGC--C-G---CAGAATTCCTGACAATGATGGCCCGCAAAATGAAGGATACCGACTCCGAGG
AAGAGATCCGCGAGGCTTTCAAGGTGTTTGACCGCGACAACAATGNGTGAGTTTG-A--CTCG-AC--CCA------AAC
TTTC-TCTCAATTGTCGCGAC-AAAACCCTGACT-------T-TTTCAGGCAAATCATCTCTGCTGAGCACGGTCTCGATG
GCTCGGGTGTGTAA---------GTA-TT---CACCA-TTCG-AATCCAGCTACAATCCGACA--A-TGTCTGA-TCGTCAACAG
CTACAATGGCTCCTCCGACCTCCAGTTGGAGCGTATGAACGTCTACTTCAACGAGGTGCGTCG---------------AAC--
---AAAT-CCACCATAT---AACGA-A---ACAAATAAAGAAACAGACTCAC-A--ATGG-----TA-TAGGCCTCCGGCAACAAAT
ACGTCCCTCGTGCTGTCCTCGTCGACTTGGAGCCCGGNCCTCCCACCCTTGTC--CTCTAC-ACCTGTTGCTTTGG
CGGGCCCACC-GGGGCGACCTGGTCGCCGGGGGAC-G-AACGTCCCCGGGCCCGCGCCCGCCGAAG-CGCTCT
GTGAACCCTGATGAAGATGG-GCTGTCTGAG-A-CGATG-AAAATTGTCAAAACTTTCAACAATGGATCTCTTGGTTC
CGGCATCGATGAAGAACGCAGCGAAATGCGATAAGTAATGTGAATTGCAGAATTCCGTGAATCATCGAATCTTTGA
ACGCACATTGCGCCCCCTGGCATTCCGGGGGGCATGCCTGTCCGAGCGTCATTTCTGCCCTCAAGCACGGCTT
GTGTGTTGGGTGTGGTCCCCCC-GGGGACCTGCCCGAAAGGCAGCGGCGACGTCCGTC--GGTCCTCGAGCGTA
TGGGGCTT-GTCACTCG-CTCGGGAAGGACCTGCGGGGGTTGGTCA
>Talaromyces_flavus_CBS_310.38
TG-GATTTCCGG-TTGTC----------GCA----ATGTGG---TGGT-GGGTGGTT-AGCTGACTAG-CCG---TTT---------GGATG-
AGTAGGACAAGGATGGAGATGGTGAGT----------------------GAAC-----CACGAA--CACCAACG-ATACAGCAACGA-AT
AAGTA-CTGTCGTTATG-ATT--------GCGAGCAAATTATTGA---------CGGGGACTGAACAGGTCAAATCACAACCAAG
GAACTGGGCACCGTCATGCGTTCCCTCGGCCAGAACCCCTCCGAATCCGAATTGCAGGACATGATCAACGAAGT
CGACGCTGACAACAACGGCACAATCGATTTCCCTGGTATGGCCAA-----------ACCTGTCTACGGT-TATTA-----CAA--
----------TGGCAG----A-ACTAACTGC--CAA---CAGAATTCTTGACAATGATGGCCCGCAAAATGAAGGATACCGATTCC
GAGGAAGAGATCCGCGAGGCTTTCAAGGTGTTTGACCGTGATAACAATGNGTGAGTTTG-ACTCTCG-AC--CGA---
---AACTCTC-----AATTGTCGCGAC-AACACGCTGACT-------T-TTCCAGGCAAATCATCTCCGCTGAGCACGGTCTCG
ACGGCTCCGGTGTGTAA---------GTA-TT--ACACGA-TTCA-AATCCAGCTACGATCCAACA--A-TATCTGA-TAATCAAC
AGCTACAATGGCTCCTCCGACCTCCAGTTGGAGCGTATGAACGTTTACTTCAACGAGGTGCGTCA---------------AA
C-----CACT-CCACCTAAT--AAACGGAA--GACAAA-----------CTCAT-G--ATCG-----ATATAGGCTTCCGGCAACAAATAT
GTCCCTCGTGCTGTCCTCGTCGACTTGGAGCCCGGNCCTCCCACCCTTGTC--CTATAC-ACCTGTTGCTTTGGCG
GGCCCACC-GGGGCCACCTGGTCGCCGGGGGAC-G---CGTCTCCGGGCCCGCGCCCGCCGAAG-CGCTCTGTG
AACCCTGATGAAGATGG-GCTGTCTGAG-A-CTATG-AAAATTGTCAAAACTTTCAACAATGGATCTCTTGGTTCCGG
CATCGATGAAGAACGCAGCGAAATGCGATAAGTAATGTGAATTGCAGAATTCCGTGAATCATCGAATCTTTGAACG
CACATTGCGCCCCCTGGCATTCCGGGGGGCATGCCTGTCCGAGCGTCATTTCTGCCCTCAAGCACGGCTTGTGT
GTTGGGTGCGGTCCCCCC-GGGGACCTGCCCAAAAGGCAGCGGCGACGCCCGTC--GGTCCTCGAGCGTATGG
GGCTC-GTCACTCG-CTCGGGAAGGACCTGCGGGGGTTGGTCA
>Talaromyces_funiculosus_CBS_272.86
TG-GATTCCCGG-TTGTC----------GCA----ATGTTA--------TGGTGGTT-AGCTGACTAG-CCG---TTG---------AAATA-ATT
AGGACAAGGATGGAGATGGTGAGT----------------------CCGC-----CACGAA--TGATAACC-AGATGATCTTGA-GCAAA
GT-------TTATC-GCA--------TCGAATA-TATATTAA---------CTATATT-CAAAAGGCCAAATCACAACCAAGGAACTGGGC
ACTGTCATGCGTTCCCTCGGCCAGAACCCCTCCGAATCCGAATTGCAGGACATGATCAATGAAGTCGATGCTGAC
AACAACGGAACAATCGATTTCCCTGGTATGACAAA----------------CTGCAAAC-TCTCAGTTTTCTA-----------CCACA
G----T-ACTAACTAA--C-A---CAGAATTCTTGACTATGATGGCCCGCAAAATGAAGGATACTGACTCCGAGGAAGAGA
TCCGTGAGGCTTTCAAGGTGTTTGACCGTGACAACAATGNGTGAGTTTA-ACTCAAA-AC------------------TATCAATT
GGCGCAAC-AACACACTGACT-------C-GTCCAGGCAAATCATCTCTGCTGAGCACGGTCTCGACGGCTCCGGTGT

GTAA---------GTA-TT---CACGA-TCCG-AAACCAACTACAATCAGACA--G-TATCTGA-TAATCAACAGCTACAATGGCT
CCTCCGATCTCCAGTTGGAGCGTATGAACGTCTACTTCAACGAGGTGCGTAA----------------AAC-----AAAT-TCATCG
TAC--AAACGGAA---ATAAA-----------TCTCAT-A--ACGG-----TC-CAGGCCTCCGGCAACAAATATGTCCCTCGTGCTG
TCCTGGTCGATTTGGAGCCCGGNCCTCCCACCCTTGTC--CTCTAC-ACCTGTTGCTTTGGCGGGCCCAC--GGGG
CC-CCTGGTCGCCGGGGGAC-G--CCGTCCCCGGGCCCGCGCCCGCCGAAG-CGCTTCGTGAACCCTGATGAAG
AAGG-GCTGTCTGAG-A-CTATG-AAAATTGTCAAAACTTTCAACAATGGATCTCTTGGTTCCGGCATCGATGAAGAA
CGCAGCGAAATGCGATAAGTAATGTGAATTGCAGAATTCCGTGAATCATCGAATCTTTGAACGCACATTGCGCCCC
CTGGCATTCCGGGGGGCATGCCTGTCCGAGCGTCATTTCTGCCCTCAAGCACGGCTTGTGTGTTGGGTGTGGT
CCCCC--GGGGACCTGCCCGAAAGGCAGCGGCGACGTCCGTC--GGTCCTCGAGCGTATGGGGCTC-GTCACTCG
-CTCGGGAAGGACCTGCGGGGGTTGGTCA
>Talaromyces_galapagensis_CBS_751.74
TG-GATTTCTGG-TTGTC----------GCA----TTGTTT---TGGT-GGCTGGTT-AGCTGACTAG--CG---TTT---------TGATG-A
GTAGGACAAGGATGGTGATGGTGAGT----------------------CCGC-----CACGAA--CA-CGATG-ATATCGTCTCGA-ACAA
AGG-------TTGTT-TC---------ACGAACA-GATATTGA---------TAAACTC-CAATAGGCCAAATCACAACCAAGGAATTGG
GCACTGTCATGCGTTCCCTCGGTCAGAACCCCTCCGAATCTGAATTGCAGGACATGATCAACGAAGTCGACGCT
GACAACAACGGCACAATTGATTTCCCTGGTATGACAAACCACAA---------GCTCGCGATT-T---------TTA------------TGG
CGATACTT-ACTAACTGC--C-G---CAGAATTCTTGACGATGATGGCCCGTAAAATGAAGGATACCGACTCCGAGGAA
GAGATCCGTGAGGCATTCAAGGTGTTTGACCGTGACAACAATGNGTAAGTTTG-ACTGTCA-AC--CCG------AACTT
TG-CCTCAGTTGTCGCGAC-AACACACTGACT-------T-CTCCAGGCAAATCATCTCTGCTGAGCACGGCCTCGATGG
CTCTGGTGTGTAA---------GTA-TT--GCACGA-TTCG-AATCTAGCTCCAATCCGACA--A-TATCTGA-TTGTCAATAGCT
ACAATGGCTCCTCCGACCTCCAGTTGGAGCGTATGAACGTCTACTTCAACGAGGTGCGTTA--------------TAAA-----
ACAC-TCGACTTAT----GCAG-A---ACAAA------------CACTC-A--TTGG-----TA-TAGGCCTCCGGCAACAAATACGTTCC
CCGTGCTGTCCTCGTCGACTTGGAGCCCGGNCCTCCCACCCTTGTC--CTATAC-ACCTGTTGCTTTGGCGGGCC
CACC-GGGGCCACCTGGTCGCCGGGGGAT-A-CACGTCCCCGGGCCCGCGCCCGCCGAAG-CGCCCTGTGAAC
CCTGATGAAGATGG-GCTGTCTGAG-A-CTGTG-AAAATGGTCAAAACTTTCAACAATGGATCTCTTGGTTCCGGCA
TCGATGAAGAACGCAGCGAAATGCGATAAGTAATGTGAATTGCAGAATTCCGTGAATCATCGAATCTTTGAACGCA
CATTGCGCCCCCTGGCATTCCGGGGGGCATGCCTGTCCGAGCGTCATTTCTGCCCTCAAGCACGGCTTGTGTGT
TGGGTGTGGTCCCCCC-GGGGACCTGCCCGAAAGGCAGCGGCGACGTCCGTC--GGTCCTCGAGCGTATGGGG
CTC-GTCACTCG-CTCGGGACGGACCTGCGGGGGTTGGTCA
>Talaromyces_indigoticus_CBS_100534
TG-GATTCCTGG-TTGTC----------GCA----ATGTTG---TGGT-GGGTGGTT-AGCTGACTAG--CG---GTT---------TGATG-A
GTAGGACAAGGATGGTGATGGTGAGT----------------------TCGC-----CACGAA--CAACGACC-AAAAGACCTTGA-ACA
AAGG-------TCATT-GCT--------ACGAACA-GATATTGA---------CTATATC-GAATAGGTCAAATTACGACCAAGGAATTGG
GCACCGTCATGCGTTCCCTCGGCCAGAACCCCTCCGAATCCGAATTGCAGGACATGATCAACGAAGTCGACGCT
GACAACAACGGCACAATTGATTTCCCTGGTATGACGAA-------------CCACACCACCAC-TCGCAATATTACA------------
TAGCAG----T-ACTAACTGC--T-A---CAGAATTCTTGACAATGATGGCTCGCAAAATGAAGGATACCGACTCCGAGGA
AGAGATCCGCGAGGCTTTCAAGGTGTTTGACCGTGACAACAATGNGTGAGTTTG-ACTCCCG-AC--CCA------AAC
TTTC-GATCAATTGTCACGAC-AACACGCTGACT-------T-TTCCAGGCAAATCATCTCTGCTGAGCACGGTCTCGAC
GGCTCTGGTGTGTAA---------GTA-TT--ACACGT-TTCG-AATCCACCTACCATCCGGCA--G-TGTCTGA-TTATCAACA
GCTACAATGGCTCCTCCGACCTCCAGTTGGAGCGTATGAACGTCTACTTCAACGAGGTGCGTTA----------------GAA-
----GACTCTCGACTCAT----GCAG-A---ACAAA-------------CACTC--ATTG-----GTATAGGCCTCCGGAAACAAATATGTC
CCCCGTGCTGTCCTCGTCGACTTGGAGCCCGGNCCTCCCACCCTTGTC--CTATATAACCTGTTGCTTTGGCGGG
CCCACCCTGTG--GCCAGGTCGCCGGGGGAC-G--TCGTCCCCGGGCCCGCGCCCGCCGAAG-CGCCCTGTGAA
CCCTGATGAAGATGG--CTGTCTGAGTC-GTATG-AGAATCGT-AAAACTTTCAACAATGGATCTCTTGGTTCCGGCA

TCGATGAAGAACGCAGCGAAATGCGATAAGTAATGTGAATTGCAGAATTCCGTGAATCATCGAATCTTTGAACGCA
CATTGCGCCCCCTGGCATTCCGGGGGGCATGCCTGTCCGAGCGTCATTTCTGCCCTCAAGCACGGCTTGTGTGT
TGGGTGTGGTCCCCCC-GGGGACCTGCCCGAAAGGCAGCGGCGACGTCCGTC--GGTCCTCGAGCGTATGGGG
CTT-GTCACTCG-CTCGGGAAGGACCTGCGGGGGTTGGTCA
>Talaromyces_intermedius_CBS_152.65
TG-GATTCCTAG-TTGTC----------GCA----ATGTTATGCTGGT-GGGTGGCT-AGCTGATTAG-CCG---TTT---------TGATG
-AGTAGGACAAGGATGGAGATGGTGAGT--------------------GATCCGC-----CTCGAC--CACCGACG-AGATAGTCTTCG
AACAAAGGCTTT---TGTTCCGAT--------GTGGACAGCATATTGA---------CTATATCGGAATAGGTCAAATTACAACCAA
GGAGCTCGGCACCGTCATGCGTTCCCTCGGCCAGAACCCCTCCGAATCCGAATTGCAGGACATGATCAACGAAG
TCGACGCTGACAACAACGGCACAATTGATTTCCCTGGTATGACACA------------------CTACAAC-TCGCAATCGTCTA-
-----------TGGCAG----TTGCTAACTGCTAC-A---CAGAATTCTTAACAATGATGGCCCGCAAAATGAAGGATACCGACT
CCGAGGAAGAGATCCGGGAGGCTTTCAAGGTGTTTGACCGTGACAACAATGNGTGAGTTTG-ACTTTGA-AA--AC
A------AACTCTG-GGTCAATTATCGCGAC-AACACGCTGACT-------C-TTCCAGGCAAATCATCTCTGCTGAGCACGG
TCTCGACGGCTCTGGTGTGTAA---------GTATTT--ACACGA-TTCGAAATCCGATTACAATCCGACA--A-TATCTGA-TA
ATCAACAGTTACAATGGTTCCTCCGATCTCCAGTTGGAGCGTATGAACGTTTACTTCAACGAGGTTCGTCA----------
------GAA-----CAAT-CCGCCAAAC--AAATCAAA--CACAAG-----------ACTCATCA--CTGG------TATAGGCTTCCGGCAA
CAAATATGTCCCCCGTGCTGTCCTCGTCGACTTGGAGCCCGGNCCTCCCACCCTTGTC--CTATAC-ACCTGTTGC
TTTGGCGGGCCCACC-GGGGCCACCTGGTCGCCGGGGGAC-G---CGTCCCCGGGCCCGCGCCCGCCGAAG-CG
CCCTGTGAACCCTGATGAATATGG-GCTGTCTGAG-A-CTATG-AAAATTGTCAAAACTTTCAACAATGGATCTCTTG
GTTCCGGCATCGATGAAGAACGCAGCGAAATGCGATAAGTAATGTGAATTGCAGAATTCCGTGAATCATCGAATCT
TTGAACGCACATTGCGCCCCCTGGCATTCCGGGGGGCATGCCTGTCCGAGCGTCATTTCTGCCCTCAAGCACG
GCTTGTGTGTTGGGTGTGGTCCCCCC-GGGGACCTGCCCGAAAGGCAGCGGCGACGTCCGTC--GGTCCTCGAG
CGTATGGGGCTT-GTCACTCG-CTCGGGAAGGACCTGCGGGGGTTGGTCA
>Talaromyces_liani_CBS_225.66
TA-GCAATCTGG-TTGTC----------GCA----ATGTTG---TGGT-GGGTGGTT-AGCTGACTAG-CCG---TTT---------TGATG-
AGTAGGACAAGGATGGAGATGGTGAGT----------------------CGGC-----CACGAA--CAATATCC-AAAAGGCGTTGA-AC
AAGGG-------TTATC-GCC--------GCGAACA-GATATTGA---------CTATATC-GAATAGGTCAAATCACAACCAAGGAACT
GGGCACTGTCATGCGTTCCCTCGGCCAGAACCCCTCCGAATCCGAACTGCAGGACATGATCAACGAAGTCGACG
CTGACAACAACGGCACAATCGATTTCCCTGGTATGATATA------------ATTGCTCACAGGA-TATTA-----CGA------------T
GGCAG----T-ACTAACTGC--C-G---CAGAATTCCTGACAATGATGGCCCGCAAAATGAAGGACACCGACTCCGAGGA
AGAGATCCGCGAGGCATTCAAGGTGTTTGACCGTGACAACAATGNGTGAGTTGG-ACTCTCG-AC--CCG------AAC
TTTC-TCTCAATTGTCGCGAC-AACACGCTGACT-------T-TTCCAGGCAAATCATCTCTGCTGAGCACGGTCTCGATG
GCTCTGGTGTGTAA---------GTA-TT--GCACGA-TTCG-ACTCCAGCTACGATCCGACG--A-TATCTGA-TAATCAACAG
CTACAATGGCTCCTCCGACCTCCAGTTGGAGCGTATGAACGTTTACTTCAACGAGGTGCGTCA-----------------AC----
-CAAT-CCATCGTAT--AAACGG-A---ACAAA-----------GCTCAT-A--CTGG------TGTAGGCCTCCGGCAACAAATACGTT
CCCCGTGCTGTCCTCGTCGACTTGGAGCCCGGNCCTCCCACCCTTGTCTCCTATAC-ACCTGTTGCTTTGGCGG
GCCCACC-GGGGCCACCTGGTCGCCGGGGGAC-G-CACGTCCCCGGGCCCGCGCCCGCCGAAG-CGCGCTGTG
AACCCTGATGAAGATGG-GCTGTCTGAG-A-CTATG-AAAATTGTCAAAACTTTCAACAATGGATCTCTTGGTTCCGG
CATCGATGAAGAACGCAGCGAAATGCGATAAGTAATGTGAATTGCAGAATTCCGTGAATCATCGAATCTTTGAACG
CACATTGCGCCCCCTGGCATTCCGGGGGGCATGCCTGTCCGAGCGTCATTTCTGCCCTCAAGCACGGCTTGTGT
GTTGGGTGTGGTCCCCCC-GGGGACCTGCCCGAAAGGCAGCGGCGACGTCCGTC--GGTCCTCGAGCGTATGG
GGCTC-GTCACTCG-CTCGGGAAGGACCTGCGGGGGTTGGTCA
>Talaromyces_macrosporus_CBS_317.63
TG-GATTCCTGG-TTGTC----------GCA----ATGT-----------GGTGATT-CGCTGACTAG-CCG----TT---------TGATG-CGCA

GGACAAGGATGGAGATGGTGAGT----------------------CCGC-----CACGAA--CAATAGCC-AAAAGGCCTCGA-ACAAA
GG-------CCGTC-GCT--------GCGAACA-GATATTGA---------CTATATC-GAATAGGCCAAATCACAACCAAGGAACTGG
GCACCGTCATGCGTTCCCTCGGCCAGAACCCCTCCGAATCCGAACTGCAGGACATGATCAACGAGGTCGATGCT
GACAACAACGGCACAATCGACTTCCCTGGTATGACAAA----------------CTACAAAC-TCGCA---TTCTA-----------TGG
CAG----T-ACTAACTGC--C-G---CAGAATTCTTGACAATGATGGCCCGCAAGATGAAGGATACCGACTCCGAGGAAG
AGATCCGCGAGGCTTTCAAGGTGTTTGACCGTGACAACAATGNGTGAGTTTG-ACTCTCG-GC--CCA------ACCTTT
C-TATCAATGGTCGCGAC-AACACGCTGACT------TT-TTCCAGGCAAATCATCTCCGCTGAGCACGGTCTCGACGG
CTCTGGTGTGTAA---------GTA-TT---CACGA-TTCG-AATCCAGCGACAATCCGACA--A-TATCTGA-TGATCAACAGCT
ACAATGGCTCCTCCGACCTCCAATTGGAGCGTATGAACGTCTACTTCAACGAGGTGCGTCA---------------AAC-----C
AAT-TCATCGTAT--AAACGG-A---ATAAA----------ACTCAT-A--ATGG------AATAGGCCTCCGGCAACAAATATGTCCCC
CGTGCTGTCCTCGTCGATTTGGAGCCCGGNCCTCCCACCCTTGTC--CTATAC-ACCTGTTGCTTTGGCGGGCCCA
CTGGGGGCCCCCTGGTCGCCGGGGGAC-A-TCCGTCCCCGGGCCCGCGCCCGCCGAAG-CGCTCTGTGAACCC
TGATGAAGATGG-GCTGTCTGAG-A-TTATG-AAAATTGTCAAAACTTTCAACAATGGATCTCTTGGTTCCGGCATCG
ATGAAGAACGCAGCGAAATGCGATAAGTAATGTGAATTGCAGAATTCCGTGAATCATCGAATCTTTGAACGCACAT
TGCGCCCCCTGGCATTCCGGGGGGCATGCCTGTCCGAGCGTCATTTCTGCCCTCAAGCACGGCTTGTTTGTTGG
GTGTGGTCCCCCC-GGGGACCTGCCCGAAAGGCAGCGGCGACGTCCGTC--GGTCCTCGAGCGTATGGGGCTC-
GTCACTCG-CTCGGGAAGGACCTGCGGGGGTTGGTCA
>Talaromyces_marneffei_CBS_388.87
TG-GATTTCTGG-TTGTC----------GCA----ATGTTG---TGGT-GGGTGGTT-CGCTGACTAG-CCG---TTT---------GGATG-
AATAGGACAAGGATGGTGATGGTGAGT---------------------GACGC-----CACGAA--CACCAGAC-ATATAGTCTTCGAA
CAAAAAG------TTATT-ACT--------GCGAACA-GATATTAA---------TAACATC--AATAGGTCAAATTACAACCAAGGAACT
GGGCACCGTCATGCGTTCCCTCGGCCAGAACCCTTCCGAATCCGAATTGCAGGACATGATCAACGAGGTCGACG
CTGACAACAACGGCACAATCGATTTCCCTGGTATGATGCA------GCCTCTATTTATCGCAGCCGTTTCCGATCATAA-
-----------GGGCAG----ATACTGACTGC--C-T---TAGAATTCTTGACAATGATGGCCCGCAAAATGAAGGATACCGACTC
CGAGGAAGAGATCCGCGAGGCTTTCAAGGTGTTTGATCGTGACAACAATGNGTGAGTTTG-GCTCTCA-ACAAAC
ACATCACAACTTTC-AGTCACTTGTCGCGAC-AACACGCTGACTC-----TT-TTCCAGGCAAATCATCTCCGCCGAGC
ACGGCCTCGACGGCTCCGGTGTGTAA---------GTA-TT--ACACGA-TTCA-AATCCAGCTACAATCCGACA--ATTATCT
GA-TGATCAACAGCTACAATGGCTCCTCCGACCTCCAGTTGGAGCGTATGAACGTTTACTTCAACGAGGTGCGTC
A---------------GAC-----CCTT-CCACCTAAT--AAACCGAA--GACGAA-----------CTCATCATCATCG-----ATATAGGCTTC
CGGCAACAAATATGTCCCCCGTGCTGTCCTCGTCGACTTGGAGCCCGGNCCTCCCACCCTTGTC--CTATAC-ACC
TGTTGCTTTGGCGGGCCCACC-GGGGCCACCCGGTCGCCGGGGGAC-G--TTGTCCCCGGGCCCGCGCCCGCC
GAAG-CGCCCTGTGAACCCTGATGAAGATGG-ACTGTCTGAG-A-CCATG-AAAATTGTCAAAACTTTCAACAATGGA
TCTCTTGGTTCCGGCATCGATGAAGAACGCAGCGAAATGCGATAAGTAATGTGAATTGCAGAATTCCGTGAATCAT
CGAATCTTTGAACGCACATTGCGCCCCCTGGCATTCCGGGGGGCATGCCTGTCCGAGCGTCATTTCTGCCCTCA
AGCACGGCTTGTGTGTTGGGTGTGGTCCCTCC-GGGGACCTGCCCGAAAGGCAGCGGCGACGTCCGTC--GGTC
CTCGAGCGTATGGGGCTC-GTCACTCG-CTCGGGAAGGACCTGCGGGGGTTGGTCA
>Talaromyces_muroii_CBS_756.96
TG-GATTTCTAC-TTGTC----------GCA----ATGTTG---TGGT-GGGTGGTT-AGCTGACTAG-CCG---TTT---------TGATG-A
GTAGGACAAGGATGGAGATGGTGAGT----------------------CCGC-----CACGAA--AAATAACC-AAAAGGCCTTGA-ACA
AAGG-------TTATC-GCT--------GCGAACA-GATATTGA---------CTATGTC-GAATAGGTCAAATCACAACCAAGGAACTG
GGCACCGTCATGCGTTCTCTCGGCCAGAACCCCTCCGAATCCGAATTGCAGGACATGATCAACGAAGTCGACGC
TGACAACAACGGCACAATCGATTTCCCTGGTATGACAAA----------------CCACAAGC-TCGCAATAATCTA-----------T
GGCAG----T-ACTAACTGC--C-G---CAGAATTCTTGACAATGATGGCCCGCAAAATGAAGGATACCGACTCCGAAGA
AGAGATCCGCGAGGCTTTCAAGGTGTTTGACCGCGACAACAATGNGTGAGTTTG-ACTCTCG-AC--CCG------AAC

TTTC-TATCAATTGTCGCGAC-AACACGCTGACT-------T-TTCTAGGCAAATCATCTCTGCTGAGCACGGTCTCGATG
GCTCTGGTGTGTAA---------GTA-TT--GCACGA-TTCG-ATTCCAGCTACAATCCGACA--A-TATCTGA-TTATCAACAGC
TACAATGGCTCCTCCGACCTCCAGTTGGAGCGTATGAACGTCTACTTCAACGAGGTGCGTTA---------------GAAA----
-ACAC-TCGACTCAT----GCAG-A---ACAAA------------CACTC-A--TTTG-----AA-TAGGCCTCCGGCAACAAATACGTTC
CCCGTGCTGTCCTCGTCGACTTGGAGCCCGGNCCTCCCACCCTTGTCGTTTATA--ACCTGTTGCTTTGGCGGGC
CCACC-GGGGCCACCTGGTCGCCGGGGGAC----ACGTTCCCGGGCCCGTGCCCGCCGAAG-CGCCCCTAGAACT
CTGGTGAAGCTAG-GCTGTCTGAG-C-TTATG-AAAATAGT-AAAACTTTCAACAATGGATCTCTTGGTTCCGGCATCG
ATGAAGAACGCAGCGAAATGCGATAAGTAATGTGAATTGCAGAATTCCGTGAATCATCGAATCTTTGAACGCACAT
TGCGCCCCCTGGCATTCCGGGGGGCATGCCTGTCCGAGCGTCATTTCTGCCCTCAAGCGCGGCTTGTGTGTTG
GGTGTGGTCCCCCC-GGGGACCTGCCCGAAAGGCAGCGGCGACGTCCGTCG-GGTCCTCGAGCGCATGGGGC
TT-GTCACTCG-CTCGGGAGGGACCCGCGGGGGTTGGTCA
>Talaromyces_oumae-annae_CBS_138208
TG-GATTTCTGA-TTGAC----------GCA----ATGTTT--GTGAT--CGTGGTT-AGCTGACTAG-CCG---TTT---------TGATG-A
GTAGGACAAGGATGGAGATGGTGAGT---------------------GACCGC-----CACGAA--CGTCGATG-ATACAGTCAACG-AA
TAAGGGCTATCCTTATG-ATT--------GCGAACAGCATATTGA---------TGGTATTTGAACAGGTCAGATCACCACCAAGG
AATTGGGCACCGTCATGCGTTCCCTCGGCCAGAACCCCTCCGAATCCGAATTGCAGGACATGATCAACGAGGTC
GACGCTGACAACAACGGCACAATCGATTTCCCTGGTATGACATA------ACCTATCAGACGCAGCCATATATGACTAT
CGAG------------TGGCAG----A-ACTAATTGC--C-A---CAGAATTCTTGACAATGATGGCCCGCAAAATGAAGGATACCG
ACTCCGAGGAAGAGATCCGTGAGGCTTTCAAGGTGTTTGACCGCGACAACAATGNGTGAGTTTG--GTCCCG-AC-
-CCA------AACTTTC-TATCAATCGTCGCAAC-AACATGCTGATT-------C-TTTCAGGCAAATCATCTCTGCTGAGCACG
GTCTCGACGGCTCTGGTGTGTAA---------GTG-TT--ACACGA-TTCG-AATCCAGCTACAATCCGACA--A-TATCTGA--
CATCAACAGCTACAATGGCTCCTCCGACCTCCAGTTGGAGCGTATGAACGTTTACTTCAACGAGGTGCGTCA-------
---------GACCATCTCACC-ACACCAGAT---AACCCGA---ACAAA-----------ACTCAT-A--ATTG-----GTATAGGCCTCCGG
CAACAAATATGTCCCTCGTGCTGTCCTCGTCGACTTGGAGCCCGGNCCTCCCACCCTTGTCT-CTACAC-ACCTGT
TGCTTTGGCGGGCCCACC-GGGGCCACCCGGTCGCCGGGGGACGGTTTCGTCCCCGGGCCCGCGCCCGCCG
AAG-CGCCCTGTGAACCCTGATGAAGATGG-GCTGTCTGAGTA-CTATG-AAAATTGTCAAAACTTTCAACAATGGAT
CTCTTGGTTCCGGCATCGATGAAGAACGCAGCGAAATGCGATAAGTAATGTGAATTGCAGAATTCCGTGAATCATC
GAATCTTTGAACGCACATTGCGCCCCCTGGCATTCCGGGGGGCATGCCTGTCCGAGCGTCATTTCTGCCCTCAA
GCACGGCTTGTGTGTTGGGTGCGGTCCCCCC-GGGGACCTGCCCGAAAGGCAGCGGCGACGTCCGTCT-GGTC
CTCGAGCGTATGGGGCTCTGTCACTCG-CTCGGGACGGACCTGCGGGGGTTGGTCA
>Talaromyces_panamensis_CBS_128.89
TG-GATTTTTAGTTGGTC----------GCA----ATGTTG---TGGT-GGGCTGTT-AGCTGACGCG-CCG---TGT---------TGATG-
AGTAGGACAAGGATGGCGATGGTGAGT--------------------GATCCGC-----CACGAG--CACCAACA-ATGCAGTTTTGA-
GCAAGGGATATGTATGATGAAAC--------ATGAACAACGGATTGA---------TTGTATCTAAATAGGTCAAATCACAACCAA
GGAACTCGGCACCGTCATGCGTTCCCTCGGCCAGAACCCCTCCGAATCCGAATTGCAGGACATGATCAACGAGG
TCGACGCCGACAACAACGGCACAATCGATTTCCCTGGTATGAGCCA------------ACCCGTCTACGTT-TGTTA-----CG
G------------GGGCAG----A-ACTAACTGC--CTA---CAGAATTCTTGACAATGATGGCTCGCAAAATGAAGGATACCGACT
CCGAAGAAGAGATCCGTGAGGCTTTCAAGGTGTTTGACCGCGACAACAATGNGTGAGTTTG-ACGTTAA-AC--AC
AACG---AGCTTTA-CATCAATTGTCGCGAC-AATACGCTGACT------CT-TTCCAGGCAAATCATCTCTGCTGAGCACG
GTCTCGACGGCTCTGGTGTGTAA---------GTG-TTGGACACGA-TTCGAACTCCGGCTGCAATCCGACA--A-TTTTTA
A-CGATCAACAGTTACAATGGCTCCTCCGACCTCCAGTTGGAGCGTATGAACGTCTACTTCAACGAGGTGCGTTA--
--------------GGA----------ACAGTCGATCCAAGCGAGA--AACAAA------------CTCATCA--ATTG-----ATATAGGCCCCCGG
CAACAAATATGTCCCTCGTGCCGTCCTCGTCGACTTGGAGCCCGGNCCTCCCACCCTTGTC--CTACAC-ACCTGT
TGCTTTGGCGGGCCCACC-GGGGCCACCCGGTCGCCGGGGGAC-G---CGTCCCCGGGCCCGCGCCCGCCGAA

G-CGCCCTGTGAACCCTGATGAAGATGG-GCTGTCTGAG-A-CGATG-AAAATTGTCAAAACTTTCAACAATGGATCT
CTTGGTTCCGGCATCGATGAAGAACGCAGCGAAATGCGATAAGTAATGTGAATTGCAGAATTCCGTGAATCATCGA
ATCTTTGAACGCACATTGCGCCCCCTGGCATTCCGGGGGGCATGCCTGTCCGAGCGTCATTTCTGCCCTCAAGC
ACGGCTTGTGTGTTGGGTGCGGTCCCCCC-GGGGACCTGCCCGAAAGGCAGCGGCGACGCCCGTC--GGTCCT
CGAGCGCATGGGGCTT-GTCACTCG-CTCGGGATGGACCTGCGGGGGTTGGTCA
>Talaromyces_pinophilus_CBS_631.66
TG-GAAATCTGG-TTGTC----------GCA----ATGTTG---TGGT-GGATGGTT-AGCTGACTAG-CCG---TTT---------TGATG-A
GTAGGACAAGGATGGAGATGGTGAGT----------------------CCGC-----CACGAA--CACGACGA-TATTTGTCTCGA-ACA
AAGG-------TTTTT-TC---------ACGAGCA-TATATTGA---------TAAAATC-TAATAGGCCAAATTACAACTAAGGAACTGGG
CACCGTTATGCGTTCCCTCGGCCAGAACCCCTCCGAATCCGAACTGCAGGACATGATCAACGAAGTCGACGCTG
ACAACAACGGCACAATCGATTTCCCTGGTATGATATA------------ATTGTTCACGGGT-T---TATA--CGA------------TGGC
AG----T-ACTAACTGC--C-G---CAGAATTCTTGACAATGATGGCCCGCAAAATGAAGGATACCGACTCCGAGGAAGA
GATCCGTGAAGCTTTCAAGGTGTTTGACCGTGACAACAATGNGTGAGTTGG-GCTCTCG-AC--CTG------GAATTTC
-TATCAATTGTCGTGAC-ACCACGTTGACT-------T-TTCCAGGCAAATCATCTCTGCTGAGCATGGCCTCGATGGCTC
TGGTGTGTAA---------GTA-TT--ACACGA-TTCG-AATGCAGCTACAATCCGACA--A-GATCTGA-TAATCAACAGCTACA
ATGGCTCCTCCGACCTCCAGTTGGAGCGTATGAACGTTTACTTCAACGAGGTGCGTCG----------------AAC-----CAAT
-CCATTGTAT---AAGGG-A---ACAAA-----------GCTCAT-A--CTGG-----TG-TAGGCCTCCGGCAACAAATACGTTCCCCG
TGCCGTCCTCGTCGACTTGGAGCCCGGNCCTCCCACCCTTGTC--CTATAC-ACCTGTTGCTTTGGCGGGCCCAC
C-GGGGCCACCTGGTCGCCGGGGGAC-G-CACGTCTCCGGGCCCGCGCCCGCCGAAG-CGCTCTGTGAACCCT
GATGAAGATGG-GCTGTCTGAG-A-CTGTG-AAAATTGTCAAAACTTTCAACAATGGATCTCTTGGTTCCGGCATCGA
TGAAGAACGCAGCGAAATGCGATAAGTAATGTGAATTGCAGAATTCCGTGAATCATCGAATCTTTGAACGCACATT
GCGCCCCCTGGCATTCCGGGGGGCATGCCTGTCCGAGCGTCATTTCTGCCCTCAAGCACGGCTTGTGTGTTGG
GTGTGGTCCCCCC-GGGGACCTGCCCGAAAGGCAGCGGCGACGTCCGTC--GGTCCTCGAGCGTATGGGGCTC-
GTCACTCG-CTCGGGAAGGACCTGCGGGGGTTGGTCA
>Talaromyces_primulinus_CBS_321.48
TT-CATTTCTGG-TTGTC----------GCA----ATGTTG---TGGT-GGGTGGTT-AGCTGACCAG-CCG---TTT---------TGATG-A
GTAGGACAAGGATGGAGATGGTGAGT---------------------GACCAC-------CGAA--CACCAACG-TTACAGTCAACG-AAT
AAGGGCTATCTTTATG-ATT--------GGGAACA-GGTATTAA-----------TCGTC-GAATAGGTCAAATCACAACCAAGGAAC
TGGGCACCGTCATGCGTTCCCTCGGCCAGAACCCCTCCGAATCCGAGTTGCAGGACATGATCAACGAAGTCGAC
GCTGACAACAACGGCACAATCGATTTCCCTGGTATGACTCA-----------------TCACGTCTATTGCTTGATACGA----------
--TGGCAG----T-ACTGACCGC--T-G---CAGAATTTTTGACAATGATGGCCCGCAAAATGAAGGATACCGACTCCGAG
GAAGAGATCCGCGAGGCTTTCAAGGTGTTTGACCGCGACAACAATGNGTGAGTCTG-AATCTCG-AC--TCG------A
ACTTTG-TACTAATTGTCGCGAC-AATACGCTGACT------CT-TTCCAGGCAAATCATCTCTGCTGAGCACGGTCTCGA
CGGCTCTGGTGTGTAA---------GTA-TT--ACGCGA-TTCG-AACCCAGCTACAATTCGACA--A-TATCTGA-TCTTCAAC
AGCTACAATGGCTCCTCCGACCTCCAGTTGGAGCGTATGAACGTCTACTTCAACGAGGTGCGTCA----------------GA
C-----CACT--TACCAGAT--AAACCG-A---ACGAA----------AACTCAT----ATTT-----TAATAGGCCTCCGGCAACAAATATG
TCCCCCGTGCTGTCCTCGTCGACTTGGAGCCCGGNCCTCCCACCCTTGTCT-CTCTAC-ACCTGTTGCTTTGGCG
GGCCCACC-GGGGCCACCCGGTCGCCGGGGGAC-G-CTCGTCCCCGGGCCCGCGCCCGCCGAAG-CGCCCTGT
GAACCCTGATGAAGATGG-GCTGTCTGAGTA-TTATG-AAAATTGTCAAAACTTTCAACAATGGATCTCTTGGTTCCG
GCATCGATGAAGAACGCAGCGAAATGCGATAAGTAATGTGAATTGCAGAATTCCGTGAATCATCGAATCTTTGAAC
GCACATTGCGCCCCCTGGCATTCCGGGGGGCATGCCTGTCCGAGCGTCATTTCTGCCCTCAAGCGCGGCTTGT
GTGTTGGGTGCGGTCCCCCC-GGGGACCTGCCCGAAAGGCAGCGGCGACGTCCGTCC-GGTCCTCGAGCGTAT
GGGGCTTTGTCACTCG-CTCGGGAAGGACCTGCGGGGGTTGGTCA
>Talaromyces_purpurogenus_CBS_286.36

TGCAATCATTGT-TTG------------GGT----ATGTTG------TTGGCCGGTT-ATCTAATTAGCCCG---TTT---------GGATG-AGT
AGGACAAGGATGGTGATGGTGAGT----------------------TCACCCGGACACGCAGCGATCAACG-ATAAGACTCTGA----
-------------------------------ACAGGATATTTA---------CTATATC-GATTAGGTCAAATCACAACCAAGGAACTGGGCACCGT
CATGCGCTCCCTCGGCCAGAACCCCTCCGAATCCGAATTGCAGGACATGATCAACGAAGTTGACGCTGACAACA
ACGGCACAATCGATTTCCCTGGTATGATGA------------CTCTCGCTACAATC-TACTGCGGATAGG------------TAACTG--
----ATTGATAAT--GGA---TAGAATTCTTGACAATGATGGCCCGCAAAATGAAGGATACCGACTCCGAGGAAGAGATC
CGTGAGGCTTTCAAGGTGTTTGACCGTGACAACAATGNGTGAGGAATGA-----------CCA------CGCTTTC-AGTCAAT
TGTCGCGAC-GACTCGCTGACT------AT-TTTCAGGCAAATCATCTCTGCTGAGCACGGTCTCGATGGATCCGGCGT
GTAA---------GTGTTG--ATGGGA-TTCGAAATCCATCTACAATTCGACC--G-TATCTGA-TAATCAACAGTTACAATGGC
TCCTCCGACCTCCAGTTGGAGCGTATGAACGTTTACTTCAACGAGGTGCGTCG----------------AAC-----AA---CCAAC
CAATAGAAACAAAA---ACAAA----------AACTCAT-A--TCCAATGCTTAACAGGCTTCCGGCAACAAATATGTTCCTCGT
GCTGTCCTCGTCGACTTGGAACCCGGNCCTCCCACCCTTGTC--CCA-AC-ACCTGTTGCTTCGGCGGGCCCACC-
GGGGCCACCCGGTCGCCGGGGGAC-A-TCCGTCCCCGGGCCCGCGCCCGCCGAGG-CGCTCTGTGAACCCTGA
TGAAGATGG-GCTGTCTGAGGA--TATG-AAAATTGTCAAAACTTTCAACAATGGATCTCTTGGTTCCGGCATCGATG
AAGAACGCAGCGAAATGCGATAAGTAATGTGAATTGCAGAATTCCGTGAATCATCGAATCTTTGAACGCACATTGC
GCCCCCTGGCATTCCGGGGGGCATGCCTGTCCGAGCGTCATTTCTGCCCTCAAGCACGGCTTGTGTGTTGGGT
GTGGTCCC-CC-GGGGACCTGCCCGAAAGGCAGCGGCGACGTCCGTC--GGTCCTCGAGCGTATGGGGCTC-GTC
ACTCG-CTCGGGAAGGACCTGCGGGGGTTGGTCA
>Talaromyces_ruber_CBS_132704
TG-GAAACTTGGTTTGTC----------ACA----ATGTTG---GC-----GTGGTT-AGCTGACTAG-CCG---TTT---------TGATG-AA
TAGGACAAGGATGGAGATGGTGAGT------------------------CGC-----CGCGAA--CAATGA-----AACACCTTGA-ACGAAC
A-------TTACC-GCA--------GTCAACA-GACATTGA---------CCCTATC-GGACAGGACAAATCACAACCAAGGAACTCGG
CACAGTCATGCGTTCTCTCGGCCAGAACCCCTCCGAATCCGAATTGCAGGACATGATCAACGAAGTCGACGCTG
ACAACAACGGCACAATTGATTTCCCTGGTATGACTGA-----------------CCACCAAC-TCGCAATATTTGA------------TGG
CAG----T-ACTGACTGC--C-G---CAGAATTCTTGACAATGATGGCCCGCAAAATGAAGGATACCGACTCCGAGGAAG
AGATCCGTGAGGCTTTCAAGGTGTTTGACCGTGACAACAATGNGTGAGTTTG-A--CTCG-AC--CCA------AGCTTAC
-TATCAATTGTCGCGAC-AGCACGCTGACT-------A-GACCAGGCAAATCATCTCTGCTGAGCACGGTCTCGATGGCT
CTGGTGTGTAA---------GTA-TT--TCACAA-TTGG-AATACACCTACAGTTCGACG--A-TATCTGA-TCATCGACAGCTAC
AATGGCTCCTCCGACCTCCAGTTGGAGCGTATGAACGTCTACTTCAACGAGGTGCGTTA---------------GAAA----GTC
TC-TCGACTCCT----ACAG-A---ACAGA------------CACTC-A--TTCA-----TC-TAGGCCTCCGGCAACAAATATGTCCCTC
GTGCCGTCCTCGTCGATTTGGAGCCCGGNCCTCCCACCCTTGTCT-CTATAC-ACCTGTTGCTTTGGCGGGCCCA
CC-GGGGTCACCTGGTCGCCGGGGGACAA-TCTGTCCCCGGGCCCGCGCCCGCCGAAG-CGCTCTGTGAACCC
TGATGAAGATGG-GCTGTCTGAGTA-CTATG-AAAATTGTCAAAACTTTCAACAATGGATCTCTTGGTTCCGGCATCG
ATGAAGAACGCAGCGAAATGCGATAAGTAATGTGAATTGCAGAATTCCGTGAATCATCGAATCTTTGAACGCACAT
TGCGCCCCCTGGCATTCCGGGGGGCATGCCTGTCCGAGCGTCATTTCTGCCCTCAAGCACGGCTTGTGTGTTG
GGTGCGGTCCCCCC-GGGGACCTGCCCGAAAGGCAGCGGCGACGTCCGTCT-GGTCCTCGAGCGTATGGGGCT
TTGTCACTCG-CTCGGGAAGGGCTGGCGGGGGTTGGTCA
>Talaromyces_rubicundus_CBS_342.59
TG-GATTTCTTC-TTGTC----------GCA----ATG------TGGT-GGGTGGTT-AGCTGACTAG--CG---GTT---------TAATG-AGT
AGGACAAGGATGGAGATGGTGAGT----------------------TCGC-----CATGAA--CAACGAAC-AAAAGACCTTGC-TCAAA
GG-------TCATT-GCT--------GCGAACA-GATATTGA---------CTATATC-GAATAGGTCAAATCACAACCAAGGAATTGGG
CACTGTCATGCGTTCCCTCGGCCAGAATCCCTCCGAATCCGAATTGCAGGACATGATCAACGAAGTCGACGCTG
ACAACAACGGCACAATCGATTTCCCTGGTATGACGAA-------------CCACACTACCAC-TCGCAATATTCCA------------T
AGCAG----T-ACTAACTGC--T-A---CAGAATTCTTGACAATGATGGCCCGCAAAATGAAGGATACCGACTCCGAAGAA

GAGATCCGTGAGGCTTTCAAGGTGTTCGATCGTGACAACAATGNGTGAGTTTGAACTCCCG-AC--CCA------ACCT
TTC-CATCAATTGTCGCGAC-AACACGCTGACT-------T-TTCCAGGCAAATCATCTCTGCTGAGCACGGTCTCGACG
GCTCTGGTGTGTAA---------GTA-TT--AGACCG-TACG-AATCCGACTACAATCCGACA--G-TATCTGA-TTATCAACAG
CTACAATGGCTCCTCCGACCTCCAGTTGGAGCGTATGAACGTCTACTTCAACGAGGTGCGTTA---------------GAAA--
---ACAT-TCGACTCAT----GCAG-A---ACAAA-------------ACACTC--ATTG-----GTATAGGCCTCCGGAAACAAATATGTCC
CCCGTGCTGTCCTCGTCGACTTAGAGCCCGGNCCTCCCACCCTTGTC--CTATA--ACCTGTTGCTTTGGCGGGCC
CACC-GTGA------GGTCGCCGGGGGAC-G-AACGTCCCCGGGCCCGCGCCCGCCGAAG-CGCCCTGTGAACCCTG
ATGAAGATGG-GCTGTCTGAG---GTACG-AAAATCGTCAAAACTTTCAACAATGGATCTCTTGGTTCCGGCATCGAT
GAAGAACGCAGCGAAATGCGATAAGTAATGTGAATTGCAGAATTCCGTGAATCATCGAATCTTTGAACGCACATTG
CGCCCCCTGGCATTCCGGGGGGCATGCCTGTCCGAGCGTCATTTCTGCCCTCAAGCACGGCTTGTGTGTTGGG
TGTGGTCCCCCC-GGGGACCTGCCCGAAAGGCAGCGGCGACGTCCGTC--GGTCCTCGAGCGTATGGGGCTC-G
TCACTCG-CTCGGGAAGGACCTGCGGGGGTTGGTCA
>Talaromyces_sayulitensis_CBS_138204
TG-CAAATCTGG-TTCTC----------GCA----ATGTTG---TGGT-GGGTGGTT-AGCTGACTAG-CCG---TTT---------TGATG-
AGTAGGACAAGGATGGAGATGGTGAGC-----------------------CCGC-----CACGAA--CAATAACC-AAAAAGCCTTGA-AC
AAAGG-------TTGGT-TC---------GCGAACA-AATATTGA----------TATGCTTGAATAGGCCAGATCACAACCAAGGAACT
GGGCACCGTCATGCGTTCTCTCGGCCAGAACCCCTCCGAATCCGAACTGCAGGACATGATCAATGAAGTGGACG
CTGACAACAACGGCACAATCGATTTTCCTGGTATGATATA------------ATTGCTCACGGAT-TATTA-----CGA------------T
GGCAG----T-ACTAATTGC--C-G---CAGAATTCTTGACAATGATGGCACGCAAAATGAAGGATACCGACTCGGAAGAA
GAGATCCGTGAGGCTTTCAAGGTGTTTGACCGTGACAACAATGNGTGAGTTGG-ACTCTCG-AC--TCG------GACT
TTC-TATCAATTGTCGCGAC-AACACGCTGACT-------T-TCCCAGGCAAATCATCTCTGCTGAGCACGGTCTCGATGG
CTCTGGTGTGTAA---------GTA-TT--GCACGATTTCG-AATCCGGCTACAATCCGACG--A-TATCTGA-TAATCAACAGC
TACAATGGCTCCTCCGACCTCCAGTTGGAGCGTATGAACGTCTATTTCAACGAGGTGTGTCG----------------AAC-----
CAAT-CCATCGTAT---AACGG-A---ACAAT-----------ACTTAT----ATTA-----GTATAGGCCTCCGGCAACAAATACGTTCCC
CGTGCTGTCCTCGTCGACTTGGAACCCGGNCCTCCCACCCTTGTCTCTAATAC-ACCTGTTGCTTTGGCGGGCCC
ACC-GGGGCCACCTGGTCGCCGGGGGAC-G-CACGTCCCCGGGCCCGCGCCCGCCGAAG-CGCTCTGTGAACC
CTGATGAAGATGG-GCTGTCTGAGTA-CTGTGAAAAATTGTCAAAACTTTCAACAATGGATCTCTTGGTTCCGGCAT
CGATGAAGAACGCAGCGAAATGCGATAAGTAATGTGAATTGCAGAATTCCGTGAATCATCGAATCTTTGAACGCAC
ATTGCGCCCCCTGGCATTCCGGGGGGCATGCCTGTCCGAGCGTCATTTCTGCCCTCAAGCACGGCTTGTGTGTT
GGGTGTGGTCCCCCC-GGGGACCTGCCCGAAAGGCAGCGGCGACGTCCGTCT-GGTCCTCGAGCGTATGGGGC
TCTGTCACTCG-CTCGGGAAGGACCTGCGGGGGTTGGTCA
>Talaromyces_siamensis_CBS_475.88
TG-ACTTTCTAC-TTATC----------GCA----ATGTTG---TGGT--GGTGGTT-AGCTGACTAG-CCG---GTT---------TGATG-AA
TAGGACAAGGATGGAGATGGTGAGT----------------------CGCCACGAACACAAA--CAACAACC-AAACGACTTTGA-A
CAAAGG-------CTATC---A--------ACGGACA-GTTATTGA---------CTCTATC-GAATAGGTCAAATCACAACCAAGGAACT
GGGCACCGTCATGCGTTCCCTCGGCCAGAACCCCTCGGAATCCGAATTGCAGGACATGATCAACGAGGTCGAC
GCTGACAACAACGGCACAATCGATTTTCCTGGTATGACAAA-------------GCACACAACTAC-TCGCAATATTCCA-------
-----TGGCAG----T-ACTAACTGC--T-G---CAGAATTCCTGACAATGATGGCCCGCAAAATGAAGGATACCGACTCCGA
GGAAGAGATCCGCGAGGCCTTCAAGGTGTTTGATCGTGACAACAATGNGTGAGTTTG-A--CTCG-AC--CCA------A
ACTTTC-TATCAATTGTCGCGAC-AACACGCTGACT-------T-TTTCAGGCAAATCATCTCTGCTGAGCACGGTCTCGA
TGGCTCTGGTGTGTAA---------GTA-TT---CACGACCTCG-AATCCAGCTACAATCCGACA--A-TATCTGA-TCGTCAAC
AGCTACAATGGCTCCTCCGACCTCCAGTTGGAGCGTATGAACGTCTACTTCAACGAGGTGCGTCG----------------TA
C-----CAAT-CCACCATAT---GACGA-A---ACAAA------------CTTAC-A--ATGG-----TATTAGGCCTCCGGCAACAAATACG
TCCCCCGTGCTGTCCTCGTCGACTTGGAGCCCGGNCCTCCCACCCTTGTC--CTATAC-ACCCGTTGCTTTGGCG

GGCCCACC-GGGGCCACCTGGTCGCCGGGGGAC-G-TCCGTCCCCGGGCCCGCGCCCGCCGAAG-CGCTCTGT
GAACCCTGATGAAGATGG-GCTGTCTGAG-A-CTATG-AAAATTGTCAAAACTTTCAACAATGGATCTCTTGGTTCCG
GCATCGATGAAGAACGCAGCGAAATGCGATAAGTAATGTGAATTGCAGAATTCCGTGAATCATCGAATCTTTGAAC
GCACATTGCGCCCCCTGGCATTCCGGGGGGCATGCCTGTCCGAGCGTCATTTCTGCCCTCAAGCACGGCTTGT
GTGTTGGGTGCGGTCCCCCC-GGGGACCTGCCCGAAAGGCAGCGGCGACGTCCGTC--GGTCCTCGAGCGTATG
GGGCTC-GTCACTCG-CTCGGGAAGGACCTGCGGGGGTTGGTCA
>Talaromyces_stollii_CBS_408.93
TG-GATTCCTTG-TTGTC----------GCA----ATGCTA---GCGT-GGGTGGTT-TGCTGACGAG-CCG---TCT---------TGATG-
AGTAGGACAAGGATGGAGATGGTGAGT------------------------CGC-----CGCGAA--CAATGA-----AGCTGCTTGA-ACGA
AT---------------CA--------GCCAACA-GACCTTAA---------CTCTATC-GAACAGGACAAATCACAACCAAGGAACTTGGCA
CCGTCATGCGTTCCCTCGGCCAGAACCCCTCCGAATCCGAATTGCAGGACATGATCAACGAAGTCGACGCTGAT
AACAACGGCACAATCGATTTCCCTGGTATGATAGA-----------------CCACCAGC-TCGCACTTTTTGA------------TGGCA
G----C-ACTAACTGC--C-G---CAGAATTTTTGACAATGATGGCCCGCAAAATGAAGGATACCGACTCCGAGGAAGAG
ATCCGTGAGGCTTTCAAGGTGTTTGACCGTGATAACAATGNGTGAGTTTG-A--CTCG-AC--CCA------AACTTTC-TA
TCAATTGTCGCGGC-GGCACGCTGACT-------T-GTCCAGGCAAATTATCTCTGCTGAGCACGGTCTCGATGGCTCT
GGTGTGTAA---------GTA-TT--TCACAA-TTCG-AATACACCCACAGTCCGAGG--T-TATCTGA-TCATCGACAGCTACAA
TGGCTCCTCCGACCTCCAGTTGGAGCGTATGAACGTCTACTTCAACGAGGTGCGTTT---------------GAAA----GTTTC
-TCGACTCCT----GCAG-A---ACAAA------------CACTA-A--TTCA-----GC-TAGGCCTCCGGCAACAAATACGTCCCCCG
TGCTGTCCTCGTCGACTTGGAGCCCGGNCCTCCCACCCTTGTCT-CTATAC-ACCTGTTGCTTTGGCGGGCCCAC
C-GGGGCCACCTGGTCGCCGGGGGAC-G-TTCGTCCCCGGGCCCGCGCCCGCCGAAG-CGCTCTGTGAACCCT
GATGAAGATGG-GCTGTCTGAGTA-CTATG-AAAATTGTCAAAACTTTCAACAATGGATCTCTTGGTTCCGGCATCGA
TGAAGAACGCAGCGAAATGCGATAAGTAATGTGAATTGCAGAATTCCGTGAATCATCGAATCTTTGAACGCACATT
GCGCCCCCTGGCATTCCGGGGGGCATGCCTGTCCGAGCGTCATTTCTGCCCTCAAGCACGGCTTGTGTGTTGG
GTGCGGTCCCCCC-GGGGGCCTGCCCGAAAGGCAGCGGCGACGTCCGTCT-GGTCCTCGAGCGTATGGGGCTT
TGTCACTCG-CTCGGGAAGGACTGGCGGGGGTTGGTCA
>Talaromyces_thailandensis_CBS_133147
TG-GGTTTCCGG-TTGTC----------GCA----ATGTTG---TGGCTGGGTGGTT-AGCTGACTAG-CCG---TTT---------TGATG
-AATAGGACAAGGATGGAGATGGTGAGT--------------------GAGCCGC-----AATGAT--CACCAACG-ATGCAATCTTTG-
GACAAGA-------ATATC-AGC--------ACGAACAGTATCCTGA---------CTATATCTGAATAGGTCAAATCACAACCAAGGA
ACTGGGCACCGTCATGCGTTCCCTCGGCCAGAACCCCTCCGAATCCGAGCTGCAGGACATGATCAACGAAGTC
GACGCTGACAACAACGGCACAATCGATTTCCCTGGTATGAAACA------------ATTGCTTACGGGC-G------TTACGA----
--------TGGCAG----AAACTAACTGC--CTA---CAGAATTCTTGACAATGATGGCCCGCAAAATGAAGGATACCGACTCC
GAGGAAGAGATCCGTGAGGCTTTCAAGGTGTTTGATCGCGACAACAATGNGTGAGTGTTGACTCTCG-AC--CCA--
----AACTTTC-GGTCAATTCTCGCGAC-AACACGCTGACT-------CTTTCCAGGCAAATCATCTCCGCTGAGCACGGTC
TCGACGGCTCTGGTGTGTAA---------GTG-TT-GATACGA-ATCGAAATTC---CACAATTCGACA--A-TATCTGA-TAATC
AACAGCTACAATGGCTCCTCCGACCTCCAGTTGGAGCGTATGAACGTTTACTTCAACGAGGTGAGTTA---------------
-GAC---------AACACTCGACTCGAACAGAA--CACAGA------------CTCATCA--ATTG-----CA-TAGGCTTCCGGCAACAA
GTATGTCCCTCGTGCTGTCCTCGTCGACTTGGAGCCCGGNCCTCCCACCCTTGTCT-CTATAC-ACCTGTTGCTTT
GGCGGGCCCACC-GGGGCCACCCGGTCGCCGGGGGAC-G-CTCGTCCCCGGGCCCGCGCCCGCCGAAG-CGC
TCTAAGAACCCTGATGAAGATGG-GCTGTCTGAGTA-CTATG-AAAATTGTCAAAACTTTCAACAATGGATCTCTTGG
TTCCGGCATCGATGAAGAACGCAGCGAAATGCGATAAGTAATGTGAATTGCAGAATTCCGTGAATCATCGAATCTT
TGAACGCACATTGCGCCCCCTGGCATTCCGGGGGGCATGCCTGTCCGAGCGTCATTTCTGCCCTCAAGCACGG
CTTGTGTGTTGGGTGTGGTCCCCCC-GGGGACCTGCCCGAAAGGCAGCGGCGACGTCCGTCT-GGTCCTCGAG
CGTATGGGGCTTTGTCACTCG-CTCGGGAAGGACCTGCGGGGGTTGGTCA

>Talaromyces_verruculosus_CBS_388.48

TG-AATTTCTGG-TTATC----------GCA----ATGTTG---TGGT-GGGTAGTT-AGCTGACTAG-CCG---TTT---------TGTTG-A
GTAGGACAAGGATGGAGATGGTGAGT---------------------GACCGC-----CACCAA--CACCGACG-ATCTAGTCTCGA-AC
AAAGG-------CTATT-TGT--------ACGATCA-GATATTGA---------CGATATCTGAATAGGTCAAATCACAACCAAGGAACT
GGGCACTGTCATGCGTTCCCTCGGCCAGAACCCCTCCGAATCCGAATTGCAGGACATGATCAACGAGGTTGACG
CTGACAACAACGGCACAATCGATTTCCCTGGTATGACATAACCTAT------CAACCGCAAACATATATGACAGTTGGA--
----------TGGCAG----A-ACTAACTGC--C-A---CAGAATTCTTGACAATGATGGCTCGCAAAATGAAGGATACCGACTCC
GAGGAAGAGATCCGTGAGGCTTTCAAGGTGTTTGACCGCGACAACAATGNGTGAGTTTG-ACTCTGG-AA--CCC---
---AACTTTC-GATCAATTGTTGCGAC-AGCACACTGACT-------C-TTTCAGGCAAATCATCTCTGCTGAGCACGGTCT
CGACGGCTCCGGTGTGTAA---------GTA-TA--ACACGA-TTCG-AATCCAGCTGCAGTCCGACG--A-TATCTGA-TGAT
CAACAGCTACAATGGCTCCTCCGACCTCCAGTTGGAGCGTATGAACGTCTACTTCAACGAGGTGCGTCA------------
----GAC-----CAAT-CCACTAGAT--AAATCG-A---ACAAA-----------ACTCATCC--CTCG-------ATAGGCTTCCGGCAACAA
ATATGTCCCCCGTGCTGTCCTCGTCGACTTGGAGCCCGGNCCTCCCACCCTTGTCT-CTATAC-ACCTGTTGCTTT
GGCGGGCCCACC-GGGGCCACCTGGTCGCCGGGGGAC-G--TCGTCTCCGGGCCCGCGCCCGCCGAAG-CGCT
CTGTGAACCCTGATGAAGATGG-GCTGTCTGAGTA-CTATG-AAAATTGTCAAAACTTTCAACAATGGATCTCTTGGT
TCCGGCATCGATGAAGAACGCAGCGAAATGCGATAAGTAATGTGAATTGCAGAATTCCGTGAATCATCGAATCTTT
GAACGCACATTGCGCCCCCTGGCATTCCGGGGGGCATGCCTGTCCGAGCGTCATTTCTGCCCTCAAGCACGGC
TTGTGTGTTGGGTGCGGTCCCCCC-GGGGACCTGCCCGAAAGGCAGCGGCGACGTCCGTCT-GGTCCTCGAGC
GTATGGGGCTCTGTCACTCG-CTCGGGACGGACCTGCGGGGGTTGGTCA

>Talaromyces_viridis_CBS_114.72

TG-TATTTCTTG-TTGTC----------GCA----ATGTTG---TGGT-GGGTGGTT-AGCTGACTAG-TCG---TTT---------TGATG-A
GTAGGACAAGGATGGAGATGGTGAGT--------------------CAACCGC-----CACG-------CAACG-ATACAATCTTTG-AACA
AAATCTACTATTGTA-ATT--------ACGAACAAAATATTGA---------CCATATCTGAAAAGGTCAAATCACAACCAAGGAAC
TGGGCACCGTCATGCGTTCCCTCGGCCAGAACCCCTCCGAATCCGAATTGCAGGACATGATCAACGAAGTCGAC
GCTGACAACAACGGCACAATCGATTTCCCTGGTATGACAGA--------------CACACTACAAC-GCACAATCGTCTA-------
-----TGGCAG----AAACTAACTGC--CGA---TAGAATTCTTGWCAATGATGGCCCGCAAAATGAAGGATACCGACTCCG
AGGAAGAGATCCGTGAGGCTTTCAAGGTGTTTGACCGTGACAACAATGNGTGAGTTTG-ACTCTGAGAC--CGA----
--AACTCTCAGTCAAATTGTT-------------TGACT------CT-TTCCAGGCAAATCATCTCTGCTGAGCACGGCCTCGATGG
CTCCGGTGTGTAA---------GTA-TTTGACTTGG-TTCG-AATCCAGCTACAATCCGACA--A-TCTCTGA-TCATCAACAG
TTACAATGGCTCCTCCGACCTCCAGTTGGAGCGTATGAACGTTTACTTCAACGAGGTGCGTTA---------------GAAAA
CAAGACAT-TCGACTCA-----ACCGGA---CAAAG-----------ACTCATCA--GTTG-----GTTTAGGCTTCCGGCAACAAATA
TGTTCCTCGTGCTGTTCTCGTCGACTTGGAGCCCGGNTCTCCCACCCTTGTCT-CTCTAT-ACCTGTTGCTTCGGC
GGGCCCACC-GGGACA--CTGGTCGCCGGGGGAC-G-TCCGTCCCCGGGCCCGCGCCCGCCGAAGCCCCCCTGT
GAACCCTGATGAATATGGAGCTGTCTGAGTATCAATG-AAAATCGTCAAAACTTTCAACAATGGATCTCTTGGTTCC
GGCATCGATGAAGAACGCAGCGAAATGCGATAAGTAATGTGAATTGCAGAATTCCGTGAATCATCGAATCTTTGAA
CGCACATTGCGCCCCCTGGCATTCCGGGGGGCATGCCTGTCCGAGCGTCATTTCTGCCCTCAAGCGCGGCTTG
TGTGTTGGGTGCGGTCCCCCC-GGGGACCCGCCCGAAAGGCAGCGGCGACGCCCGTCC-GGTCCTCGAGCGC
ATGGGGCTCTGTCACCCG-CTCGGGAAGGACCCGCGGGGGTTGGTCA

>Talaromyces_viridulus_CBS_252.87

TG-GATTTCTGG-TTGTC----------GCA----ATGTTG----TGT-GGGTAGTT-AGCTGACGAG-CCG---TTT---------TGATG-A
GTAGGACAAGGATGGAGATGGTGAGT---------------------GACCAC-----CACAAA--CACCGGCG-ATATGGTCTTGA-AC
CGAGG-------TTATT-ACT--------ACGAACA-TATATTGA---------CGGTATTTGAACAGGTCAAATCACAACCAAGGAACT
GGGCACCGTCATGCGTTCCCTCGGCCAGAACCCCTCTGAATCCGAATTGCAGGACATGATCAACGAGGTCGACG
CTGACAACAACGGCACAATCGATTTCCCTGGTATGACCCA-----------------ACACGTCT-ACGCTGAACACGA-----------

-TGGCGG----T-ACTAACTGC--C-A---CAGAATTCTTGACAATGATGGCCCGCAAAATGAAGGATACCGACTCCGAGG
AAGAGATCCGCGAGGCTTTCAAGGTGTTTGACCGCGACAACAATGNGTGAGTTTG--GTCTCG-AC--CCA------AAC
TTTC-TATCAATTGTCGCAAC-AACACGCTGACT-------C-TTTCAGGCAAATCATCTCTGCTGAGCACGGTCTCGACG
GCTCTGGTGTGTAA---------GTA-TC--GCACGA-TTCG-AATCCAGCTACAACCCGACA--A-TATCTGA-CAATCAACAG
CTACAATGGCTCCTCCGACCTCCAGTTGGAGCGTATGAACGTTTACTTCAACGAGGT----CA---------------GAC-----C
ACT-CCACCAGAT--AAATCG-A---ACAAA-----------ACTCAT-A--ATTG-----GTATAGGCCTCCGGCAACAAATATGTCCC
TCGTGCTATCCTCGTCGACTTGGAGCCCGGNCCTCCCACCCTTGTCT-CTATAC-ACCTGTTGCTTTGGCGGGCCC
ACC-GGGGCCACCTGGTCGCCGGGGGAC-G-TTCGTCCCCGGGCCCGCGCCCGCCGAAG-CGCCCTGTGAACC
CTGATGAAGATGG-GCTGTCTGAGTA-CGATG-AAAATTGTCAAAACTTTCAACAATGGATCTCTTGGTTCCGGCAT
CGATGAAGAACGCAGCGAAATGCGATAAGTAATGTGAATTGCAGAATTCCGTGAATCATCGAATCTTTGAACGCAC
ATTGCGCCCCCTGGCATTCCGGGGGGCATGCCTGTCCGAGCGTCATTTCTGCCCTCAAGCACGGCTTGTGTGTT
GGGTGTGGTCCCCCC-GGGGACCTGCCCGAAAGGCAGCGGCGACGTCCGTCG-GGTCCTCGAGCGCATGGGG
CTTTGTCACTCG-CTCGGGAAGGACCTGCGGGGGTTGGTCA
>Talaromyces_qii_AS3.15414
TG-GGTTTCCGG-TTGTC----------GCA----ATGTTG---TGGC-GGGTGGTT-AGCTGACTCG-CCG---TTT---------TGATG-
AGTAGGACAAGGATGGAGATGGTGAGT-------------------GAGCCGC-----AATGAT--CACCAACG-ATGCAGTCTTTG-
GACGAGT-------AAATC-AGC--------ACGAACAGTATCCTGA---------CTATATCTGAATAGGTCAAATCACAACCAAGGA
ACTGGGCACCGTCATGCGTTCCCTCGGCCAGAACCCCTCCGAATCCGAACTGCAGGACATGATCAACGAAGTCG
ACGCTGACAACAACGGCACAATCGATTTCCCTGGTATGACACA------------ATCATCTTACGGT-CGCTA-----CGA------
------TGGCAG----AGACTAACTGC--CTA---CAGAATTCCTGACAATGATGGCCCGCAAAATGAAGGATACCGACTCCG
AGGAAGAGATCCGTGAGGCTTTCAAGGTGTTTGACCGCGACAACAATGNGTGAGTGTTGACTCTCG-AC--CCA----
--AACTTTC-AGTCAATTCTCGCGAC-AACACGCTGACT------CT-TTCCAGGCAAATCATCTCCGCTGAGCACGGTCT
CGACGGCTCTGGTGTGTAA---------GTG-TT-GATACGA-ATCGAAATCC---TACAATTCGACA--C-TATCTGA-TAATCA
ACAGCTACAATGGCTCCTCCGACCTCCAGTTGGAGCGTATGAACGTTTACTTCAACGAGGTGCGTTA---------------
GAA----------TCACTCGACTCAAACGAAA--CACAGA------------CTCATCA--ATTG-----CA-TAGGCTTCCGGCAACAAGT
ATGTCCCTCGTGCTGTCCTCGTCGATTTGGAGCCCGGNCCTCCCACCCTTGTCT-CTATAT-ACCTGTTGCTTTGG
CGGGCCCACC-GGGGCCACCCGGTCGCCGGGGGAC-G-CTCGTCCCCGGGCCCGCGCCCGCCGAAG-CGCTCT
AAGAACCCTGATGAAGATGG-GCTGTCTGAGTA-ATTTG-AAAATTGTCAAAACTTTCAACAATGGATCTCTTGGTTC
CGGCATCGATGAAGAACGCAGCGAAATGCGATAAGTAATGTGAATTGCAGAATTCCGTGAATCATCGAATCTTTGA
ACGCACATTGCGCCCCCTGGCATTCCGGGGGGCATGCCTGTCCGAGCGTCATTTCTGCCCTCAAGCACGGCTT
GTGTGTTGGGTGTGGTCCCTCC-GGGGACCTGCCCGAAAGGCAGCGGCGACGTCCGTCT-GGTCCTCGAGCGTA
TGGGGCTCTGTCACTCG-CTCGGGAAGGACCTGCGGGGGTTGGTCA
>Talaromyces_fusisporus_AS3.15415
TG-GATTCCTGG-TTGTCGCGTTGTTGTGCA----ATGCTA---TAGC-GGGCGGTT-AGCTGACTAG-CCG---TTT---------T
GATG-AATAGGACAAGGATGGAGATGGTGAGT---------------------TTCTTACGAACACGAA--CGACAACG-ATACGATT
TTTG-GAGAATA-------TTATG-ACT--------ACGAACAACATATTGA---------TAAAGTC-CAATAGGTCAGATCACAACCAA
GGAACTGGGCACCGTCATGCGTTCCCTCGGCCAGAACCCCTCCGAATCTGAATTGCAGGACATGATCAACGAAG
TCGACGCTGACAACAACGGCACAATCGATTTCCCTGGTATGACAGT----------------ACACAAAATTGCAATTTTTGGA
------------GGGCAG----T-ACTAACTGC--C-G---CAGAATTCTTGACAATGATGGCCCGCAAAATGAAGGATACCGACTC
CGAGGAAGAAATCCGTGAGGCTTTCAAGGTGTTTGACCGTGACAACAATGNGTGAGTTGG-A--CACG-AC--CCA---
---AACTTTC-TATCAATTGTCGCGAC-AGCACGCTGACT---------TGTCAGGCAAATCATCTCTGCTGAGCACGGTCTC
GATGGCTCTGGTGTGTAA---------GTA-TT--ATACGA-TTCG-AATCCAGCTACAATCCGACA--G-TATCTGA-TCATCAA
CAGCTACAATGGCTCCTCCGACCTCCAGTTGGAGCGTATGAACGTCTACTTCAACGAGGTGCGTTA--------------G
GAT-----TTCC-TCGGCTCAT----GCAG-A---ACAAA----------CACTCAT----ATGA-------ATAGGCCTCCGGCAACAAATAC

GTCCCCCGTGCTGTCCTCGTCGACTTGGAGCCCGGNCCTCCCACCCTTGTCT-CTATAC-ACCTGTTGCTTTGGC
GGGCCCACCGGGGCTCACCTGGTCGCCGGGGGAC-G-TCTGTCCCCGGGCCCGCGCCCGCCGAAG-CGCTCTG
AGAACCCTGATGAAGATGG-GCTGTCTGAGTA-CTATG-AAAATTGTCAAAACTTTCAACAATGGATCTCTTGGTTCC
GGCATCGATGAAGAACGCAGCGAAATGCGATAAGTAATGTGAATTGCAGAATTCCGTGAATCATCGAATCTTTGAA
CGCACATTGCGCCCCCTGGCATTCCGGGGGGCATGCCTGTCCGAGCGTCATTTCTGCCCTCAAGCGCGGCTTG
TGTGTTGGGTGCGGTCCCCCC-GGGGACCTGCCCGAAAGGCAGCGGCGACGTCCGTTTGGGTCCTCGAGCGTA
TGGGGCTTTGTCACTCG-CTCGGGACGGACCCGCGGGGGTTGGTCA

>Talaromyces_helicus_CBS_335.48

TG-ACGGTTTGA----------------GCA----AGAATT--------GTGTGGC--AGCAATCTAA--TA---TTTTGATCTTGATGATG-TAC
AGGACAAAGATGGCGATGGTGAGTTTGTCGAGAGCATTTACTCCGAATTGCAGATAAGCAAT--CAACGAAGAAAA
AAGGGGTCG-ACATGGACACAAACCTGTG--------------GAATACTGTACTAAAAGCGTGATCGACGTT-GGCCAGGTC
AAATCACAACCAAAGAACTCGGTACCGTCATGCGCTCTCTCGGCCAGAACCCTTCCGAATCCGAATTGCAGGACA
TGATCAACGAAGTCGACGCTGATAACAACGGCACAATTGACTTTCCTGGTATGATCAA------------AATCCGTCCTCA
C-TCCCGCTGTTTTGCCAGCTCCCCCCTCGCAGTG--TGATTAATAGAAAGGGTATTAGAATTCTTGACCATGATGG
CTCGCAAAATGAAGGATACCGACTCCGAAGAAGAAATCCGCGAGGCATTCAAGGTGTTTGACCGCGACAACAAT
GNGTCCATCAT-ATTCTTG-TT-------------CCCTC-TGCTAATCGTCATAAC-------------------------CAGGCAAATCATCTCT
GCTGAGCACGGCCTCGATGGCTCTGGTGTGTAAGTTGACCGTGTATTC--TGTCGACGTCGAAATCTATCATCGAT
CGCTCA--G-ATTCTGACCAACCACCAGCTACAATGGCTCCTCCGACCTTCAATTGGAGCGCATGAACGTCTACTTC
AACGAGGTACGCCAATCCATCTAAGGCCTACACGCATTCAAT-CCAACTAACGCGAGAAAAA---AAAAA----------------
--------------TCTTAGGCCAACGGCAACAAATATGTTCCTCGTGCTGTCCTCGTCGATCTCGAGCCCGGNCCTCCCAC
CCGTGCCGTTATAAT-ACCTGTTGCTTTGGCGGGCCCAC--GGGG-CACCTGGTCGCTGGGGGAC-G--CCGTCCCC
GGGCCCGCGCCCGCCGAAG-CACCCCCTGAACTCTGAAGAAGATAGGGCTGTCTGAG-A-CCTAG-AAAATTGTCA
AAACTTTCAACAATGGATCTCTTGGTTCCGGCATCGATGAAGAACGCAGCGAAATGCGATAAGTAATGTGAATTGC
AGAATTCCGTGAATCATCGAATCTTTGAACGCACATTGCGCCCCCTGGCATTCCGGGGGGCATGCCTGTCCGAG
CGTCATTTCTGCCCTCAAGCACGGCTTGTGTGTTGGGTGTGGTCCCCCCGGGGGACCTGCCCCAAAGGCAGCG
GCGACGCCCGTCG-GGTCCTCGAGCGCATGGGGCTT-GTCACTTG-CTCGGGAGGGACCTGCGGGGGTTGGTCA

Data S4. The sequence alignment of combined *CaM*, *BenA* and *ITS1-5.8S-ITS2.*
